# Cost-utility analysis of teriflunomide in naïve vs. previously treated patients with relapsing–remitting multiple sclerosis in Italy

**DOI:** 10.1007/s10072-022-06022-x

**Published:** 2022-04-14

**Authors:** Carlo Lazzaro, Roberto Bergamaschi, Mauro Zaffaroni, Rocco Totaro, Damiano Paolicelli

**Affiliations:** 1 Studio di Economia Sanitaria, Via Stefanardo da Vimercate, 19, 20128 Milan, Italy; 2 IRCCS Mondino Foundation, Pavia, Italy; 3 Multiple Sclerosis Centre, Hospital of Gallarate, ASST Della Valle Olona, Gallarate, Italy; 4 Centro Malattie Demielinizzanti, Ospedale San Salvatore, L’Aquila, Italy; 5 Department of Basic Medical Sciences, Neuroscience and Sense Organs, University of Bari “Aldo Moro”, Bari, Italy

**Keywords:** Relapsing–remitting multiple sclerosis, Teriflunomide, Disease-modifying treatment, Cost-utility analysis, Markov model, Italy

## Abstract

**Background:**

Multiple sclerosis (MS) accounts for 176 cases per 100,000 inhabitants (female/male ratio = 2:1) in Italy. For most of the patients (67%), the disease course is relapsing–remitting MS (RRMS).

**Objective:**

To compare the costs and quality-adjusted life years (QALYs) of teriflunomide in RRMS naïve patients vs. RRMS patients previously treated (experienced) with other disease-modifying therapies in Italy.

**Methods:**

A four health states Markov model-supported cost-utility analysis (CUA) covering a 7-year timespan through annual cycles was developed, following the healthcare sector and the societal viewpoints. Part of the parameters that populated the Markov model was obtained from a questionnaire administered to four primary Italian MS centres. Costs of healthcare and non-healthcare resources, expressed in euro (€) 2019, and QALYs were discounted at 3% real social discount rate. One-way, scenario and probabilistic sensitivity analyses tested the uncertainty of the baseline findings.

**Results:**

Baseline CUA shows that teriflunomide in RRMS naïve patients is strongly dominant vs. experienced patients (healthcare sector perspective: − €1042.68 and + 0.480 QALYs; societal perspective: − €6782.81 and + 0.480 QALYs). Sensitivity analyses confirmed the robustness of the baseline results.

**Conclusion:**

Teriflunomide in RRMS naïve vs. experienced patients is cost-effective and possibly strongly dominant from both the healthcare sector and the society viewpoints in Italy. Our findings need further confirmation from real-world studies.

**Supplementary Information:**

The online version contains supplementary material available at 10.1007/s10072-022-06022-x.

## Introduction

Multiple sclerosis (MS) is a chronic debilitating neurological disorder, accounting for 176 cases per 100,000 inhabitants (female/male ratio = 2:1) in Italy [1, 2].

For most of the patients (67%), the disease course is relapsing–remitting MS (RRMS) [2].

MS implies a sustained consumption of healthcare resources, reduces patients’ productivity, worsens her/his health-related quality of life (utility) [3] and often requires informal care [3].

In recent years, the treatment paradigms of MS have profoundly changed, due to the availability of a wide spectrum of disease modifying therapies (DMTs), acting on the immune system with distinct mechanisms of action. These also include oral therapies, such as teriflunomide, dimethyl-fumarate, fingolimod, cladribine often used in RRMS patients; thanks to their proven efficacy and safety, also being able to enhance patients’ compliance [4].

From a recent survey of the literature, the oral agents appear to be generally well tolerated with an acceptable safety profile [5]. However, this does not exclude that there may be more subtle and specific issues with each of the different drugs [5].

 Most patients with MS switch between DMTs during their lifetime because non-responders or for safety concerns. The choice of the optimal treatment has therefore become increasingly complex, having as its first goal the ability to prevent the accumulation of disability over time [6].

As it was proved to reduce relapses and delay disability progression [7–9], since 2014 teriflunomide (Aubagio® — Sanofi Srl, Italy) 14 mg per os once per day has been reimbursed by the Italian National Health Service (INHS) as first line DMT for RRMS [10]. However, it was quite often used after the patient had already tried a different DMT, even for convenience reasons [11, 12].

The development of new drugs in MS has led to an increase in costs for the management of the disease. In this scenario, economic evaluation of healthcare programmes, in addition to clinical indication, plays a relevant role in supporting resource allocation decision-making.

This study presents a cost-utility analysis (CUA) [3] (Supplementary Information-SI Definition 1) that, adopting both the healthcare sector and the societal viewpoints, aims at verifying whether privileging the use of teriflunomide in RRMS patients who did not receive any previous therapy (naïve) vs. RRMS patients who had been already treated (experienced) with up to three DMTs is also cost-effective in Italy.

## Materials and methods

### Markov model

CUA was supported by a Markov model with four mutually exclusive and exhaustive health states (controlled RRMS: health state when patient enters the model; RRMS relapse; RRMS remission; all-cause mortality) (Fig. SI1) [3, 13, 14].

The Markov model lasts 7 years (i.e. 7 1-year Markov cycles) as it does not consider the possible conversion from RRMS to secondary progressive (SP) MS, since teriflunomide has no therapeutic indication for SPMS [10].

At each Markov cycle, two hypotethical cohorts of 1000 RRMS naïve and 1000 RRMS experienced patients remain in the same Markov state, progress to another Markov state or die for age- and gender-specific all-cause mortality, according to a transition probability matrix calculated on a subset of 721 parameters detailed separately (Tables SI1-SI56) [3, 13, 14].

Parts of the clinical, demographic and economic parameters that populated the Markov model (266/721 = 36.89%) were obtained from a questionnaire [15] administered by in-presence (50%) or phone (50%) interviews during June 2019 to a convenient sample [16] of 4 neurologists expert in MS (approximately 5000 patients followed-up per year in total) representative of as many primary MS centres in the north, middle and south Italy, as well as from research assumptions and literature.

The Expanded Disability Status Scale (EDSS) score-specific probability of relapse was obtained from the literature [17, 18].

The probability of remission after a clinical relapse was set at 20% (Table SI31) [19].

According to neurologists’ qualified opinion [15], from year 2 onwards, 10% RRMS naïve patients who relapsed were assumed to switch to second line therapies as alemtuzumab, cladibrine, fingolimod, natalizumab or ocrelizumab due to teriflunomide ineffectiveness (Table SI39).

Adherence to teriflunomide that was expected to influence both cost and quality-adjusted life years (QALYs) [3] was assumed 100% for both the hypothetical cohorts of patients.

For each Markov state, QALYs were calculated by multiplying life-years saved (LYS) by utility (Table SI38) [3]. Utility values related to EDSS score and disutility values for possible adverse events (AEs) related to teriflunomide were retrieved from previous researches [17, 20–24].

As this research did not imply patients’enrollment, no ethics committee approval of the study protocol (included the abovementioned questionnaire) was required by the Italian legislation [25].

The Markov model was developed with software Microsoft® Excel® for Windows® 2010 (Microsoft Corporation, Seattle, WA, USA).

### Healthcare and non-healthcare resources identification, quantification and valuation

Both healthcare and non-healthcare resources were considered. Healthcare resources included teriflunomide and other DMTs (alemtuzumab; cladribine; fingolimod; natalizumab; ocrelizumab); for alemtuzumab, natalizumab, ocrelizumab, healthcare professionals’ time, drugs and disposables for premedication, administration and postemedication in outpatient or day-hospital setting; patients assessment before and during treatment with teriflunomide and other DMTs; drugs and healthcare procedures to manage relapses and possible AEs due to teriflunomide; patients follow-ups (with and without relapses); mobility aids (walking canes; walkers; crutches; wheelchairs) (Tables SI1-SI19; SI23-SI30; SI32-SI37; SI39-SI49).

Being the same for both the hypothetical cohorts of patients, diagnosis-related healthcare resources were not considered.

Teriflunomide and other DMTs were costed at ex-factory price (net of mandatory discounts) [10, 26] as they are administered in hospital setting only; other drugs were costed at retail price as they can be purchased at territorial pharmacy.

Healthcare procedures were valued according to the INHS tariffs for day-hospital and outpatient setting, which were assumed to be good proxies for their opportunity cost (i.e. the cost of the best forgone alternative) (Tables SI20; SI50-SI52) [27].

Usually, INHS tariffs for drugs administration include the cost of drugs. Since teriflunomide and other DMTs were valued separately, cost related to their administration in day-hospital or outpatient setting was halved to avoid double counting (i.e. costing the same resource twice) [3, 24].

The yearly cost of disability aids was obtained from the literature (Table SI52).

Non-healthcare resources included car transportation back and forth between home and hospital; car parking at the hospital; patient and caregiver’s time (working hours lost for patients aged < 70; leisure hours lost for patients aged ≥ 70 and for all the caregivers) for transportation, assessments, therapy or follow-ups; patients’ home and car adaptations (Tables SI9; SI13; SI22; SI48; SI49).

Non-healthcare resources were valued via unit monetary standards (Table SI52). The loss of working and leisure hours was costed via the gross wage rate (net wage + income taxes + social security contributions) and take-home wage rate (net wage only) approaches, respectively [28]. The loss of working hours experienced by housekeepers affected by RRMS was valued at homehelper hourly gross wage [3].

Resources were grouped into two different cost categories [3]. Healthcare sector costs included all the healthcare resources funded by INHS or hospital. Patient and/or her/his family costs included all non-healthcare resources plus healthcare resources not funded by INHS (e.g. over the counter drugs for hair thinning and rachialgy) (Tables SI21; SI52).

Costs were expressed in euros (€) at 2019 values and inflated to that year when necessary.

Costs, LYS and QALYs were discounted at 3% real social discount rate [3, 29] and half-cycle correction was applied (i.e. death was assumed to occurr half-way between the annual Markov cycle, so that dead notional patients were assigned 6-month costs, LYS and QALYs) [13, 14].

### Statistical analysis

The number of notional patients in each Markov state was reported as mean and standard deviation (SD).

The majority of the parameters that populated the Markov model (438/721 = 60.75%) were assigned a theoretical probability distribution [14].

The beta distribution was fitted to dichotomous events (i.e. events that imply 2 pathways), such as probabilities and RRMS stage-specific utility values.

Polytomous events (i.e. events that imply ≥ 3 pathways), such as conditional probabilities of switching to other DMTs given the ineffectiveness of teriflunomide, followed a Dirichlet distribution.

The gamma distribution was fitted to volume of healthcare resources consumption (if different from drugs posology) and EDSS scores.

The normal distribution was assigned to the unit cost of healthcare resources different from drugs.

Point estimate and 95% confidence interval (95% *CI*) were calculated for all the parameters which were given a statistical distribution [14].

Parameter standard error was determined analytically or by imposing an appropriate coefficient of variation on the sample mean (Tables SI40-SI52) [14].

For parameters which were not assigned a theoretical probability distribution point estimate and range were reported.

No hypothesis test was performed on costs, LYS and QALYs totalled by RRMS naïve or experienced patients.

### Sensitivity analyses

One-way (OW), scenario (S) and probabilistic (P) sensitivity analyses (SA) tested the robustness of the baseline ICUR [3].

Parameters included in OWSA were varied one at a time, holding the others at their base case values [3]; parameter baseline point estimates were replaced with the limits of the range or the 95% *CI* of the assigned theoretical probability distributions [14, 24].

OWSA explored the variations in ICUR due to changes in all the parameters related to event probabilities; resource consumption; unit cost; utility and disutility values (Tables SI40-SI52).

The 3% baseline real social discount rate was also changed (0%; 5%) [29] to check its influence on the base case findings.

OWSA results are displayed as departures from the basecase ICUR on a tornado chart and in tables.

The first SSA provided the annual point estimates of the ICUR during the 7-year timespan the Markov model stretches over [3], in order to investigate the relationship between ICUR and time and its potential bearing on the cost-effectiveness profile of the healthcare technologies under comparison.

The second and third SSA assessed the impact on cost and QALYs due to a lower adherence probability to teriflunomide (from 90 to 50%) and a higher probability of recovery after RRMS relapse (from 30 to 90%) for both the hypothetical cohorts of patients.

PSA explored the parameters joint uncertainty via a 10,000-iteration Monte Carlo simulation [3, 14]. For each Monte Carlo iteration, a random value was drawn for each one of the 438 parameters which were fitted a statistical distribution (the remaining 283 parameters entered the PSA at their base case estimate), so that 10,000 ICURs were simulated [3, 14].

An algebraic manipulation of the ICUR (net monetary benefit (NMB)) (SI Definition 2) supported the construction of cost-effectiveness acceptability curve (CEAC) and cost-effectiveness acceptability frontier (CEAF) (SI Definitions 3 and 4) [3, 14, 30, 31].

Averaging over the results of the Monte Carlo simulation, PSA showed the probability that one of the alternatives was cost-effective (CEAC) or optimal (CEAF), that is having the highest expected NMB vs. comparator for different threshold values [3, 14, 30, 31].

CEAC and CEAF overlap if and only if the healthcare programme showing the higher probability of being cost-effective has also the highest expected NMB [3, 14, 30, 31].

As recommended by literature, posology, number of administrations and unit costs of drugs were excluded from SA [3, 14].

## Results

### Markov model

On average, RRMS naïve enter the model at 33 years (range: 25–49), being younger than RRMS-experienced notional patients (37 years; range: 25–48). The first administration of teriflunomide occurs after 6 years (range: 1–13) and 11 years (range: 8–13) from diagnosis for RRMS naïve and experienced notional patients, respectively.

Female patients were 78.71% and 77.08% for RRMS naïve and experienced notional patients, respectively.

During the 7-year timespan (Table 1),

**Table 1:** Results — base case analysis — mean (SD) number of notional patients in each Markov state

Markov states	RRMS naïve patients	%	RRMS experienced patients	%
Controlled RRMS				
Teriflunomide — non-relapsed RRMS	291 (115)	29.09%	282 (145)	28.23%
Relapse				
Teriflunomide — RRMS relapse	511 (61)	51.12%	608 (111)	60.76%
Switch from teriflunomide to other DMTs^a^ — RRMS relapse	73 (46)	7.27%	0.00	0.00%
Remission				
Teriflunomide — remission after RRMS relapse	86 (39)	8.57%	107 (51)	10.73%
Switch from teriflunomide to other DMTs — remission after RRMS relapse	37 (38)	3.73%	0.00 (-)	0.00%
Absorption state				
Death	2 (1)	0.22%	3 (2)	0.28%
Total	**1000**	**100.00**	**1000**	**100.00**

DMT, disease-modifying treatment; SD, standard deviation; RRMS, relapsing–remitting multiple sclerosis

^a^DMTs, alemtuzumab; cladribine; fingolimod; natalizumab; ocrelizumab

the RRMS naïve hypothetical cohort reports, on average, 291 (*SD*: 115) non-relapsed patients (29.09% of the starting 1000 notional patients), 511 (*SD*: 61) patients who relapsed during teriflunomide treatment (51.12%) and 73 (*SD*: 46) who relapsed after switching from teriflunomide to other DMT treatment (7.27%); 86 (*SD*: 39) patients who recovered after a relapse during teriflunomide treatment (8.57%) and 37 (*SD*: 38) patients who recovered after a relapse during DMT treatment (3.73%); eventually, 2 (*SD*: 1) patients dead (0.22%).

The RRMS experienced hypothetical cohort totals, on average, 282 (*SD*: 145) non-relapsed patients (28.23% of the starting 1000 notional patients), 608 (*SD*: 111) patients who relapsed during teriflunomide treatment (60.76%); 107 (*SD*: 51) patients who recovered after a relapse during teriflunomide treatment (10.73%); finally, 3 (*SD*: 2) patients passed away (0.28%).

Loss of working or leisure time is higher for RRMS naïve patients (205.44 h; range: 169.47–238.61 vs. 181.92 h; range: 156.48–214.06), who, in turn, need less informal care (37.20 h; range: 20.95–57.81 vs. 83.28 h; range: 63.49–105.17).

### Cost, LYS, QALYs and incremental cost-utility ratio

Following the societal perspective, after 7 years, the average cost per notional patient is lower for RRMS naïve vs. experienced patients (€108,162.69 vs. €114,945.50) (Table 2).

**Table 2:** Results — base case analysis — cost per patient (€2019)

Cost items					RRMS naïve patients	%		RRMS experienced patients	%	
Healthcare sector costs										
Patient assessment before teriflunomide first administration					€113.34	0.10%		€113.34	0.10%	
Teriflunomide					€64,168.16	59.33%		€77,461.03	67.39%	
Liver function monitoring during teriflunomide treatment					€105.09	0.10%		€123.15	0.11%	
Management of teriflunomide AEs					€64.83	0.06%		€61.24	0.05%	
Switches from teriflunomide to other DMTs^a^										
Pre-treatment assessment					€2.63	0.002%		€0.00	0.00%	
On treatment assessment					€84.55	0.08%		€0.00	0.00%	
Premedication					€9.49	0.01%		€0.00	0.00%	
Drugs + administration					€16,838.13	15.57%		€0.00	0.00%	
Postmedication					€42.32	0.04%		€0.00	0.00%	
Follow-up in case of no RRMS relapse					€1359.15	1.26%		€906.74	0.79%	
RRMS relapse management					€943.17	0.87%		€986.68	0.86%	
Post RRMS relapse follow-up					€616.69	0.57%		€645.04	0.56%	
Mobility aids					€5326.69	4.92%		€10,419.72	9.06%	
Total (A)					**€89,674.24**	**82.91%**		**€90,716.92**	**78.92%**	
Patients and their family cost — out-of-pocket expenses										
Management of teriflunomide AEs^b^					€0.39	0.0004%		€0.43	0.0004%	
Mobility aids^b^					€3162.24	2.92%		€6095.75	5.30%	
Other aids					€3163.42	2.92%		€6098.02	5.31%	
Trasportation					€4844.04	4.48%		€4560.41	3.97%	
Parking					€290.92	0.27%		€291.33	0.25%	
Total (B)					**€11,461.01**	**10.59%**		**€17,045.94**	**14.83%**	
Patients and their family cost — patient time and caregiver’s time										
Patient loss of working time^c^					€6527.93	6.04%		€6066.12	5.28%	
Informal care^c^					€499.50	0.46%		€1116.52	0.97%	
Total (C)					**€7027.44**	**6.50%**		**€7182.64**	**6.25%**	
Overall (A + B + C)					**€108,162.69**	**100.00%**		**€114,945.50**	**100.00%**	
Base case analysis — cost-utility analysis (€2019)										
Healthcare programmes	Cost		LYS	QALYS	**Incremental** **cost (ΔC)**			**Incremental QALYs (ΔQALYs)**	**ICUR** **(ΔC/ΔQALYs)**	
Healthcare sector perspective										
RRMS experienced patients	€90,716.92	6.402		3.123	**-**			**-**	**-**	
RRMS naïve patients	€89,674.24	6.406		3.603	− €1042.68			0.480	Teriflunomide in RRMS naïve patients is strongly dominant vs RRMS-experienced patients	
Societal perspective										
RRMS experienced patients	€114,945.50	6.402		3.123	**-**		**-**			**-**
RRMS naïve patients	€108,162.69	6.406		3.603	− €6782.81		0.480			Teriflunomide in RRMS naïve patients is strongly dominant vs RRMS-experienced patients

*AE*, adverse event; *DMT*, disease-modifying treatment; *ICUR*, incremental cost-utility ratio; *INHS*, Italian National Health Service; *LYS*, life-years saved; *QALYs*, quality-adjusted life years; *RRMS*, relapsing–remitting multiple sclerosis

^a^DMTs: alemtuzumab; cladribine; fingolimod; natalizumab; ocrelizumab

^b^Healthcare resources not funded by INHS

^c^For patients ≥ 70 years old and all the caregivers the loss of working time has been valued as loss of leisure time

Healthcare sector costs amount to €89,674.24 and €90,716.92 for RRMS naïve and experienced notional patients (82.91% vs. 78.92% of the overall cost, respectively); out-of-pocket expenses equal €11,461.01 and €17,045.94 for RRMS naïve and experienced notional patients (10.59% vs. 14.83% of the overall cost, respectively), whereas productivity losses and informal care reach €7027.44 and €7182.64 for RRMS naïve and experienced notional patients (6.50% vs. 6.25% of the overall cost, respectively).

For both the hypotetical cohorts, the cost-driver is teriflunomide (59.33% and 67.39% of the overall cost totalized by RRMS naïve and experienced notional patients, respectively).

Despite the similarity in overall mortality (LYS 6.406 vs. 6.402 for RRMS naïve and experienced notional patients, respectively), 7-year QALYs are higher for RRMS naïve patients (3.603 vs. 3.123 per notional patient).

Due to incremental QALYs (+ 0.480 for both perspectives) and cost-savings (healthcare sector perspective: − €1042.68; societal perspective: − €6782.81), teriflunomide is strongly dominant when administered to RRMS naïve vs. experienced notional patients.

### Sensitivity analyses

**Fig. 1:**
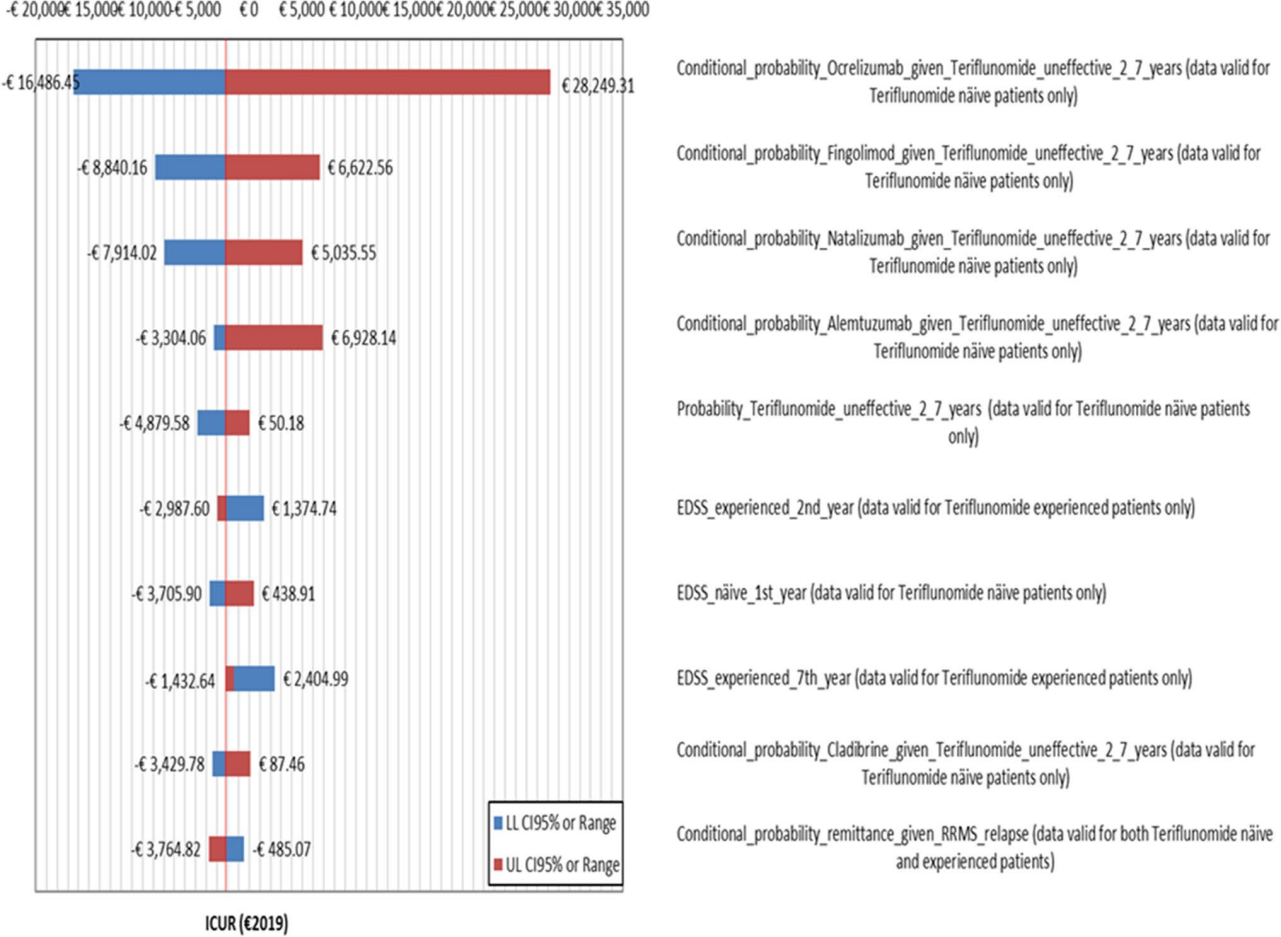
Results — OWSA — tornado chart — departures from baseline ICUR caused by the most relevant 10 parameters included in OWSA — healthcare sector perspective (€2019). CI, confidence interval; EDSS, Expanded Disability Status Scale; ICUR, incremental cost-utility ratio; LL 95% *CI*, lower limit 95% *CI*; OWSA, one-way sensitivity analysis; RRMS, relapsing–remitting multiple sclerosis; UL 95% *CI*, upper limit 95% *CI*. Base case ICUR, teriflunomide in RRMS naïve patient is strongly dominant

**Fig. 2:**
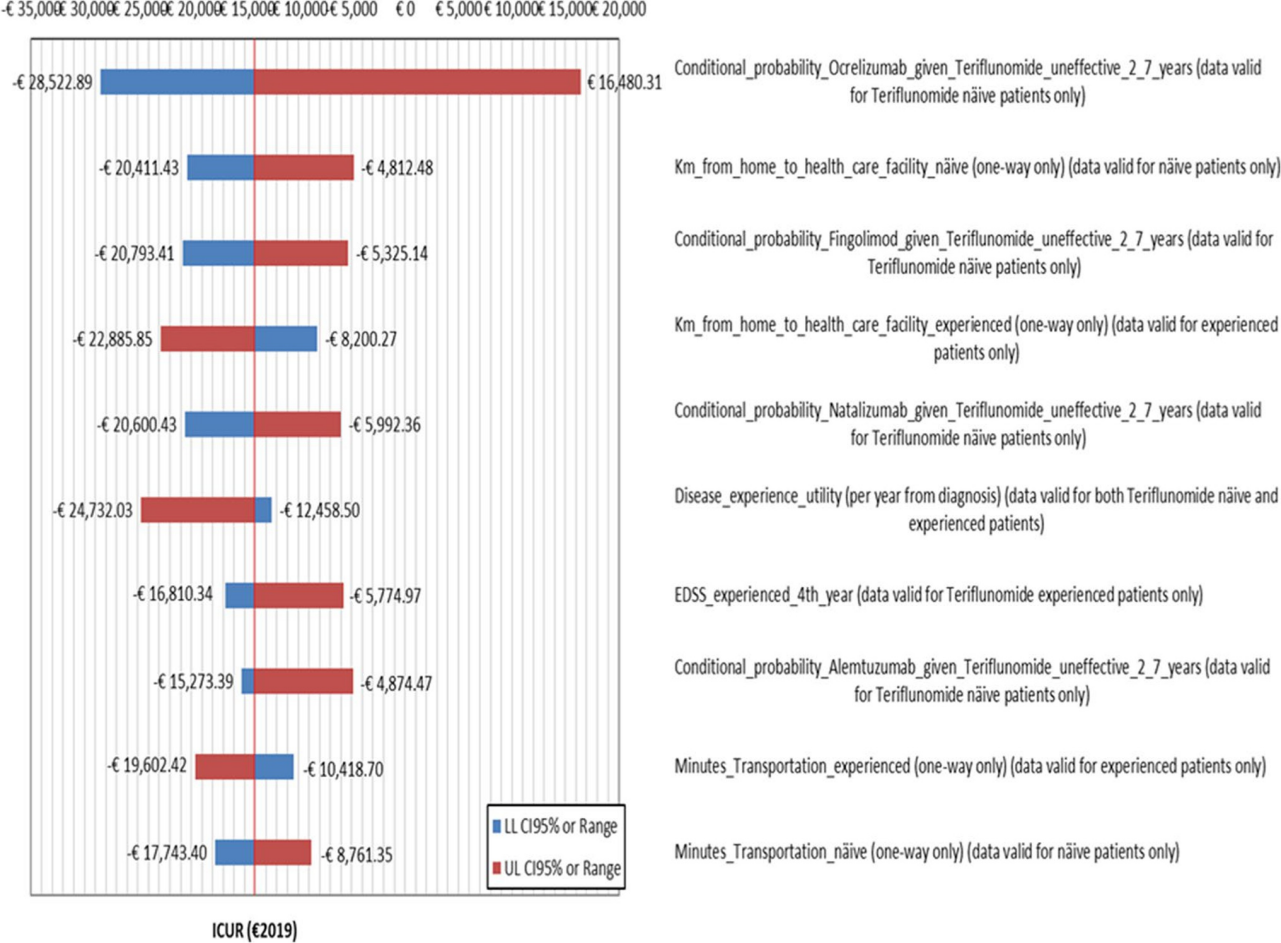
Results — OWSA — Tornado chart — departures from baseline ICUR caused by the most relevant 10 parameters included in OWSA — societal perspective (€2019). CI, confidence interval; EDSS, Expanded Disability Status Scale; ICUR, incremental cost-utility ratio; LL 95% *CI*, lower limit 95% *CI*; OWSA, one-way sensitivity analysis; RRMS, relapsing–remitting multiple sclerosis; UL 95% *CI*, upper limit 95% *CI*. Base case ICUR, teriflunomide in RRMS naïve patient is strongly dominant

For both healthcare sector and societal viewpoints, OWSA shows that the largest departures from baseline ICUR reported on tornado chart follow from variations in the conditional probability of switching to ocrelizumab due teriflunomide ineffectiveness in RRMS naïve notional patients (from − 1401.30 to + 659.45% vs. base case ICUR) (Figs. 1 and 2).

The conditional probability of switching to fingolimod in RRMS naïve notional patients given poor response to teriflunomide is ranked second (healthcare sector perspective: from − 405.07 to + 307.22%) and third (societal perspective: from − 62.29 to + 47.24%) among the most influential parameters on the base case cost per incremental QALY gained when its sample estimate is replaced by the lower and the upper limits of 95% *CI* (from − 405.07 to + 307.22%).

Changes in EDSS scores totalled by RRMS-experienced notional patients produce a moderate variation in the baseline ICUR when the healthcare sector standpoint is considered (from − 210.79 to + 37.62%) (Fig. 1).

The mildest impact on the base case ICUR is due to variations in the conditional probability of remission given relapse for both RRMS naïve and experienced notional patients (healthcare sector perspective: from − 77.66 to + 73.43%) and minutes of transportation from RRMS naïve patient’s home to hospital (societal perspective: from − 37.96 to + 25.65%), respectively (Figs. 1 and 2).

Real social discount rate for costs, LYS and QALYs is not ranked among the first 600 (healthcare sector perspective) and 30 (societal perspective) parameters that produce the largest variations in the baseline ICUR.

In the first SSA, ICUR reaches its maximum at year 3 for both healthcare sector (€26,422.27) and societal (€30,661.47) standpoints and then decreases progressively; the strong dominance status for RRMS naïve notional patients is reached at year 7 (Fig. SI2).

The second SSA highlights that reducing adherence probability to 40% increases baseline ICUR of + 1762.31% (health case sector perspective) and + 327.44% (societal perspective) (Table SI53).

Notwithstanding the increased probability of remission after RRMS relapse, the third SSA confirms that teriflunomide in RRMS naïve notional patients is strongly dominant (Table SI54).

**Fig. 3:**
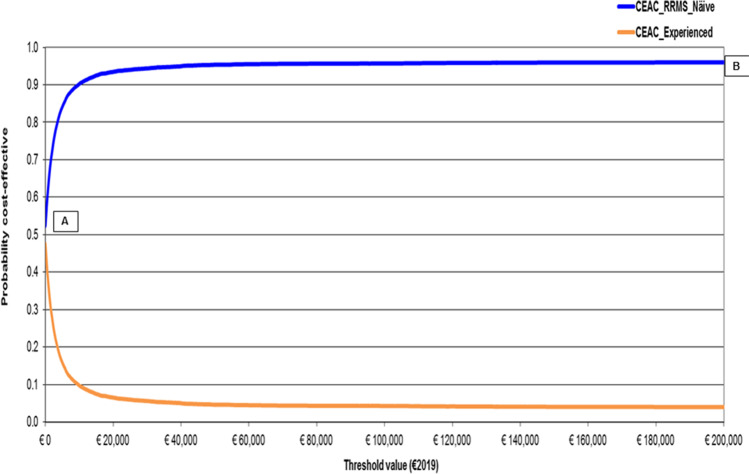
Results — PSA — CEAC and CEAF (1000 out of 1000 threshold values plotted) — healthcare sector perspective (€2019). CEAC, cost-effectiveness acceptability curve; CEAF, cost-effectiveness acceptability frontier; ICUR, incremental cost-utility ratio; NMB, net monetary benefit; PSA, probabilistic sensitivity analysis; RRMS, relapsing–remitting multiple sclerosis; WTP, willingness to pay. Base case ICUR, teriflunomide in RRMS naïve patients is strongly dominant. CEAC for teriflunomide in RRMS naïve patients and CEAF overlap as it is the healthcare programme with the higher probability of being cost-effective and the highest expected NMB CEAF shows that teriflunomide in RRMS naïve patients is the optimal strategy from a threshold value of €0.00 onward (i.e. from A to B)

**Fig. 4:**
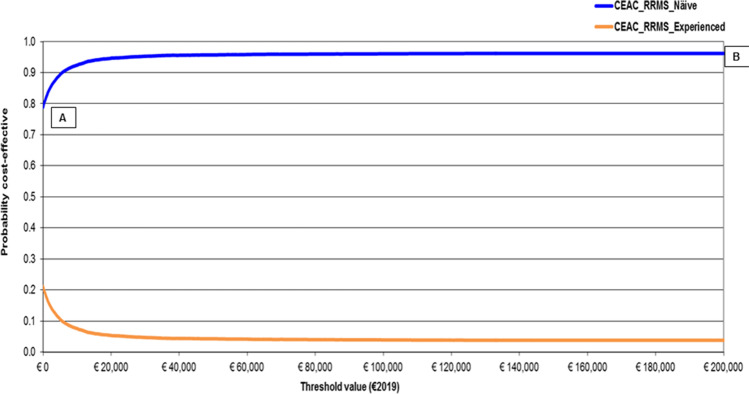
Results — PSA — CEAC and CEAF (1000 out of 1000 threshold values plotted) — societal perspective (€2019). CEAC, cost-effectiveness acceptability curve; CEAF, cost-effectiveness acceptability frontier; ICUR, incremental cost-utility ratio; NMB, net monetary benefit; PSA, probabilistic sensitivity analysis; RRMS, relapsing–remitting multiple sclerosis; WTP, willingness to pay. Base case ICUR, teriflunomide in RRMS naïve patients is strongly dominant. CEAC for teriflunomide in RRMS naïve patients and CEAF for RRMS naïve patients overlap as it is the healthcare programme with the higher probability of being cost-effective and the highest expected NMB. CEAF shows that teriflunomide in RRMS naïve patients is the optimal strategy from a threshold value of €0.00 onward (i.e. from A to B)

As far as PSA results are concerned, CEAC shows higher probability for teriflunomide in RRMS naïve notional patients to be cost-effective as the willingness to pay (WTP) for incremental QALY gained increases. Following the healthcare sector (societal) perspective, the likelihood for teriflunomide in RRMS naïve notional patients to be cost-effective equals 94.52% (95.46%) for a WTP of €25,000 and 95.37% (95.89%) for a WTP of €40,000 (Figs. 3 and 4).

If, being interested in savings only, decision-makers assigned a WTP = 0 per incremental QALY gained, the probability for teriflunomide to be cost-effective in RRMS naïve notional patients would be 52.30% (healthcare sector standpoint) and 79.75% (societal standpoint), respectively.

For both healthcare sector and societal viewpoints, the CEAC of teriflunomide in RRMS naïve patients and CEAF overlap as it is the healthcare programme with the higher probability of being cost-effective and the highest expected NMB from a WTP = 0 onward.

## Discussion

This paper reports on methods and results of a 7-year Markov model-supported CUA [3, 13, 14] that compares costs and QALYs totaled by two hypothetical cohorts of RRMS patients on teriflunomide who are, respectively, naïve and experienced vs. previous DMTs.

To the best of our knowledge, our research is innovative in two respects.

First, this is the first comparison of costs and QALYs accrued to RRMS notional patients on teriflunomide who were already or never exposed to DMTs.

Besides, the Markov model-supported CUA was conceived from scratch for the Italian setting instead of being a customization to the local clinical and economic standards of a health economic model originally developed for a different country.

Despite previous researches did not provide consistent recommedations about the cost-effectiveness of teriflunomide [32–36], the basecase analysis and most of the variations made in SAs show that, for both healthcare sector and society standpoints, teriflunomide administered in RRMS naïve rather than experienced patients is cost-saving and, at the same time, produces more QALYs.

OWSA highlights that, for both healthcare sector and societal perspectives, the baseline ICUR is highly sensitive to variations induced in the conditional probability of switching to other DMTs from year 2 onwards given teriflunomide ineffectiveness in RRMS naïve notional patients. This finding is consistent with the remarkable impact of the DMTs (15.57%) on the overall cost totaled by RRMS naïve notional patients in the base case CUA. In addition, since MS experts’ point estimate for this parameter (10.00%) was less optimistic than the mean probability of discontinuation due to ineffectiveness of teriflunomide 7 mg (3.86%) and 14 mg (2.57%) reported in literature (3.86% + 2.57% = 6.43%) [8], the favourable cost-effectiveness profile of teriflunomide in RRMS naïve vs. experienced patients was possibly conservative.

Despite remarkable variations in Markov model key-parameters, such as time horizon, adherence probability to teriflunomide and remission after RRMS relapse, SSAs confirm the baseline results or, at worst, show the base case ICUR not to exceed the upper limit of the informal acceptability range for incremental LYS or QALY gained suggested for the Italian setting (€25,000–€40,000) [29].

PSA shows that there is no evidence that costs of RRMS-experienced patients on teriflunomide may be lower than the ones totalized by their naïve counterparts.

This finding is proved by the intersection of CEACs with the *y*-axis that represents the one-side *p*-value for the difference in costs between the two healthcare programmes under comparison, as for WTP = 0 cost containment only is important for decision-makers [14]. These values (52.30% and 79.75% for healthcare sector and societal perspectives, respectively) are higher than the arbitrary 5% one-way *p*-value.

Conversely, CEACs highlight that the difference in QALYs for RRMS naïve patients on teriflunomide is statistically significant at 5%. This result becomes apparent as, when WTP approaches positive infinity, CEACs tend to 1 minus the (one-tailed) *p*-value for the difference in QALYs gained by RRMS naïve patients, that is (1–96.52%) = 3.48% for both the adopted viewpoints [14].

More substantively for rationing decisions, CEACs show that, when contrasted against the aforementioned informal acceptability range for incremental LYS or QALY gained recommended for Italy [29], the probability for teriflunomide to be cost-effective in RRMS naïve patients is always higher than 94.50%, regardless of the adopted standpoint. CEAFs confirm CEACs results.

PSA findings can impact Italian MS specialists’ decision-making, whose clinical practice faces tight budget constraints. Prescribing teriflunomide RRMS in naïve patients implies a negligible likelihood of resources misallotment that can be calculated as the difference between 1 and the probability for teriflunomide in RRMS naïve patients to be optimal for each WTP value reported on the *x*-axis of the CEAF graph. For instance, following the healthcare sector viewpoint, the probability of resources misallocation reaches (1–94.52%) = 5.48% and (1–95.37%) = 4.63% for a WTP of €25,000 and €40,000 [29], respectively.

### What are the main limitations of this study?

First, as a head-to-head comparison of teriflunomide in naïve vs. experienced RRMS real patients was unavailable, the parameters needed for populating the Markov model were retrieved from different sources [37]. In addition, a relevant share of the clinical parameters was obtained from the qualified opinion [15] of a convenience sample [16] of 4 MS specialists who coauthored this manuscript. It is also worth noting that, since all the authors gathered together in a meeting aimed at exploring the feasibility of this research, the Delphi panel approach [15] that elicits data from each expert separately was not feasible, as the anonymity requirement would have not been met.

Against the possible criticism that the only robust findings are those obtained from empirical economic evaluation of healthcare programmes piggybacked onto clinical trials [3], it seems worth reminding that healthcare resources allocation based on the results of a Markov model-supported CUA are, in all likelihood, more helpful than decisions made with no support at all [38].

A third limitation, linked to the first one, rests on the evidence that utility values related to different EDSS scores [17] and disutility values due to the adverse events that teriflunomide may cause [20–24] were originally collected for foreign countries. However, the literature claims that, unlike costs, QALYs are less expected to change remakably when utility values are retrieved from researches performed abroad, as, other things being equal, patients sharing the same disease severity tend to report similar utility values [31].

Notwithstanding, it would be interesting to collect empirical utiliy (and disutility) values from a sample of RRMS naïve and experienced patients on teriflunomide in a CUA performed alongside an Italian empirical study [39]. This chance will be particularly welcomed if a validated Italian version of the recent Neuro-QoL utility scoring system is available in the near future [40].

In conclusion, teriflunomide in RRMS naïve vs. experienced patients is cost-effective and possibly strongly dominant from both the healthcare sector and the society viewpoints in Italy.

However, even though decision models are well established in the health economic literature [3, 13, 14], our findings need further confirmation from real-world studies.

## Supplementary Information

Below is the link to the electronic supplementary material.

ESM 1 (DOCX 705 kb)

## Data Availability

Not applicable.
