# Supplementary material for: Cost-utility analysis of teriflunomide in naïve vs. previously treated patients with relapsing–remitting multiple sclerosis in Italy
Source: Neurol Sci. 2022 Apr 14;43(8):4933–44. doi: 10.1007/s10072-022-06022-x (PMC9349140; doi:10.1007/s10072-022-06022-x)
Supplement: Supplementary file 1 — (DOCX 705 kb) [file 10072_2022_6022_MOESM1_ESM.docx]

Cost-utility analysis of teriflunomide in naïve vs. previously treated patients with Relapsing-Remitting Multiple Sclerosis in Italy

Lazzaro C^1^, Bergamaschi R^2^, Zaffaroni M^3^, Totaro R^4^, Paolicelli D^5^

^1^ Studio di Economia Sanitaria, Milan, Italy

^2^ IRCCS Mondino Foundation, Pavia, Italy

^3^ Multiple Sclerosis Centre, Hospital of Gallarate, ASST della Valle Olona, Italy

^4^ Centro Malattie Demielinizzanti, Ospedale San Salvatore, L’Aquila, Italy

^5^ Department of Basic Medical Sciences, Neuroscience and Sense Organs, University of Bari "Aldo Moro", Italy

Corresponding author:

Carlo Lazzaro

Studio di Economia Sanitaria

Via Stefanardo da Vimercate, 19

Milan I -20128

Italy

landline/fax: + 39 02 2600.0516

E-mail address: carlo.lazzaro@tin.it

http://orcid.org/0000-0003-4795-1834

**ORCID**

Carlo Lazzaro http://orcid.org/0000-0003-4795-1834

Roberto Bergamaschi https://orcid.org/0000-0002-1397-511X

Mauro Zaffaroni <https://orcid.org/0000-0001-9020-934X>

Rocco Totaro https://orcid.org/0000-0001-5852-7743

Damiano Paolicelli <https://orcid.org/0000-0002-8645-1763>

# Supplementary Information (SI)Section A: SI Tables and Figures

| **List of abbreviations** | |
| --- | --- |
| ALT | Alanine Aminotransferase |
| AST | Aspartate Aminotransferase |
| CI | Confidence Interval |
| CV | Coefficient Of Variation |
| CUA | Cost-Utility Analysis |
| CVC | Central Venous Catheter |
| DH | Day-Hospital |
| DMT | Disease-Modifying Treatment |
| ECG | Electrocardiogram |
| EDSS | Expanded Disability Status Scale |
| gGT | Gamma-Glutamyl Transpeptidasis |
| HBV | Hepatitis B Virus |
| HCV | Hepatitis C Virus |
| HIV | Human Immunodeficiency Virus |
| HPV | Human Papilloma Virus |
| ICUR | Incremental Cost-Utility Ratio |
| im | intramuscular |
| INHS | Italian National Health Service |
| iv | intravenous |
| JCV | John Cunningham Virus |
| LL | Lower Limit |
| LYS | Life-Years Saved |
| MRI | Magnetic Resonance Imaging |
| OOP | Out-Of-Pocket |
| OTC | Optical Coherence Tomography |
| OWSA | One-Way Sensitivity Analysis |
| PSA | Probabilistic Sensitivity Analysis |
| QALYs | Quality-Adjusted Life Years |
| RRMS | Relapsing-Remitting Multiple Sclerosis |
| SE | Standard Error |
| SSA | Scenario Sensitivity Analysis |
| UL | Upper Limit |

**Table SI1 – Materials and Methods - Parameters with no statistical distribution for base case analysis, OWSA and PSA**

| **Parameter number** | **Parameter**  **name** | **Spreadsheet**  **(point estimate cell)** | **Parameter**  **description** | **Observations** | **Point estimate** | **LL**  **range** | **UL range** | **Source** |
| --- | --- | --- | --- | --- | --- | --- | --- | --- |
| **RRMS naïve and experienced patients on Teriflunomide** | | | | | | | | |
| **Anagraphics and demographics** | | | | | | | | |
| 1 | Age_naïve | Parameters (L20) | RRMS naïve patients’age when entering the model | 153 | 33 | 25 | 49 | Neurologists’opinion |
| 2 | Age_exp | Parameters (L21) | RRMS experienced patients’age when entering the model | 220 | 37 | 25 | 48 | Neurologists’opinion |
| **Clinical events – RRMS duration at Teriflunomide treatment** | | | | | | | | |
| 3 | RRMS_duration_naïve | Parameters (L23) | RRMS duration naïve patients when entering the model | 153 | 6 | 1 | 13 | Neurologists’opinion |
| 4 | RRMS_duration_exp | Parameters (L24) | RRMS duration edxperienced patients when entering the model | 220 | 11 | 8 | 13 | Neurologists’opinion |
| **Healthcare resources – Probability - Teriflunomide pre-treatment assessment** | | | | | | | | |
| 5 | Pr_pre_treatment_assessment_Teriflunomide | Parameters (L58) | Probability pre-treatment assessment Teriflunomide for both naïve and experienced RRMS patients | 373 | 1.000 | 1.000 | 1.000 | Neurologists’opinion |
| 6 | Pr_Blood_test_pre_Teriflunomide | Parameters (L59) | Probability full blood test for pre-treatment assessment Teriflunomide for both naïve and experienced RRMS patients | 373 | 1.000 | 1.000 | 1.000 | Neurologists’opinion |
| 7 | Pr_Haepatic_test_ALT_pre_Teriflunomide | Parameters (L66) | Probability ALT test for pre-treatment assessment Teriflunomide for both naïve and experienced RRMS patients | 373 | 1.000 | 1.000 | 1.000 | Neurologists’opinion |
| 8 | Pr_Haepatic_test_AST_pre_Teriflunomide | Parameters (L67) | Probability AST test for pre-treatment assessment Teriflunomide for both naïve and experienced RRMS patients | 373 | 1.000 | 1.000 | 1.000 | Neurologists’opinion |
| 9 | Pr_Haepatic_test_gGT_pre_Teriflunomide | Parameters (L68) | Probability gGT test for pre-treatment assessment Teriflunomide for both naïve and experienced RRMS patients | 373 | 1.000 | 1.000 | 1.000 | Neurologists’opinion |
| 10 | Pr_Sierology_test_quantiferon_pre_Teriflunomide | Parameters (L73) | Probability quantiferon test for pre-treatment assessment Teriflunomide for both naïve and experienced RRMS patients | 373 | 1.000 | 1.000 | 1.000 | Neurologists’opinion |
| 11 | Pr_Kidney_test_creatinine_pre_Teriflunomide | Parameters (L78) | Probability creatinine test for pre-treatment assessment Teriflunomide for both naïve and experienced RRMS patients | 373 | 1.000 | 1.000 | 1.000 | Neurologists’opinion |
| **Healthcare resources – Probability - Teriflunomide liver monitoring** | | | | | | | | |
| 12 | Pr_Haepatic_monitoring_Teriflunomide | Parameters (L82) | Probability liver monitoring during Teriflunomide treatment for both naïve and experienced RRMS patients | 373 | 1.000 | 1.000 | 1.000 | Neurologists’opinion |
| 13 | Pr_Haepatic_test_ALT_monitoring_1st_year_Teriflunomide | Parameters (L83) | Probability ALT test for monitoring during Teriflunomide treatment for both naïve and experienced RRMS patients 1^st^ year | 373 | 1.000 | 1.000 | 1.000 | Neurologists’opinion |
| 14 | Pr_Haepatic_test_ALT_monitoring_2_7_year_Teriflunomide | Parameters (L88) | Probability ALT test for monitoring during Teriflunomide treatment for both naïve and experienced RRMS patients year 2_7 | 373 | 1.000 | 1.000 | 1.000 | Neurologists’opinion |
| 15 | Pr_Haepatic_test_AST_monitoring_2_7_year_Teriflunomide | Parameters (L89) | Probability AST test for monitoring during Teriflunomide treatment for both naïve and experienced RRMS patients year 2_7 | 373 | 1.000 | 1.000 | 1.000 | Neurologists’opinion |

**Table SI2 – Materials and Methods - Parameters with no statistical distribution for base case analysis, OWSA and PSA**

| **Parameter number** | **Parameter**  **name** | **Spreadsheet**  **(point estimate cell)** | **Parameter**  **description** | **Observations** | **Point estimate** | **LL**  **range** | **UL**  **range** | **Source** |
| --- | --- | --- | --- | --- | --- | --- | --- | --- |
| **RRMS naïve and experienced patients on Teriflunomide** | | | | | | | | |
| **Healthcare resources – Probability - Teriflunomide liver monitoring** | | | | | | | | |
| 16 | Pr_Haepatic_test_gGT_monitoring_2_7_year_Teriflunomide | Parameters (L90) | Probability gGT test for monitoring during Teriflunomide treatment for both naïve and experienced RRMS patients year 2_7 | 373 | 1.000 | 1.000 | 1.000 | Neurologists’opinion |
| **Healthcare resources – Probability - RRMS no relapse - Follow-up** | | | | | | | | |
| 17 | Pr_fu_no_RRMS_relapse_Teriflunomide | Parameters (L94) | Probability follow-up with no relapse during Teriflunomide treatment for both naïve and experienced RRMS patients year 1_7 | 373 | 1.000 | 1.000 | 1.000 | Neurologists’opinion |
| 18 | Pr_Neurologist_visit_fu_RRMS_NO_relapse_naïve_1st_year_Teriflunomide | Parameters (L127) | Probability neurologist visit for follow-up with no relapse during Teriflunomide treatment for naïve RRMS patients 1^st^ year | 153 | 1.000 | 1.000 | 1.000 | Neurologists’opinion |
| 19 | Pr_Neurologist_visit_fu_RRMS_NO_relapse_exp_1st_year_Teriflunomide | Parameters (L127) | Probability neurologist visit for follow-up with no relapse during Teriflunomide treatment for experienced RRMS patients 1^st^ year | 220 | 1.000 | 1.000 | 1.000 | Neurologists’opinion |
| 20 | Pr_Neurologist_visit_fu_RRMS_NO_relapse_naïve_2_7_year_Teriflunomide | Parameters (L165) | Probability neurologist visit for follow-up with no relapse during Teriflunomide treatment for naïve RRMS patients year 2_7 | 153 | 1.000 | 1.000 | 1.000 | Neurologists’opinion |
| 21 | Pr_Neurologist_visit_fu_RRMS_NO_relapse_exp_2_7_year_Teriflunomide | Parameters (L166) | Probability neurologist visit for follow-up with no relapse during Teriflunomide treatment for experienced RRMS patients year 2_7 | 220 | 1.000 | 1.000 | 1.000 | Neurologists’opinion |
| **Healthcare resources – Probability - RRMS relapse - Management** | | | | | | | | |
| 22 | Cond_pr_relapse_management_given_RRMS_relapse_Teriflunomide | Parameters (L189) | Conditional probability management given RRMS relapse during Teriflunomide treatment for both naïve and experienced patients year 1_7 | 373 | 1.000 | 1.000 | 1.000 | Neurologists’opinion |
| 23 | Cond_pr_Neurologist_visit_given_RRMS_relapse_1st_year_Teriflunomide | Parameters (L201) | Conditional probability neurologist visit for management given RRMS relapse during Teriflunomide treatment for both naïve and experienced patients year 1_7 | 373 | 1.000 | 1.000 | 1.000 | Neurologists’opinion |
| 24 | Cond_pr_Neurologist_visit_given_RRMS_relapse_2_7_year_Teriflunomide | Parameters (L213) | Conditional probability neurologist visit for management given RRMS relapse during Teriflunomide treatment for both naïve and experienced patients year 2_7 | 373 | 1.000 | 1.000 | 1.000 | Neurologists’opinion |
| **Healthcare resources – Probability - RRMS relapse - Follow-up** | | | | | | | | |
| 25 | Cond_pr_fu_given_RRMS_relapse_Teriflunomide | Parameters (L215) | Conditional probability follow-up given RRMS relapse during Teriflunomide treatment for both naïve and experienced patients year 1_7 | 373 | 1.000 | 1.000 | 1.000 | Neurologists’opinion |
| **Switch to other DMTs due to Teriflunomide ineffectiveness in naïve patients - All DMTs** | | | | | | | | |
| **Healthcare resources – Probability - Pre-treatment assessment - Natalizumab** | | | | | | | | |
| 26 | Cond_pr_pre_Natalizumab_full_blood_count_given_fail_naïve_2_7_year_Teriflunomide | Parameters (L285) | Conditional probability full blood test pre-treatment assessment Natalizumab given poor response to Teriflunomide naïve patients years 2_7 | 15 | 1.000 | 1.000 | 1.000 | Neurologists’opinion |

**Table SI3 – Materials and Methods - Parameters with no statistical distribution for base case analysis, OWSA and PSA**

| **Parameter number** | **Parameter**  **name** | **Spreadsheet**  **(point estimate cell)** | **Parameter**  **description** | **Observations** | **Point estimate** | **LL range** | **UL range** | **Source** |
| --- | --- | --- | --- | --- | --- | --- | --- | --- |
| **RRMS naïve and experienced patients on Teriflunomide** | | | | | | | | |
| **Switch to other DMTs due to Teriflunomide ineffectiveness in naïve patients - All DMTs** | | | | | | | | |
| **Healthcare resources – Probability - Pre-treatment assessment - Natalizumab** | | | | | | | | |
| 27 | Cond_pr_pre_Natalizumab_haepatic_test_ALT_given_fail_naïve_2_7_year_Teriflunomide | Parameters (L286) | Conditional probability ALT test pre-treatment assessment Natalizumab given poor response to Teriflunomide naïve patients years 2_7 | 15 | 1.000 | 1.000 | 1.000 | Neurologists’opinion |
| 28 | Cond_pr_pre_Natalizumab_haepatic_test_AST_given_fail_naïve_2_7_year_Teriflunomide | Parameters (L287) | Conditional probability AST test pre-treatment assessment Natalizumab given poor response to Teriflunomide naïve patients years 2_7 | 15 | 1.000 | 1.000 | 1.000 | Neurologists’opinion |
| 29 | Cond_pr_pre_Natalizumab_haepatic_test_gGT_given_fail_naïve_2_7_year_Teriflunomide | Parameters (L288) | Conditional probability gGT test pre-treatment assessment Natalizumab given poor response to Teriflunomide naïve patients years 2_7 | 15 | 1.000 | 1.000 | 1.000 | Neurologists’opinion |
| 30 | Cond_pr_pre_Natalizumab_kidney_test_creatinine_given_fail_naïve_2_7_year_Teriflunomide | Parameters (L290) | Conditional probability creatinine test pre-treatment assessment Natalizumab given poor response to Teriflunomide naïve patients years 2_7 | 15 | 1.000 | 1.000 | 1.000 | Neurologists’opinion |
| 31 | Cond_pr_pre_Natalizumab_kidney_test_urea_given_fail_naïve_2_7_year_Teriflunomide | Parameters (L291) | Conditional probability urea test pre-treatment assessment Natalizumab given poor response to Teriflunomide naïve patients years 2_7 | 15 | 1.000 | 1.000 | 1.000 | Neurologists’opinion |
| 32 | Cond_pr_pre_Natalizumab_kidney_test_urine_given_fail_naïve_2_7_year_Teriflunomide | Parameters (L292) | Conditional probability urine test pre-treatment assessment Natalizumab given poor response to Teriflunomide naïve patients years 2_7 | 15 | 1.000 | 1.000 | 1.000 | Neurologists’opinion |
| 33 | Cond_pr_pre_Natalizumab_sierology_test_JCV_given_fail_naïve_2_7_year_Teriflunomide | Parameters (L293) | Conditional probability JCV test pre-treatment assessment Natalizumab given poor response to Teriflunomide naïve patients years 2_7 | 15 | 1.000 | 1.000 | 1.000 | Neurologists’opinion |

**Table SI4 – Materials and Methods - Parameters with no statistical distribution for base case analysis, OWSA and PSA**

| **Parameter number** | **Parameter**  **name** | **Spreadsheet**  **(point estimate cell)** | **Parameter**  **description** | **Observations** | **Point estimate** | **LL range** | **UL range** | **Source** |
| --- | --- | --- | --- | --- | --- | --- | --- | --- |
| **RRMS naïve and experienced patients on Teriflunomide** | | | | | | | | |
| **Switch to other DMTs due to Teriflunomide ineffectiveness in naïve patients - All DMTs** | | | | | | | | |
| **Healthcare resources – Probability - During treatment assessment - Natalizumab** | | | | | | | | |
| 34 | Cond_pr_during_Natalizumab_full_blood_count_given_fail_naïve_2_7_year_Teriflunomide | Parameters (L295) | Conditional probability full blood test during Natalizumab treatment given poor response to Teriflunomide naïve patients years 2_7 | 15 | 1.000 | 1.000 | 1.000 | Neurologists’opinion |
| 35 | Cond_pr_during_Natalizumab_haepatic_test_ALT_given_fail_naïve_2_7_year_Teriflunomide | Parameters (L296) | Conditional probability ALT test during Natalizumab treatment given poor response to Teriflunomide naïve patients years 2_7 | 15 | 1.000 | 1.000 | 1.000 | Neurologists’opinion |
| 36 | Cond_pr_during_Natalizumab_haepatic_test_AST_given_fail_naïve_2_7_year_Teriflunomide | Parameters (L297) | Conditional probability AST test during Natalizumab treatment given poor response to Teriflunomide naïve patients years 2_7 | 15 | 1.000 | 1.000 | 1.000 | Neurologists’opinion |
| 37 | Cond_pr_during_Natalizumab_haepatic_test_gGT_given_fail_naïve_2_7_year_Teriflunomide | Parameters (L298) | Conditional probability gGT test during Natalizumab treatment given poor response to Teriflunomide naïve patients years 2_7 | 15 | 1.000 | 1.000 | 1.000 | Neurologists’opinion |
| 38 | Cond_pr_during_Natalizumab_kidney_test_creatinine_given_fail_naïve_2_7_year_Teriflunomide | Parameters (L300) | Conditional probability kidney test during Natalizumab treatment given poor response to Teriflunomide naïve patients years 2_7 | 15 | 1.000 | 1.000 | 1.000 | Neurologists’opinion |
| 39 | Cond_pr_during_Natalizumab_kidney_test_urea_given_fail_naïve_2_7_year_Teriflunomide | Parameters (L301) | Conditional probability urea test during Natalizumab treatment given poor response to Teriflunomide naïve patients years 2_7 | 15 | 1.000 | 1.000 | 1.000 | Neurologists’opinion |
| 40 | Cond_pr_during_Natalizumab_kidney_test_urine_given_fail_naïve_2_7_year_Teriflunomide | Parameters (L302) | Conditional probability urine test during Natalizumab treatment given poor response to Teriflunomide naïve patients years 2_7 | 15 | 1.000 | 1.000 | 1.000 | Neurologists’opinion |
| **Healthcare resources – Probability - Administration disposable - Natalizumab** | | | | | | | | |
| 41 | Cond_pr_adm_physio_Natalizumab_given_fail_naïve_2_7_year_Teriflunomide | Parameters (L313) | Conditional probability physiological solution administration Natalizumab given poor response to Teriflunomide naïve patients years 2_7 | 15 | 1.000 | 1.000 | 1.000 | Neurologists’opinion |
| 42 | Cond_pr_adm_iv_Natalizumab_given_fail_naïve_2_7_year_Teriflunomide | Parameters (L314) | Conditional probability iv line administration Natalizumab given poor response to Teriflunomide naïve patients years 2_7 | 15 | 1.000 | 1.000 | 1.000 | Neurologists’opinion |

**Table SI5 – Materials and Methods - Parameters with no statistical distribution for base case analysis, OWSA and PSA**

| **Parameter number** | **Parameter**  **name** | **Spreadsheet**  **(point estimate cell)** | **Parameter**  **description** | **Observations** | **Point estimate** | **LL range** | **UL range** | **Source** |
| --- | --- | --- | --- | --- | --- | --- | --- | --- |
| **RRMS naïve and experienced patients on Teriflunomide** | | | | | | | | |
| **Switch to other DMTs due to Teriflunomide ineffectiveness in naïve patients - All DMTs** | | | | | | | | |
| **Healthcare resources – Probability - Administration disposable - Natalizumab** | | | | | | | | |
| 43 | Cond_pr_adm_needle_Natalizumab_given_fail_naïve_2_7_year_Teriflunomide | Parameters (L315) | Conditional probability needle administration Natalizumab given poor response to Teriflunomide naïve patients years 2_7 | 15 | 1.000 | 1.000 | 1.000 | Neurologists’opinion |
| 44 | Cond_pr_adm_antiseptic_Natalizumab_given_fail_naïve_2_7_year_Teriflunomide | Parameters (L316) | Conditional probability antiseptic administration Natalizumab given poor response to Teriflunomide naïve patients years 2_7 | 15 | 1.000 | 1.000 | 1.000 | Neurologists’opinion |
| **Healthcare resources – Probability - Postadministration time - Natalizumab** | | | | | | | | |
| 45 | Cond_pr_postad_time_nurse_Natalizumab_given_fail_naïve_2_7_year_Teriflunomide | Parameters (L322) | Conditional probability nurse time for administration Natalizumab given poor response to Teriflunomide naïve patients years 2_7 | 15 | 1.000 | 1.000 | 1.000 | Neurologists’opinion |
| **Healthcare resources – Probability - Pre-treatment assessment - Fingolimod** | | | | | | | | |
| 46 | Cond_pr_pre_Fingolimod_ECG_given_fail_naïve_2_7_year_Teriflunomide | Parameters (L325) | Conditional probability ECG test for pre-treatment assessment Fingolimod given poor response to Teriflunomide naïve patients years 2_7 | 11 | 1.000 | 1.000 | 1.000 | Neurologists’opinion |
| 47 | Cond_pr_pre_Fingolimod_OCT_given_fail_naïve_2_7_year_Teriflunomide | Parameters (L326) | Conditional probability OCT test for pre-treatment assessment Fingolimod given poor response to Teriflunomide naïve patients years 2_7 | 11 | 1.000 | 1.000 | 1.000 | Neurologists’opinion |
| 48 | Cond_pr_pre_Fingolimod_sierology_test_zooster_given_fail_naïve_2_7_year_Teriflunomide | Parameters (L327) | Conditional probability zooster test for pre-treatment assessment Fingolimod given poor response to Teriflunomide naïve patients years 2_7 | 11 | 1.000 | 1.000 | 1.000 | Neurologists’opinion |
| 49 | Cond_pr_pre_Fingolimod_dermatologist_visit_given_fail_naïve_2_7_year_Teriflunomide | Parameters (L328) | Conditional probability dermatologist visit for pre-treatment assessment Fingolimod given poor response to Teriflunomide naïve patients years 2_7 | 11 | 1.000 | 1.000 | 1.000 | Neurologists’opinion |
| **Healthcare resources – Probability - Administration setting - Fingolimod** | | | | | | | | |
| 50 | Cond_pr_adm_DH_1st_adm_only_Fingolimod_given_fail_naïve_2_7_year_Teriflunomide | Parameters (L330) | Conditional probability 1^st^ administration in DH setting Fingolimod given poor response to Teriflunomide naïve patients years 2_7 | 11 | 0.003 | 0.003 | 0.003 | Neurologists’opinion |
| **Healthcare resources – Probability - Pre-treatment assessment - Cladribine** | | | | | | | | |
| 51 | Cond_pr_pre_ Cladribine_haepatic_test_ALT_given_fail_naïve_2_7_year_Teriflunomide | Parameters (L333) | Conditional probability ALT test pre-treatment assessment Cladribine given poor response to Teriflunomide naïve patients years 2_7 | 7 | 1.000 | 1.000 | 1.000 | Neurologists’opinion |
| 52 | Cond_pr_pre_ Cladribine_haepatic_test_AST_given_fail_naïve_2_7_year_Teriflunomide | Parameters (L334) | Conditional probability AST test pre-treatment assessment Cladribine given poor response to Teriflunomide naïve patients years 2_7 | 7 | 1.000 | 1.000 | 1.000 | Neurologists’opinion |

**Table SI6 – Materials and Methods - Parameters with no statistical distribution for base case analysis, OWSA and PSA**

| **Parameter number** | **Parameter**  **name** | **Spreadsheet**  **(point estimate cell)** | **Parameter**  **description** | **Observations** | **Point estimate** | **LL range** | **UL range** | **Source** |
| --- | --- | --- | --- | --- | --- | --- | --- | --- |
| **RRMS naïve and experienced patients on Teriflunomide** | | | | | | | | |
| **Switch to other DMTs due to Teriflunomide ineffectiveness in naïve patients - All DMTs** | | | | | | | | |
| **Healthcare resources – Probability - Pre-treatment assessment - Cladribine** | | | | | | | | |
| 53 | Cond_pr_pre_ Cladribine_haepatic_test_gGT_given_fail_naïve_2_7_year_Teriflunomide | Parameters (L335) | Conditional probability gGT test pre-treatment assessment Cladribine given poor response to Teriflunomide naïve patients years 2_7 | 7 | 1.000 | 1.000 | 1.000 | Neurologists’opinion |
| 54 | Cond_pr_pre_Cladribine_kidney_test_creatinine_given_fail_naïve_2_7_year_Teriflunomide | Parameters (L336) | Conditional probability creatinine test pre-treatment assessment Cladribine given poor response to Teriflunomide naïve patients years 2_7 | 7 | 1.000 | 1.000 | 1.000 | Neurologists’opinion |
| 55 | Cond_pr_pre_Cladribine_kidney_test_urea_given_fail_naïve_2_7_year_Teriflunomide | Parameters (L337) | Conditional probability urea test pre-treatment assessment Cladribine given poor response to Teriflunomide naïve patients years 2_7 | 7 | 1.000 | 1.000 | 1.000 | Neurologists’opinion |
| 56 | Cond_pr_pre_Cladribine_kidney_test_urine_given_fail_naïve_2_7_year_Teriflunomide | Parameters (L338) | Conditional probability urine test pre-treatment assessment Cladribine given poor response to Teriflunomide naïve patients years 2_7 | 7 | 1.000 | 1.000 | 1.000 | Neurologists’opinion |
| 57 | Cond_pr_pre_Cladribine_sierology_test_HBV_given_fail_naïve_2_7_year_Teriflunomide | Parameters (L339) | Conditional probability HBV test pre-treatment assessment Cladribine given poor response to Teriflunomide naïve patients years 2_7 | 7 | 1.000 | 1.000 | 1.000 | Neurologists’opinion |
| 58 | Cond_pr_pre_Cladribine_sierology_test_HCV_given_fail_naïve_2_7_year_Teriflunomide | Parameters (L340) | Conditional probability HCV test pre-treatment assessment Cladribine given poor response to Teriflunomide naïve patients years 2_7 | 7 | 1.000 | 1.000 | 1.000 | Neurologists’opinion |
| 59 | Cond_pr_pre_Cladribine_sierology_test_zooster_given_fail_naïve_2_7_year_Teriflunomide | Parameters (L341) | Conditional probability zooster test pre-treatment assessment Cladribine given poor response to Teriflunomide naïve patients years 2_7 | 7 | 1.000 | 1.000 | 1.000 | Neurologists’opinion |
| 60 | Cond_pr_pre_Cladribine_sierology_test_quantiferon_given_fail_naïve_2_7_year_Teriflunomide | Parameters (L342) | Conditional probability quantiferon test pre-treatment assessment Cladribine given poor response to Teriflunomide naïve patients years 2_7 | 7 | 1.000 | 1.000 | 1.000 | Neurologists’opinion |
| **Healthcare resources – Probability - Premedication - Ocrelizumab** | | | | | | | | |
| 61 | Cond_pr_premed_corticosteroids_Ocrelizumab_given_fail_naïve_2_7_year_Teriflunomide | Parameters (L345) | Conditional probability corticosteroids premedication Ocrelizumab given poor response to Teriflunomide naïve patients years 2_7 | 6 | 1.000 | 1.000 | 1.000 | Neurologists’opinion |

**Table SI7 – Materials and Methods - Parameters with no statistical distribution for base case analysis, OWSA and PSA**

| **Parameter number** | **Parameter**  **name** | **Spreadsheet**  **(point estimate cell)** | **Parameter**  **description** | **Observations** | **Point estimate** | **LL range** | **UL range** | **Source** |
| --- | --- | --- | --- | --- | --- | --- | --- | --- |
| **RRMS naïve and experienced patients on Teriflunomide** | | | | | | | | |
| **Switch to other DMTs due to Teriflunomide ineffectiveness in naïve patients - All DMTs** | | | | | | | | |
| **Healthcare resources – Probability - Premedication - Ocrelizumab** | | | | | | | | |
| 62 | Cond_pr_premed_antihistaminics_Ocrelizumab_given_fail_naïve_2_7_year_Teriflunomide | Parameters (L346) | Conditional probability antihistaminics premedication Ocrelizumab given poor response to Teriflunomide naïve patients years 2_7 | 6 | 1.000 | 1.000 | 1.000 | Neurologists’opinion |
| 63 | Cond_pr_premed_antihistaminics_Ocrelizumab_given_fail_naïve_2_7_year_Teriflunomide | Parameters (L347) | Conditional probability antihistaminics premedication Ocrelizumab given poor response to Teriflunomide naïve patients years 2_7 | 6 | 1.000 | 1.000 | 1.000 | Neurologists’opinion |
| **Healthcare resources – Probability - Administration setting - Ocrelizumab** | | | | | | | | |
| 64 | Cond_pr_adm_DH_Ocrelizumab_given_fail_naïve_2_7_year_Teriflunomide | Parameters (L349) | Conditional probabilityadministration in DH setting Fingolimod given poor response to Teriflunomide naïve patients years 2_7 | 6 | 1.000 | 1.000 | 1.000 | Neurologists’opinion |
| **Healthcare resources – Probability - Administration time - Ocrelizumab** | | | | | | | | |
| 65 | Cond_pr_adm_time_nurse_Ocrelizumab_given_fail_naïve_2_7_year_Teriflunomide | Parameters (L351) | Conditional probability nurse time for administration Ocrelizumab given poor response to Teriflunomide naïve patients years 2_7 | 6 | 1.000 | 1.000 | 1.000 | Neurologists’opinion |
| **Healthcare resources – Probability - Administration disposable - Ocrelizumab** | | | | | | | | |
| 66 | Cond_pr_adm_iv_line_Ocrelizumab_given_fail_naïve_2_7_year_Teriflunomide | Parameters (L353) | Conditional probability iv line for administration Ocrelizumab given poor response to Teriflunomide naïve patients years 2_7 | 6 | 1.000 | 1.000 | 1.000 | Neurologists’opinion |
| 67 | Cond_pr_adm_needle_Ocrelizumab_given_fail_naïve_2_7_year_Teriflunomide | Parameters (L354) | Conditional probability needle for administration Ocrelizumab given poor response to Teriflunomide naïve patients years 2_7 | 6 | 1.000 | 1.000 | 1.000 | Neurologists’opinion |
| 68 | Cond_pr_adm_antiseptic_Ocrelizumab_given_fail_naïve_2_7_year_Teriflunomide | Parameters (L355) | Conditional probability antiseptic for administration Ocrelizumab given poor response to Teriflunomide naïve patients years 2_7 | 6 | 1.000 | 1.000 | 1.000 | Neurologists’opinion |
| **Healthcare resources – Probability - Postadministration disposable - Ocrelizumab** | | | | | | | | |
| 69 | Cond_pr_postad_physio_Ocrelizumab_given_fail_naïve_2_7_year_Teriflunomide | Parameters (L357) | Conditional probability physiological solution for postadministration Ocrelizumab given poor response to Teriflunomide naïve patients years 2_7 | 6 | 1.000 | 1.000 | 1.000 | Neurologists’opinion |
| **Healthcare resources – Probability - Pre-treatment assessment - Alemtuzumab** | | | | | | | | |
| 70 | Cond_pr_pre_Alemtuzumab_haepatic_test_ALT_given_fail_naïve_2_7_year_Teriflunomide | Parameters (L362) | Conditional probability ALT test for pre-treatment assessment Alemtuzumab given poor response to Teriflunomide naïve patients years 2_7 | 5 | 1.000 | 1.000 | 1.000 | Neurologists’opinion |
| 71 | Cond_pr_pre_Alemtuzumab_haepatic_test_AST_given_fail_naïve_2_7_year_Teriflunomide | Parameters (L363) | Conditional probability AST test for pre-treatment assessment Alemtuzumab given poor response to Teriflunomide naïve patients years 2_7 | 5 | 1.000 | 1.000 | 1.000 | Neurologists’opinion |
| 72 | Cond_pr_pre_Alemtuzumab_haepatic_test_gGT_given_fail_naïve_2_7_year_Teriflunomide | Parameters (L364) | Conditional probability gGT test test for pre-treatment assessment Alemtuzumab given poor response to Teriflunomide naïve patients years 2_7 | 5 | 1.000 | 1.000 | 1.000 | Neurologists’opinion |

**Table SI8 – Materials and Methods - Parameters with no statistical distribution for base case analysis, OWSA and PSA**

| **Parameter number** | **Parameter**  **name** | **Spreadsheet**  **(point estimate cell)** | **Parameter**  **description** | **Observations** | **Point estimate** | **LL range** | **UL range** | **Source** |
| --- | --- | --- | --- | --- | --- | --- | --- | --- |
| **RRMS naïve and experienced patients on Teriflunomide** | | | | | | | | |
| **Switch to other DMTs due to Teriflunomide ineffectiveness in naïve patients - All DMTs** | | | | | | | | |
| **Healthcare resources – Probability - Pre-treatment assessment - Alemtuzumab** | | | | | | | | |
| 73 | Cond_pr_pre_Alemtuzumab_sierology_test_HBV_given_fail_naïve_2_7_year_Teriflunomide | Parameters (L365) | Conditional probability HBV test for pre-treatment assessment Alemtuzumab given poor response to Teriflunomide naïve patients years 2_7 | 5 | 1.000 | 1.000 | 1.000 | Neurologists’opinion |
| 74 | Cond_pr_pre_Alemtuzumab_sierology_test_HCV_given_fail_naïve_2_7_year_Teriflunomide | Parameters (L366) | Conditional probability HCV test for pre-treatment assessment Alemtuzumab given poor response to Teriflunomide naïve patients years 2_7 | 5 | 1.000 | 1.000 | 1.000 | Neurologists’opinion |
| **Healthcare resources – Probability – Premedication - Alemtuzumab** | | | | | | | | |
| 75 | Cond_pr_premed_corticosteroids_Alemtuzumab _given_fail_naïve_2_7_year_Teriflunomide | Parameters (L369) | Conditional probability corticosteroids premedication Alemtuzumab given poor response to Teriflunomide naïve patients years 2_7 | 5 | 1.000 | 1.000 | 1.000 | Neurologists’opinion |
| 76 | Cond_pr_premed_antihistaminics_Alemtuzumab _given_fail_naïve_2_7_year_Teriflunomide | Parameters (L370) | Conditional probability antihistaminics premedication Alemtuzumab given poor response to Teriflunomide naïve patients years 2_7 | 5 | 1.000 | 1.000 | 1.000 | Neurologists’opinion |
| 77 | Cond_pr_premed_time_nurse_Alemtuzumab_given_fail_naïve_2_7_year_Teriflunomide | Parameters (L371) | Conditional probability nurse time premedication Alemtuzumab given poor response to Teriflunomide naïve patients years 2_7 | 5 | 1.000 | 1.000 | 1.000 | Neurologists’opinion |
| **Healthcare resources – Probability – Administration setting - Alemtuzumab** | | | | | | | | |
|  |  |  |  |  |  |  |  |  |
| 78 | Cond_pr_adm_DH_Alemtuzumab_given_fail_naïve_2_7_year_Teriflunomide | Parameters (L373) | Conditional probability DH administration Alemtuzumab given poor response to Teriflunomide naïve patients years 2_7 | 5 | 1.000 | 1.000 | 1.000 | Neurologists’opinion |
| **Healthcare resources – Probability – Administration disposables - Alemtuzumab** | | | | | | | | |
| 79 | Cond_pr_adm_iv_line_Alemtuzumab_given_fail_naïve_2_7_year_Teriflunomide | Parameters (L375) | Conditional probability iv line administration Alemtuzumab given poor response to Teriflunomide naïve patients years 2_7 | 5 | 1.000 | 1.000 | 1.000 | Neurologists’opinion |
| 80 | Cond_pr_adm_needle_Alemtuzumab_given_fail_naïve_2_7_year_Teriflunomide | Parameters (L376) | Conditional probability needle administration Alemtuzumab given poor response to Teriflunomide naïve patients years 2_77 | 5 | 1.000 | 1.000 | 1.000 | Neurologists’opinion |
|  |  |  |  |  |  |  |  |  |
| 81 | Cond_pr_adm_antiseptic_Alemtuzumab_given_fail_naïve_2_7_year_Teriflunomide | Parameters (L377) | Conditional probability antiseptic administration Alemtuzumab given poor response to Teriflunomide naïve patients years 2_7 | 5 | 1.000 | 1.000 | 1.000 | Neurologists’opinion |

**Table SI9 – Materials and Methods - Parameters with no statistical distribution for base case analysis, OWSA and PSA**

| **Parameter number** | **Parameter**  **name** | **Spreadsheet**  **(point estimate cell)** | **Parameter**  **description** | **Observations** | **Point estimate** | **LL range** | **UL range** | **Source** |
| --- | --- | --- | --- | --- | --- | --- | --- | --- |
| **RRMS naïve and experienced patients on Teriflunomide** | | | | | | | | |
| **Switch to other DMTs due to Teriflunomide ineffectiveness in naïve patients - All DMTs** | | | | | | | | |
| **Healthcare resources – Probability – Postadministration disposanbles - Alemtuzumab** | | | | | | | | |
| 82 | Cond_pr_postad_physio_Alemtuzumab_given_fail_naïve_2_7_year_Teriflunomide | Parameters (L379) | Conditional probability physiological solution administration Alemtuzumab given poor response to Teriflunomide naïve patients years 2_7 | 5 | 1.000 | 1.000 | 1.000 | Neurologists’opinion |
| **Non-healthcare resources – Probability – Transportation** | | | | | | | | |
| 83 | Pr_Car_Transportation | Parameters (L383) | Probability transportation by car during Teriflunomide treatment for both naïve and experienced RRMS patients year 1_7 | 5 | 1.000 | 1.000 | 1.000 | Research assumption |
| **Other probailities** | | | | | | | | |
| 84 | Half_cycle_corr | Parameters (L385) | Probability death occurring half-way between each Markov cycle for both naïve and experienced RRMS patients year 1_7 | 5 | 0.500 | 0.500 | 0.500 | [SI1.]; [SI2.] |
| **Healthcare resources - Volume** | | | | | | | | |
| **Posology - Teriflunomide** | | | | | | | | |
| 85 | Mg_adm_Teriflunomide | Parameters (L388) | Teriflunomide posology (mg) *per* administration for both naïve and experienced RRMS patients year 1_7 | 373 | 14 | 14 | 14 | [SI3.] |
| 86 | Vol_adm_Teriflunomide | Parameters (L389) | Number of administrations *per* year Teriflunomide for both naïve and experienced RRMS patients year 1_7 | 373 | 365.25 | 365.25 | 365.25 | [SI3.] |
| **Pre-treatment assessment - Teriflunomide** | | | | | | | | |
| 87 | Vol_Blood_test_pre_Teriflunoide | Parameters (L391) | Number of full blood tests for pre-treatment assessment Teriflunomide for both naïve and experienced RRMS patients | 373 | 1.000 | 1.000 | 1.000 | Neurologists’opinion |
| 88 | Vol_Lympho_subpop_test_pre_Teriflunomide | Parameters (L392) | Number of lymphocyte subpopulation tests for pre-treatment assessment Teriflunomide for both naïve and experienced RRMS patients | 373 | 1.000 | 1.000 | 1.000 | Neurologists’opinion |
| 89 | Vol_Neurologist_visit_pre_Teriflunomide | Parameters (L393) | Number of neurologist visits for pre-treatment assessment Teriflunomide for both naïve and experienced RRMS patients | 373 | 1.000 | 1.000 | 1.000 | Neurologists’opinion |
| 90 | Vol_Dermatologist_visit_pre_Teriflunomide | Parameters (L394) | Number of dermatologist visits for pre-treatment assessment Teriflunomide for both naïve and experienced RRMS patients | 373 | 1.000 | 1.000 | 1.000 | Neurologists’opinion |
| 91 | Vol_Cardiologist_visit_pre_Teriflunomide | Parameters (L395) | Number of cardiologist visit sfor pre-treatment assessment Teriflunomide for both naïve and experienced RRMS patients | 373 | 1.000 | 1.000 | 1.000 | Neurologists’opinion |
| 92 | Vol_ECG_test_pre_Teriflunomide | Parameters (L396) | Number of ECG tests for pre-treatment assessment Teriflunomide for both naïve and experienced RRMS patients | 373 | 1.000 | 1.000 | 1.000 | Neurologists’opinion |
| 93 | Vol_Brain_MRI _pre_Teriflunomide | Parameters (L397) | Number of brain MRIs for pre-treatment assessment Teriflunomide for both naïve and experienced RRMS patients | 373 | 1.000 | 1.000 | 1.000 | Neurologists’opinion |
| 94 | Vol_Haepatic_test_ALT_pre_Teriflunomide | Parameters (L398) | Number of ALT tests for pre-treatment assessment Teriflunomide for both naïve and experienced RRMS patients | 373 | 1.000 | 1.000 | 1.000 | Neurologists’opinion |
| 95 | Vol_Haepatic_test_AST_pre_Teriflunomide | Parameters (L399) | Number of AST tests for pre-treatment assessment Teriflunomide for both naïve and experienced RRMS patients | 373 | 1.000 | 1.000 | 1.000 | Neurologists’opinion |

**Table SI10 – Materials and Methods - Parameters with no statistical distribution for base case analysis, OWSA and PSA**

| **Parameter number** | **Parameter**  **name** | **Spreadsheet**  **(point estimate cell)** | **Parameter**  **description** | **Observations** | **Point estimate** | **LL range** | **UL range** | **Source** |
| --- | --- | --- | --- | --- | --- | --- | --- | --- |
| **RRMS naïve and experienced patients on Teriflunomide** | | | | | | | | |
| **Healthcare resources - Volume** | | | | | | | | |
| **Pre-treatment assessment - Teriflunomide** | | | | | | | | |
| 96 | Vol_Haepatic_test_gGT_pre_Teriflunomide | Parameters (L400) | Number of gGT tests for pre-treatment assessment Teriflunomide for both naïve and experienced RRMS patients | 373 | 1.000 | 1.000 | 1.000 | Neurologists’opinion |
| 97 | Vol_Haepatic_test_Total_bilirubin_pre_Teriflunomide | Parameters (L401) | Number of total bilirubin tests for pre-treatment assessment Teriflunomide for both naïve and experienced RRMS patients | 373 | 1.000 | 1.000 | 1.000 | Neurologists’opinion |
| 98 | Vol_Haepatic_test_Direct_bilirubin_pre_Teriflunomide | Parameters (L402) | Number of direct bilirubin tests for pre-treatment assessment Teriflunomide for both naïve and experienced RRMS patients | 373 | 1.000 | 1.000 | 1.000 | Neurologists’opinion |
| 99 | Vol_Haepatic_test_Indirect_bilirubin_pre_Teriflunomide | Parameters (L403) | Number of indirect bilirubin tests for pre-treatment assessment Teriflunomide for both naïve and experienced RRMS patients | 373 | 1.000 | 1.000 | 1.000 | Neurologists’opinion |
| 100 | Vol_Haepatic_test_Alkaline_phosphatase_pre_Teriflunomide | Parameters (L404) | Number of alkaline phosphatase tests for pre-treatment assessment Teriflunomide for both naïve and experienced RRMS patients | 373 | 1.000 | 1.000 | 1.000 | Neurologists’opinion |
| 101 | Vol_Sierology_test_quantiferon_pre_Teriflunomide | Parameters (L405) | Number of quantiferon tests for pre-treatment assessment Teriflunomide for both naïve and experienced RRMS patients | 373 | 1.000 | 1.000 | 1.000 | Neurologists’opinion |
| 102 | Vol_Sierology_test_HBV_pre_Teriflunomide | Parameters (L406) | Number of HBV tests for pre-treatment assessment Teriflunomide for both naïve and experienced RRMS patients | 373 | 1.000 | 1.000 | 1.000 | Neurologists’opinion |
| 103 | Vol_Sierology_test_HCV_pre_Teriflunomide | Parameters (L407) | Number of HCV tests for pre-treatment assessment Teriflunomide for both naïve and experienced RRMS patients | 373 | 1.000 | 1.000 | 1.000 | Neurologists’opinion |
| 104 | Vol_Sierology_test_HIV_pre_Teriflunomide | Parameters (L408) | Number of HIV tests for pre-treatment assessment Teriflunomide for both naïve and experienced RRMS patients | 373 | 1.000 | 1.000 | 1.000 | Neurologists’opinion |
| 105 | Vol_Sierology_test_zooster_pre_Teriflunomide | Parameters (L409) | Number of zoster tests for pre-treatment assessment Teriflunomide for both naïve and experienced RRMS patients | 373 | 1.000 | 1.000 | 1.000 | Neurologists’opinion |
| 106 | Vol_Kidney_test_Creatinine_pre_Teriflunomide | Parameters (L410) | Number of creatinine tests for pre-treatment assessment Teriflunomide for both naïve and experienced RRMS patients | 373 | 1.000 | 1.000 | 1.000 | Neurologists’opinion |
| 107 | Vol_Kidney_test_Urea_pre_Teriflunomide | Parameters (L411) | Number of urea tests for pre-treatment assessment Teriflunomide for both naïve and experienced RRMS patients | 373 | 1.000 | 1.000 | 1.000 | Neurologists’opinion |
| 108 | Vol_Kidney_test_Urine_pre_Teriflunomide | Parameters (L412) | Number of urine tests for pre-treatment assessment Teriflunomide for both naïve and experienced RRMS patients | 373 | 1.000 | 1.000 | 1.000 | Neurologists’opinion |
| **RRMS no relapse - Follow-up** | | | | | | | | |
| 109 | Vol_Dermatologist_visit_fu_RRMS_NO_relapse_naïve_1st_year_Teriflunomide | Parameters (L459) | Number of dermatologist visits for follow-up with no relapse during Teriflunomide treatment for naïve RRMS patients 1st year | 153 | 1.000 | 1.000 | 1.000 | Neurologists’opinion |
| 110 | Vol_Dermatologist_visit_fu_RRMS_NO_relapse_exp_1st_year_Teriflunomide | Parameters (L460) | Number of dermatologist visits for follow-up with no relapse during Teriflunomide treatment for experienced RRMS patients 1st year | 220 | 1.000 | 1.000 | 1.000 | Neurologists’opinion |
| 111 | Vol_ Cardiologist _visit_fu_RRMS_NO_relapse_exp_1st_year_Teriflunomide | Parameters (L461) | Number of cardiologist visits for follow-up with no relapse during Teriflunomide treatment for experienced RRMS patients 1st year | 220 | 1.000 | 1.000 | 1.000 | Neurologists’opinion |
| 112 | Vol_Cardiologist_visit_fu_RRMS_NO_relapse_exp_1st_year_Teriflunomide | Parameters (L462) | Number of cardiologist visits for follow-up with no relapse during Teriflunomide treatment for experienced RRMS patients 1st year | 153 | 2.000 | 2.000 | 2.000 | Neurologists’opinion |

**Table SM11 – Materials and Methods - Parameters with no statistical distribution for base case analysis, OWSA and PSA**

| **Parameter number** | **Parameter**  **name** | **Spreadsheet**  **(point estimate cell)** | **Parameter**  **description** | **Observations** | **Point estimate** | **LL range** | **UL**  **range** | **Source** |
| --- | --- | --- | --- | --- | --- | --- | --- | --- |
| **RRMS naïve and experienced patients on Teriflunomide** | | | | | | | | |
| **Healthcare resources - Volume** | | | | | | | | |
| **RRMS no relapse - Follow-up** | | | | | | | | |
| 113 | Vol_Brain_MRI_plus_spinal_cord_C_fu_RRMS_2_7_year_naïve_Teriflunomide | Parameters (L467) | Number of brain plus spinal cord MRIs with contrast for naïve RRMS patients for follow-up with no relapse during Teriflunomide treatment years 2-7 | 153 | 1.000 | 1.000 | 1.000 | Neurologists’opinion |
| 114 | Vol_Brain_MRI_plus_spinal_cord_C_fu_RRMS_2_7_year_exp_Teriflunomide | Parameters (L468) | Number of brain plus spinal cord MRIs with contrast for experienced RRMS patients for follow-up with no relapse during Teriflunomide treatment years 2-7 | 220 | 1.000 | 1.000 | 1.000 | Neurologists’opinion |
| 115 | Vol_Dermatologist_visit_fu_RRMS_NO_relapse_naïve_2_7_year_Teriflunomide | Parameters (L497) | Number of dermatologist visits for naïve RRMS patients for follow-up with no relapse during Teriflunomide treatment years 2-7 | 153 | 1.000 | 1.000 | 1.000 | Neurologists’opinion |
| 116 | Vol_Dermatologist_visit_fu_RRMS_NO_relapse_exp_2_7_year_Teriflunomide | Parameters (L498) | Number of dermatologist visits for experienced RRMS patients for follow-up with no relapse during Teriflunomide treatment years 2-7 | 220 | 1.000 | 1.000 | 1.000 | Neurologists’opinion |
| 117 | Vol_Cardiologist_visit_fu_RRMS_NO_relapse_naïve_2_7_year_Teriflunomide | Parameters (L499) | Number of cardiologist visits for naïve RRMS patients for follow-up with no relapse during Teriflunomide treatment years 2-7 | 153 | 1.000 | 1.000 | 1.000 | Neurologists’opinion |
| 118 | Vol_Cardiologis_visit_fu_RRMS_NO_relapse_exp_2_7_year_Teriflunomide | Parameters (L500) | Number of cardiologist visits for experienced RRMS patients for follow-up with no relapse during Teriflunomide treatment years 2-7 | 220 | 1.000 | 1.000 | 1.000 | Neurologists’opinion |
| **RRMS relapse - Management** | | | | | | | | |
| 119 | Posology_corticosteroids_given_RRMS_relapse_1st_year_Teriflunomide | Parameters (L502) | Posology corticosteroids (grams *per diem*) given RRMS relapse for both naïve and experienced patients 1st year | 373 | 0.950 | 0.950 | 0.950 | Neurologists’opinion |
| 120 | Duration_corticosteroids_given_RRMS_relapse_1st_year_Teriflunomide | Parameters (L503) | Threrapy duration (days) given RRMS relapse for both naïve and experienced patients 1st year | 373 | 4.750 | 4.750 | 4.750 | Neurologists’opinion |
| 121 | Vol_Blood_test_given_RRMS_relapse_1st_year | Parameters (L504) | Number of full blood tests given RRMS relapse for both naïve and experienced patients 1st year | 373 | 1.000 | 1.000 | 1.000 | Neurologists’opinion |
| 122 | Vol_metabolic_test_blood_sugar_given_RRMS_relapse_1st_year | Parameters (L505) | Number of blood sugar tests given RRMS relapse for both naïve and experienced patients 1st year | 373 | 1.000 | 1.000 | 1.000 | Neurologists’opinion |
| 123 | Vol_Kidney_test_Creatinine_given_RRMS_relapse_1st_year | Parameters (L506) | Number of creatinine tests given RRMS relapse for both naïve and experienced patients 1st year | 373 | 1.000 | 1.000 | 1.000 | Neurologists’opinion |
| 124 | Vol_Kidney_test_Urine_given_RRMS_relapse_1st_year | Parameters (L507) | Number of urine tests given RRMS relapse for both naïve and experienced patients 1st year | 373 | 1.000 | 1.000 | 1.000 | Neurologists’opinion |
| 125 | Vol_Haepatic_test_ALT_given_RRMS_relapse_1st_year | Parameters (L508) | Number of ALT tests given RRMS relapse for both naïve and experienced patients 1st year | 373 | 1.000 | 1.000 | 1.000 | Neurologists’opinion |
| 126 | Vol_Haepatic_test_AST_given_RRMS_relapse_1st_year | Parameters (L509) | Number of AST tests given RRMS relapse for both naïve and experienced patients 1st year | 373 | 1.000 | 1.000 | 1.000 | Neurologists’opinion |
| 127 | Vol_Haepatic_test_gGT_given_RRMS_relapse_1st_year | Parameters (L510) | Number of AST tests given RRMS relapse for both naïve and experienced patients 1st year | 373 | 1.000 | 1.000 | 1.000 | Neurologists’opinion |
| 128 | Vol_Brain_MRI_C_given_RRMS_relapse_1st_year | Parameters (L511) | Number of brain MRIs with contrast given RRMS relapse for both naïve and experienced patients 1st year | 373 | 1.000 | 1.000 | 1.000 | Neurologists’opinion |
| 129 | Vol_Brain_plus_spinal_cord_MRI_C_given_RRMS_relapse_1st_year | Parameters (L512) | Number of brain plus spinal cord MRIs with contrast given RRMS relapse for both naïve and experienced patients 1st year | 373 | 1.000 | 1.000 | 1.000 | Neurologists’opinion |
| 130 | Vol_Neurologist_visit_given_RRMS_relapse_1st_year | Parameters (L513) | Number of neurologist visits given RRMS relapse for both naïve and experienced patients 1st year | 373 | 1.000 | 1.000 | 1.000 | Neurologists’opinion |
| 131 | Posology_corticosteroids_given_RRMS_relapse_2_7_year_Teriflunomide | Parameters (L514) | Posology corticosteroids (grams *per diem*) given RRMS relapse for both naïve and experienced patients years 2-7 | 373 | 0.950 | 0.950 | 0.950 | Neurologists’opinion |
| 132 | Duration_corticosteroids_given_RRMS_relapse_2_7_year_Teriflunomide | Parameters (L515) | Threrapy duration (days) given RRMS relapse for both naïve and experienced patients years 2-7 | 373 | 4.750 | 4.750 | 4.750 | Neurologists’opinion |
| 133 | Vol_Blood_test_given_RRMS_relapse_2_7_year | Parameters (L516) | Number of full blood tests given RRMS relapse for both naïve and experienced patients years 2-7 | 373 | 1.000 | 1.000 | 1.000 | Neurologists’opinion |

**Table SI12 – Materials and Methods - Parameters with no statistical distribution for base case analysis, OWSA and PSA**

| **Parameter number** | **Parameter**  **name** | **Spreadsheet**  **(point estimate cell)** | **Parameter**  **description** | **Observations** | **Point estimate** | **LL range** | **UL range** | **Source** |
| --- | --- | --- | --- | --- | --- | --- | --- | --- |
| **RRMS naïve and experienced patients on Teriflunomide** | | | | | | | | |
| **Healthcare resources - Volume** | | | | | | | | |
| **RRMS relapse - Management** | | | | | | | | |
| 134 | Vol_metabolic_test_blood_sugar_given_RRMS_relapse_2_7_year | Parameters (L517) | Number of blood sugar tests given RRMS relapse for both naïve and experienced patients years 2-7 | 373 | 1.000 | 1.000 | 1.000 | Neurologists’opinion |
| 135 | Vol_Kidney_test_Creatinine_given_RRMS_relapse_2_7_year | Parameters (L518) | Number of creatinine tests given RRMS relapse for both naïve and experienced patients years 2-7 | 373 | 1.000 | 1.000 | 1.000 | Neurologists’opinion |
| 136 | Vol_Kidney_test_Urine_given_RRMS_relapse_2_7_year | Parameters (L519) | Number of urine tests given RRMS relapse for both naïve and experienced patients years 2-7 | 373 | 1.000 | 1.000 | 1.000 | Neurologists’opinion |
| 137 | Vol_Haepatic_test_ALT_given_RRMS_relapse_2_7_year | Parameters (L520) | Number of ALT tests given RRMS relapse for both naïve and experienced patients years 2-7 | 373 | 1.000 | 1.000 | 1.000 | Neurologists’opinion |
| 138 | Vol_Haepatic_test_AST_given_RRMS_relapse_2_7_year | Parameters (L521) | Number of AST tests given RRMS relapse for both naïve and experienced patients years 2-7 | 373 | 1.000 | 1.000 | 1.000 | Neurologists’opinion |
| 139 | Vol_Haepatic_test_gGT_given_RRMS_relapse_2_7_year | Parameters (L522) | Number of gGT tests given RRMS relapse for both naïve and experienced patients years 2-7 | 373 | 1.000 | 1.000 | 1.000 | Neurologists’opinion |
| 140 | Vol_Brain_MRI_C_given_RRMS_relapse_2_7_year | Parameters (L523) | Number of brain MRIs with contrast given RRMS relapse for both naïve and experienced patients years 2-7 | 373 | 1.000 | 1.000 | 1.000 | Neurologists’opinion |
| 141 | Vol_Brain_plus_spinal_cord_MRI_C_given_RRMS_relapse_2_7_year | Parameters (L524) | Number of brain plus spinal cord MRIs with contrast given RRMS relapse for both naïve and experienced patients years 2-7 | 373 | 1.000 | 1.000 | 1.000 | Neurologists’opinion |
| 142 | Vol_Neurologist_visit_given_RRMS_relapse_2_7_year | Parameters (L525) | Number of neurologist visits given RRMS relapse for both naïve and experienced patients years 2-7 | 373 | 1.000 | 1.000 | 1.000 | Neurologists’opinion |
| **RRMS relapse – Follow-up** | | | | | | | | |
| 143 | Vol_Brain_MRI_fu_RRMS_relapse_1st_year_Teriflunomide | Parameters (L527) | Number of brain MRIs with contrast for follow-up given RRMS relapse for both naïve and experienced patients 1st year | 373 | 2.000 | 2.000 | 2.000 | Neurologists’opinion |
| 144 | Vol_Brain_plus_spinal_cord_MRI_fu_RRMS_relapse_1st_year_Teriflunomide | Parameters (L528) | Number of brain plus spinal cord MRIs with contrast for follow-up given RRMS relapse for both naïve and experienced patients 1st year | 373 | 1.000 | 1.000 | 1.000 | Neurologists’opinion |
| 145 | Vol_Neurologist_visit_fu_RRMS_relapse_1st_year_Teriflunomide | Parameters (L529) | Number of neurologist visits for follow-up given RRMS relapse for both naïve and experienced patients 1st year | 373 | 1.500 | 1.500 | 1.500 | Neurologists’opinion |
| 146 | Vol_Blood_test_fu_RRMS_relapse_2_7_year_Teriflunomide | Parameters (L530) | Number of blood tests for follow-up given RRMS relapse for both naïve and experienced patients years 2-7 | 373 | 4.000 | 4.000 | 4.000 | Neurologists’opinion |
| 147 | Vol_Kidney_test_Creatinine_fu_RRMS_relapse_2_7_year_Teriflunomide | Parameters (L531) | Number of creatinine tests for follow-up given RRMS relapse for both naïve and experienced patients years 2-7 | 373 | 4.000 | 4.000 | 4.000 | Neurologists’opinion |
| 148 | Vol_ Haepatic_test_ALT_fu_RRMS_relapse_2_7_year_Teriflunomide | Parameters (L532) | Number of ALT tests for follow-up given RRMS relapse for both naïve and experienced patients years 2-7 | 373 | 4.000 | 4.000 | 4.000 | Neurologists’opinion |
| 149 | Vol_ Haepatic_test_AST_fu_RRMS_relapse_2_7_year_Teriflunomide | Parameters (L533) | Number of AST tests for follow-up given RRMS relapse for both naïve and experienced patients years 2-7 | 373 | 4.000 | 4.000 | 4.000 | Neurologists’opinion |
| 150 | Vol_ Haepatic_test_gGT_fu_RRMS_relapse_2_7_year_Teriflunomide | Parameters (L534) | Number of gGT tests for follow-up given RRMS relapse for both naïve and experienced patients years 2-7 | 373 | 4.000 | 4.000 | 4.000 | Neurologists’opinion |
| 151 | Vol_Neurologist_visit_fu_RRMS_relapse_2_7_year_Teriflunomide | Parameters (L525) | Number of neurologist visits for follow-up given RRMS relapse for both naïve and experienced patients years 2-7 | 373 | 1.500 | 1.500 | 1.500 | Neurologists’opinion |
| 152 | Vol_Brain_plus_spinal_cord_MRI_fu_RRMS_relapse_1st_year_Teriflunomide | Parameters (L528) | Number of brain plus spinal cord MRIs with contrast for follow-up given RRMS relapse for both naïve and experienced patients 1st year | 373 | 1.000 | 1.000 | 1.000 | Neurologists’opinion |
| 153 | Vol_Brain_plus_spinal_cord_MRI_fu_RRMS_relapse_2_7_year_Teriflunomide Teriflunomide | Parameters (L537) | Number of brain plus spinal cord MRIs with contrast for follow-up given RRMS relapse for both naïve and experienced patients years 2-7 | 373 | 1.000 | 1.000 | 1.000 | Neurologists’opinion |

**Table SI13 – Materials and Methods - Parameters with no statistical distribution for base case analysis, OWSA and PSA**

| **Parameter number** | **Parameter**  **name** | **Spreadsheet**  **(point estimate cell)** | **Parameter**  **description** | **Observations** | **Point estimate** | **LL range** | **UL range** | **Source** |
| --- | --- | --- | --- | --- | --- | --- | --- | --- |
| **RRMS naïve and experienced patients on Teriflunomide** | | | | | | | | |
| **Healthcare resources - Volume** | | | | | | | | |
| **Adverse events - Teriflunomide** | | | | | | | | |
| 154 | Tablets_hair_thinning_1st_year_only_Teriflunomide | Parameters (L539) | Posology (tablets *per diem*) threrapy for hair thinning for both naïve and experienced RRMS patients for adverse events during Teriflunomide treatment 1st year | 373 | 1.000 | 1.000 | 1.000 | Neurologists’opinion |
| 155 | Posology_drug_rachialgy_1st_year_Teriflunomide | Parameters (L541) | Posology (milligrams *per diem*) threrapy for rachialgy for both naïve and experienced RRMS patients for adverse events during Teriflunomide treatment 1st year | 373 | 1.000 | 1.000 | 1.000 | Neurologists’opinion |
| 156 | Posology_drug_rachialgy_2_7_year_Teriflunomide | Parameters (L543) | Posology (milligrams *per diem*) threrapy for rachialgy for both naïve and experienced RRMS patients for adverse events during Teriflunomide treatment years 2-7 | 373 | 1.000 | 1.000 | 1.000 | Neurologists’opinion |
| 157 | Posology_drug_hypertension_1st_year_Teriflunomide | Parameters (L541) | Posology (milligrams *per diem*) threrapy for hypertension for both naïve and experienced RRMS patients for adverse events during Teriflunomide treatment 1st year | 373 | 20 | 20 | 20 | Neurologists’opinion |
| 158 | Therapy_dur_hypertension_1st_year_Teriflunomide | Parameters (L546) | Therapy duration (days) hypertension for both naïve and experienced RRMS patients for adverse events during Teriflunomide treatment 1st year | 373 | 365.25 | 365.25 | 365.25 | Neurologists’opinion; [SI4.] |
| 159 | Vol_Holter_press_given_hypertension_1st_year_Teriflunomide | Parameters (L547) | Number of Holter tests given hypertension for both naïve and experienced RRMS patients for adverse events during Teriflunomide treatment 1st year | 373 | 1.000 | 1.000 | 1.000 | Neurologists’opinion |
| 160 | Vol_ECG_given_hypertension_1st_year_Teriflunomide | Parameters (L548) | Number of ECG tests given hypertension for both naïve and experienced RRMS patients for adverse events during Teriflunomide treatment 1st year | 373 | 1.000 | 1.000 | 1.000 | Neurologists’opinion |
| 161 | Vol_Echocardiogram_given_hypertension_1st_year_Teriflunomide | Parameters (L549) | Number of echocardiogram tests given hypertension for both naïve and experienced RRMS patients for adverse events during Teriflunomide treatment 1st year | 373 | 1.000 | 1.000 | 1.000 | Neurologists’opinion |
| 162 | Posology_drug_hypertension_2_7_year_Teriflunomide | Parameters (L550) | Posology (milligrams *per diem*) threrapy for hypertension for both naïve and experienced RRMS patients for adverse events during Teriflunomide treatment years 2-7 | 373 | 20 | 20 | 20 | Neurologists’opinion |
| 163 | Therapy_dur_hypertension_2_7_year_Teriflunomide | Parameters (L551) | Therapy duration hypertension (days) for both naïve and experienced RRMS patients for adverse events during Teriflunomide treatment years 2-7 | 373 | 365.25 | 365.25 | 365.25 | Neurologists’opinion; [SI4.] |
| 164 | Vol_Cardiologist_visit_given_hypertension_1st_year_Teriflunomide | Parameters (L552) | Number of cardiologist visits given hypertension for both naïve and experienced RRMS patients for adverse events during Teriflunomide treatment 1st year | 373 | 1.000 | 1.000 | 1.000 | Neurologists’opinion |
| 165 | Vol_Holter_press_given_hypertension_2_7_year_Teriflunomide | Parameters (L553) | Number of Holter tests given hypertension for both naïve and experienced RRMS patients for adverse events during Teriflunomide treatment years 2-7 | 373 | 1.000 | 1.000 | 1.000 | Neurologists’opinion |
| 166 | Vol_Cardiologist_visit_given_hypertension_2_7_year_Teriflunomide | Parameters (L554) | Number of cardiologist visit sgiven hypertension for both naïve and experienced RRMS patients for adverse events during Teriflunomide treatment years 2-7 | 373 | 1.000 | 1.000 | 1.000 | Neurologists’opinion |
| 167 | Posology_drug_minor_depression_1st_year_Teriflunomide | Parameters (L555) | Posology (milligrams *per diem*) threrapy for minor depression for both naïve and experienced RRMS patients for adverse events during Teriflunomide treatment 1st year | 373 | 50 | 50 | 50 | Neurologists’opinion |
| 168 | Therapy_dur_minor_depression_1st_year_Teriflunomide | Parameters (L556) | Therapy duration (days) minor depression for both naïve and experienced RRMS patients for adverse events during Teriflunomide treatment 1st year | 373 | 365.25 | 365.25 | 365.25 | Neurologists’opinion; [SI4.] |
| 169 | Posology_drug_minor_depression_2_7_year_Teriflunomide | Parameters (L557) | Posology (milligrams *per diem*) threrapy for minor depression for both naïve and experienced RRMS patients for adverse events during Teriflunomide treatment years 2-7 | 373 | 50 | 50 | 50 | Neurologists’opinion |
| 170 | Therapy_dur_minor_depression_2_7_year_Teriflunomide | Parameters (L558) | Therapy duration (days) minor depression for both naïve and experienced RRMS patients for adverse events during Teriflunomide treatment years 2-7 | 373 | 365.25 | 365.25 | 365.25 | Neurologists’opinion; [SI4.] |
| 171 | Posology_drug_haepatoprotector_1st_year_Teriflunomide | Parameters (L559) | Posology (milligrams *per diem*) threrapy for haepatoprotection for both naïve and experienced RRMS patients for adverse events during Teriflunomide treatment 1st year | 373 | 450 | 450 | 450 | Neurologists’opinion |
| 172 | Posology_drug_haepatoprotector_2_7_year_Teriflunomide | Parameters (L561) | Posology (milligrams *per diem*) threrapy for haepatoprotectionfor both naïve and experienced RRMS patients for adverse events during Teriflunomide treatment years 2-7 | 373 | 450 | 450 | 450 | Neurologists’opinion |

**Table SI14 – Materials and Methods - Parameters with no statistical distribution for base case analysis, OWSA and PSA**

| **Parameter number** | **Parameter**  **name** | **Spreadsheet**  **(point estimate cell)** | **Parameter**  **description** | **Observations** | **Point estimate** | **LL**  **range** | **UL range** | **Source** |
| --- | --- | --- | --- | --- | --- | --- | --- | --- |
| **RRMS naïve and experienced patients on Teriflunomide** | | | | | | | | |
| **Healthcare resources - Volume** | | | | | | | | |
| **Adverse events - Teriflunomide** | | | | | | | | |
| 173 | Vol_Haepatic_ultrasound_given_hepatoprotection_1st_year_Teriflunomide | Parameters (L563) | Number of haepatic ultrasounds given hepatoprotection for both naïve and experienced patients 1st year | 373 | 1.000 | 1.000 | 1.000 | Neurologists’opinion |
| 174 | Vol_Haepatic_test_ALT_given_hepatoprotection_1st_year_Teriflunomide | Parameters (L564) | Number of ALT tests given hepatoprotection for both naïve and experienced patients 1st year | 373 | 1.000 | 1.000 | 1.000 | Neurologists’opinion |
| 175 | Vol_Haepatic_test_AST_given_hepatoprotection_1st_year_Teriflunomide | Parameters (L565) | Number of AST tests given hepatoprotection for both naïve and experienced patients 1st year | 373 | 1.000 | 1.000 | 1.000 | Neurologists’opinion |
| 176 | Vol_Haepatic_test_gGT_given_hepatoprotection_1st_year_Teriflunomide | Parameters (L566) | Number of gGT tests given hepatoprotection for both naïve and experienced patients 1st year | 373 | 1.000 | 1.000 | 1.000 | Neurologists’opinion |
| 177 | Vol_Haepatic_test_Total_bilirubin_given_hepatoprotection_1st_year_Teriflunomide | Parameters (L567) | Number of total bilirubin tests given hepatoprotection for both naïve and experienced patients 1st year | 373 | 1.000 | 1.000 | 1.000 | Neurologists’opinion |
| 178 | Vol_Haepatic_test_albumine_given_hepatoprotection_1st_year_Teriflunomide | Parameters (L568) | Number of albumine tests given hepatoprotection for both naïve and experienced patients 1st year | 373 | 1.000 | 1.000 | 1.000 | Neurologists’opinion |
| 179 | Vol_Haepatologist_visit_given_hepatoprotection_1st_year_Teriflunomide | Parameters (L569) | Number of haepatologist visits given hepatoprotection for both naïve and experienced patients 1st year | 373 | 1.000 | 1.000 | 1.000 | Neurologists’opinion |
| 180 | Vol_Haepatic_ultrasound_given_hepatoprotection_2_7_year_Teriflunomide | Parameters (L570) | Number of haepatic ultrasounds given hepatoprotection for both naïve and experienced patients years 2-7 | 373 | 1.000 | 1.000 | 1.000 | Neurologists’opinion |
| 181 | Vol_Haepatic_test_ALT_given_hepatoprotection_2_7_year_Teriflunomide | Parameters (L571) | Number of ALT tests given hepatoprotection for both naïve and experienced patients years 2-7 | 373 | 1.000 | 1.000 | 1.000 | Neurologists’opinion |
| 182 | Vol_Haepatic_test_AST_given_hepatoprotection_2_7_year_Teriflunomide | Parameters (L572) | Number of AST tests given hepatoprotection for both naïve and experienced patients years 2-7 | 373 | 1.000 | 1.000 | 1.000 | Neurologists’opinion |
| 183 | Vol_Haepatic_test_gGT_given_hepatoprotection_2_7_year_Teriflunomide | Parameters (L573) | Number of gGT tests given hepatoprotection for both naïve and experienced patients years 2-7 | 373 | 1.000 | 1.000 | 1.000 | Neurologists’opinion |
| 184 | Vol_Haepatic_test_Total_bilirubin_given_hepatoprotection_2_7_year_Teriflunomide | Parameters (L574) | Number of total bilirubin tests given hepatoprotection for both naïve and experienced patients years 2-7 | 373 | 1.000 | 1.000 | 1.000 | Neurologists’opinion |
| 185 | Vol_Haepatic_test_albumine_given_hepatoprotection_2_7_year_Teriflunomide | Parameters (L575) | Number of albumine tests given hepatoprotection for both naïve and experienced patients years 2-7 | 373 | 1.000 | 1.000 | 1.000 | Neurologists’opinion |
| 186 | Vol_Haepatologist_visit_given_hepatoprotection_2_7_year_Teriflunomide | Parameters (L576) | Number of haepatologist visits given hepatoprotection for both naïve and experienced patients years 2-7 | 373 | 1.000 | 1.000 | 1.000 | Neurologists’opinion |
| 187 | Posology_drug_infection_1st_year_Teriflunomide | Parameters (L577) | Posology (milligrams *per diem*) threrapy for infection for both naïve and experienced RRMS patients for adverse events during Teriflunomide treatment 1st year | 373 | 1000 | 1000 | 1000 | Neurologists’opinion |
| 188 | Posology_drug_infection_2_7_year_Teriflunomide | Parameters (L579) | Posology (milligrams *per diem*) threrapy for infection for both naïve and experienced RRMS patients for adverse events during Teriflunomide treatment years 2-7 | 373 | 1000 | 1000 | 1000 | Neurologists’opinion |
| 189 | Vol_Infectivologist_visit_given_infection_1st_year_Teriflunomide | Parameters (L581) | Number of infectivologist visits for infection for both naïve and experienced RRMS patients for adverse events during Teriflunomide treatment 1st year | 373 | 1.000 | 1.000 | 1.000 | Neurologists’opinion |
| **Switch to other DMTs due to Teriflunomide ineffectiveness in naïve patients - All DMTs** | | | | | | | | |
| **Healthcare resources - Volume** | | | | | | | | |
| **Pre-treatment assessment - Natalizumab** | | | | | | | | |
| 190 | Vol_pre_Natalizumab_full_blood_count_given_fail_naïve_2_7_year_Teriflunomide | Parameters (L584) | Number of full blood tests pre-treatment assessment Natalizumab given poor response to Teriflunomide naïve patients years 2_7 | 15 | 1.000 | 1.000 | 1.000 | Neurologists’opinion |
| 191 | Vol_pre_Natalizumab_haepatic_test_ALT_given_fail_naïve_2_7_year_Teriflunomide | Parameters (L585) | Number of ALT tests pre-treatment assessment Natalizumab given poor response to Teriflunomide naïve patients years 2_7 | 15 | 1.000 | 1.000 | 1.000 | Neurologists’opinion |
| 192 | Vol_pre_Natalizumab_haepatic_test_AST_given_fail_naïve_2_7_year_Teriflunomide | Parameters (L586) | Number of AST tests pre-treatment assessment Natalizumab given poor response to Teriflunomide naïve patients years 2_7 | 15 | 1.000 | 1.000 | 1.000 | Neurologists’opinion |
| 193 | Vol_pre_Natalizumab_haepatic_test_gGT_given_fail_naïve_2_7_year_Teriflunomide | Parameters (L587) | Number of AST tests pre-treatment assessment Natalizumab given poor response to Teriflunomide naïve patients years 2_7 | 15 | 1.000 | 1.000 | 1.000 | Neurologists’opinion |

**Table SI15 – Materials and Methods - Parameters with no statistical distribution for base case analysis, OWSA and PSA**

| **Parameter number** | **Parameter**  **name** | **Spreadsheet**  **(point estimate cell)** | **Parameter**  **description** | **Observations** | **Point estimate** | **LL range** | **UL**  **range** | **Source** |
| --- | --- | --- | --- | --- | --- | --- | --- | --- |
| **RRMS naïve and experienced patients on Teriflunomide** | | | | | | | | |
| **Switch to other DMTs due to Teriflunomide ineffectiveness in naïve patients - All DMTs** | | | | | | | | |
| **Healthcare resources - Volume** | | | | | | | | |
| **Pre-treatment assessment - Natalizumab** | | | | | | | | |
| 194 | Vol_pre_Natalizumab_haepatic_test_total_bilirubin_given_fail_naïve_2_7_year_Teriflunomide | Parameters (L588) | Number of total bilirubin tests pre-treatment assessment Natalizumab given poor response to Teriflunomide naïve patients years 2_7 | 15 | 1.000 | 1.000 | 1.000 | Neurologists’opinion |
| 195 | Vol_pre_Natalizumab_kidney_test_creatinine_given_fail_naïve_2_7_year_Teriflunomide | Parameters (L589) | Number of creatinine tests pre-treatment assessment Natalizumab given poor response to Teriflunomide naïve patients years 2_7 | 15 | 1.000 | 1.000 | 1.000 | Neurologists’opinion |
| 196 | Vol_pre_Natalizumab_kidney_test_urea_given_fail_naïve_2_7_year_Teriflunomide | Parameters (L590) | Number of urea tests pre-treatment assessment Natalizumab given poor response to Teriflunomide naïve patients years 2_7 | 15 | 1.000 | 1.000 | 1.000 | Neurologists’opinion |
| 197 | Vol_pre_Natalizumab_kidney_test_urine_given_fail_naïve_2_7_year_Teriflunomide | Parameters (L591) | Number of urine tests pre-treatment assessment Natalizumab given poor response to Teriflunomide naïve patients years 2_7 | 15 | 1.000 | 1.000 | 1.000 | Neurologists’opinion |
| 198 | Vol_pre_Natalizumab_sierology_test_JCV_given_fail_naïve_2_7_year_Teriflunomide | Parameters (L592) | Number of JCV tests pre-treatment assessment Natalizumab given poor response to Teriflunomide naïve patients years 2_7 | 15 | 1.000 | 1.000 | 1.000 | Neurologists’opinion |
| **During treatment assessment - Natalizumab** | | | | | | | | |
| 199 | Vol_during_Natalizumab_full_blood_count_given_fail_naïve_2_7_year_Teriflunomide | Parameters (L594) | Number of full blood tests during Natalizumab treatment given poor response to Teriflunomide naïve patients years 2_7 | 15 | 10.400 | 10.400 | 10.400 | Neurologists’opinion |
| 200 | Vol_during_Natalizumab_haepatic_test_ALT_given_fail_naïve_2_7_year_Teriflunomide | Parameters (L595) | Number of ALT tests during Natalizumab treatment given poor response to Teriflunomide naïve patients years 2_7 | 15 | 10.400 | 10.400 | 10.400 | Neurologists’opinion |
| 201 | Vol_during_Natalizumab_haepatic_test_AST_given_fail_naïve_2_7_year_Teriflunomide | Parameters (L596) | Number of AST tests during Natalizumab treatment given poor response to Teriflunomide naïve patients years 2_7 | 15 | 10.400 | 10.400 | 10.400 | Neurologists’opinion |
| 202 | Vol_during_Natalizumab_haepatic_test_gGT_given_fail_naïve_2_7_year_Teriflunomide | Parameters (L597) | Number of gGT tests during Natalizumab treatment given poor response to Teriflunomide naïve patients years 2_7 | 15 | 10.400 | 10.400 | 10.400 | Neurologists’opinion |
| 203 | Vol_during_Natalizumab_haepatic_test_total_bilirubin_given_fail_naïve_2_7_year_Teriflunomide | Parameters (L598) | Number of total bilirubin tests during Natalizumab treatment given poor response to Teriflunomide naïve patients years 2_7 | 15 | 10.400 | 10.400 | 10.400 | Neurologists’opinion |
| 204 | Vol_during_Natalizumab_kidney_test_creatinine_given_fail_naïve_2_7_year_Teriflunomide | Parameters (L599) | Number of creeatinine tests during Natalizumab treatment given poor response to Teriflunomide naïve patients years 2_7 | 15 | 10.400 | 10.400 | 10.400 | Neurologists’opinion |
| 205 | Vol_during_Natalizumab_kidney_test_urea_given_fail_naïve_2_7_year_Teriflunomide | Parameters (L600) | Number of urea tests during Natalizumab treatment given poor response to Teriflunomide naïve patients years 2_7 | 15 | 10.400 | 10.400 | 10.400 | Neurologists’opinion |
| 206 | Vol_during_Natalizumab_kidney_test_urine_given_fail_naïve_2_7_year_Teriflunomide | Parameters (L601) | Number of urine tests during Natalizumab treatment given poor response to Teriflunomide naïve patients years 2_7 | 15 | 10.400 | 10.400 | 10.400 | Neurologists’opinion |
| 207 | Vol_during_Natalizumab_sierology_test_JCV_given_fail_naïve_2_7_year_Teriflunomide | Parameters (L602) | Number of JCV tests during Natalizumab treatment given poor response to Teriflunomide naïve patients years 2_7 | 15 | 2.000 | 2.000 | 2.000 | Neurologists’opinion |
| **Premedication - Natalizumab** | | | | | | | | |
| 208 | Posology_corticosteroids_premed_Natalizumab_given_fail_naïve_2_7_year_Teriflunomide | Parameters (L604) | Posology (grams *per* year) corticosteroids premedicationNatalizumab treatment given poor response to Teriflunomide naïve patients years 2_7 | 15 | 0.690 | 0.690 | 0.690 | Neurologists’opinion |
| 209 | Vol_min_year_premed_nurse_Natalizumab_given_fail_naïve_2_7_year_Teriflunomide | Parameters (L605) | Nurse time (minutes *per* year) for premedicationNatalizumab treatment given poor response to Teriflunomide naïve patients years 2_7 | 15 | 0.517 | 0.517 | 0.517 | Neurologists’opinion |

**Table SI16 – Materials and Methods - Parameters with no statistical distribution for base case analysis, OWSA and PSA**

| **Parameter number** | **Parameter**  **name** | **Spreadsheet**  **(point estimate cell)** | **Parameter**  **description** | **Observations** | **Point estimate** | **LL**  **range** | **UL**  **range** | **Source** |
| --- | --- | --- | --- | --- | --- | --- | --- | --- |
| **RRMS naïve and experienced patients on Teriflunomide** | | | | | | | | |
| **Switch to other DMTs due to Teriflunomide ineffectiveness in naïve patients - All DMTs** | | | | | | | | |
| **Healthcare resources - Volume** | | | | | | | | |
| **Administration time - Natalizumab** | | | | | | | | |
| 210 | Vol_min_adm_pharma_Natalizumab_given_fail_naïve_2_7_year_Teriflunomide | Parameters (L607) | Pharmacist time (minutes *per* administration) for administration Natalizumab treatment given poor response to Teriflunomide naïve patients years 2_7 | 15 | 60.000 | 60.000 | 60.000 | Neurologists’opinion |
| **Administration - Natalizumab** | | | | | | | | |
| 211 | Posology_Natalizumab_given_fail_naïve_2_7_year_Teriflunomide | Parameters (L609) | Posology (milligrams *per* administration) Natalizumab treatment given poor response to Teriflunomide naïve patients years 2_7 | 15 | 300.000 | 300.000 | 300.000 | Neurologists’opinion |
| 212 | Vol_administrations_Natalizumab_given_fail_naïve_2_7_year_Teriflunomide | Parameters (L610) | Number of administrations per *year* Natalizumab treatment given poor response to Teriflunomide naïve patients years 2_7 | 15 | 11.595 | 11.595 | 11.595 | Neurologists’opinion |
| **Administration disposables - Natalizumab** | | | | | | | | |
| 213 | Vol_adm_physio_Natalizumab_given_fail_naïve_2_7_year_Teriflunomide | Parameters (L612) | Number of physiological solution administrations Natalizumab given poor response to Teriflunomide naïve patients years 2_7 | 15 | 1.000 | 1.000 | 1.000 | Neurologists’opinion |
| 214 | Vol_adm_iv_line_Natalizumab_given_fail_naïve_2_7_year_Teriflunomide | Parameters (L613) | Number of iv line for Natalizumab administrations given poor response to Teriflunomide naïve patients years 2_7 | 15 | 1.000 | 1.000 | 1.000 | Neurologists’opinion |
| 215 | Vol_adm_needle_Natalizumab_given_fail_naïve_2_7_year_Teriflunomide | Parameters (L614) | Number of needle for Natalizumab administrations given poor response to Teriflunomide naïve patients years 2_7 | 15 | 1.000 | 1.000 | 1.000 | Neurologists’opinion |
| 216 | Vol_adm_antiseptic_cc_Natalizumab_given_fail_naïve_2_7_year_Teriflunomide | Parameters (L615) | Volume (cubic centimeter s *per* administration) of antiseptic for Natalizumab administrations given poor response to Teriflunomide naïve patients years 2_7 naïve patients years 2_7 | 15 | 10.000 | 10.000 | 10.000 | Neurologists’opinion |
| 217 | Vol_adm_CVC_Natalizumab_given_fail_naïve_2_7_year_Teriflunomide | Parameters (L616) | Number of CVCs for Natalizumab administration given poor response to Teriflunomide naïve patients years 2_7 naïve patients years 2_7 | 15 | 1.000 | 1.000 | 1.000 | Neurologists’opinion |
| 218 | Vol_adm_gauze_Natalizumab_given_fail_naïve_2_7_year_Teriflunomide | Parameters (L617) | Number of gauzes for Natalizumab administration given poor response to Teriflunomide naïve patients years 2_7 naïve patients years 2_7 | 15 | 2.000 | 2.000 | 2.000 | Neurologists’opinion |
| **Postadministration disposables - Natalizumab** | | | | | | | | |
| 219 | Vol_postad_physio_Natalizumab_given_fail_naïve_2_7_year_Teriflunomide | Parameters (L619) | Number of physiological solutions postadministration Natalizumab given poor response to Teriflunomide naïve patients years 2_7 | 15 | 1.000 | 1.000 | 1.000 | Neurologists’opinion |
| **Postadministration time - Natalizumab** | | | | | | | | |
| 220 | Vol_postad_time_nurse_Natalizumab_given_fail_naïve_2_7_year_Teriflunomide | Parameters (L621) | Number of nurse time (minutes) for administration Natalizumab given poor response to Teriflunomide naïve patients years 2_7 | 15 | 60.000 | 60.000 | 60.000 | Neurologists’opinion |
| **Pre-treatment assessment - Fingolimod** | | | | | | | | |
| 221 | Vol_pre_Fingolimod_ECG_given_fail_naïve_2_7_year_Teriflunomide | Parameters (L624) | Number of ECG tests pre-treatment assessment Fingolimod given poor response to Teriflunomide naïve patients years 2_7 | 11 | 1.000 | 1.000 | 1.000 | Neurologists’opinion |
| 222 | Vol_pre_Fingolimod_OCT_given_fail_naïve_2_7_year_Teriflunomide | Parameters (L625) | Number of OCT tests pre-treatment assessment Fingolimod given poor response to Teriflunomide naïve patients years 2_7 | 11 | 1.000 | 1.000 | 1.000 | Neurologists’opinion |
| 223 | Vol_pre_Fingolimod_sierology_test_zooster_given_fail_naïve_2_7_year_Teriflunomide | Parameters (L625) | Number of zooster tests pre-treatment assessment Fingolimod given poor response to Teriflunomide naïve patients years 2_7 | 11 | 1.000 | 1.000 | 1.000 | Neurologists’opinion |
| 224 | Vol_pre_Fingolimod_sierology_test_zooster_given_fail_naïve_2_7_year_Teriflunomide | Parameters (L626) | Number of zooster tests pre-treatment assessment Fingolimod given poor response to Teriflunomide naïve patients years 2_7 | 11 | 1.000 | 1.000 | 1.000 | Neurologists’opinion |
| 225 | Vol_pre_Fingolimod_dermatologist_visit_given_fail_naïve_2_7_year_Teriflunomide | Parameters (L627) | Number of dermatologist visits pre-treatment assessment Fingolimod given poor response to Teriflunomide naïve patients years 2_7 | 11 | 1.000 | 1.000 | 1.000 | Neurologists’opinion |

**Table SI17 – Materials and Methods - Parameters with no statistical distribution for base case analysis, OWSA and PSA**

| **Parameter number** | **Parameter**  **name** | **Spreadsheet**  **(point estimate cell)** | **Parameter**  **description** | **Observations** | **Point estimate** | **LL**  **range** | **UL range** | **Source** |
| --- | --- | --- | --- | --- | --- | --- | --- | --- |
| **RRMS naïve and experienced patients on Teriflunomide** | | | | | | | | |
| **Switch to other DMTs due to Teriflunomide ineffectiveness in naïve patients - All DMTs** | | | | | | | | |
| **Healthcare resources - Volume** | | | | | | | | |
| **Administration - Fingolimod** | | | | | | | | |
| 226 | Posology_Fingolimod_given_fail_naïve_2_7_year_Teriflunomide | Parameters (L629) | Posology (milligrams *per* administration) Fingolimod treatment given poor response to Teriflunomide naïve patients years 2_7 | 11 | 0.500 | 0.500 | 0.500 | Neurologists’opinion |
| 227 | Vol_administrations_Fingolimod_given_fail_naïve_2_7_year_Teriflunomide | Parameters (L630) | Number of administrations per *year* Fingolimod treatment given poor response to Teriflunomide naïve patients years 2_7 | 11 | 365.25 | 365.25 | 365.25 | Neurologists’opinion; [SI4.] |
| **Pre-treatment assessment - Cladribine** | | | | | | | | |
| 228 | Vol_pre_Cladribine_haepatic_test_ALT_given_fail_naïve_2_7_year_Teriflunomide | Parameters (L633) | Number of ALT tests pre-treatment assessment Cladribine given poor response to Teriflunomide naïve patients years 2_7 | 7 | 1.000 | 1.000 | 1.000 | Neurologists’opinion |
| 229 | Vol_pre_Cladribine_haepatic_test_AST_given_fail_naïve_2_7_year_Teriflunomide | Parameters (L634) | Number of AST tests pre-treatment assessment Cladribine given poor response to Teriflunomide naïve patients years 2_7 | 7 | 1.000 | 1.000 | 1.000 | Neurologists’opinion |
| 230 | Vol_pre_Cladribine_haepatic_test_gGT_given_fail_naïve_2_7_year_Teriflunomide | Parameters (L635) | Number of gGT tests pre-treatment assessment Cladribine given poor response to Teriflunomide naïve patients years 2_7 | 7 | 1.000 | 1.000 | 1.000 | Neurologists’opinion |
| 231 | Vol_pre_Cladribine_kidney_test_Creatinine_given_fail_naïve_2_7_year_Teriflunomide | Parameters (L636) | Number of creatinine tests pre-treatment assessment Cladribine given poor response to Teriflunomide naïve patients years 2_7 | 7 | 1.000 | 1.000 | 1.000 | Neurologists’opinion |
| 232 | Vol_pre_Cladribine_kidney_test_Urea_given_fail_naïve_2_7_year_Teriflunomide | Parameters (L637) | Number of urea tests pre-treatment assessment Cladribine given poor response to Teriflunomide naïve patients years 2_7 | 7 | 1.000 | 1.000 | 1.000 | Neurologists’opinion |
| 233 | Vol_pre_Cladribine_kidney_test_Urine_given_fail_naïve_2_7_year_Teriflunomide | Parameters (L638) | Number of urine tests pre-treatment assessment Cladribine given poor response to Teriflunomide naïve patients years 2_7 | 7 | 1.000 | 1.000 | 1.000 | Neurologists’opinion |
| 234 | Vol_pre_Cladribine_sierology_test_HBV_given_fail_naïve_2_7_year_Teriflunomide | Parameters (L639) | Number of HBV tests pre-treatment assessment Cladribine given poor response to Teriflunomide naïve patients years 2_7 | 7 | 1.000 | 1.000 | 1.000 | Neurologists’opinion |
| 235 | Vol_pre_Cladribine_sierology_test_HCV_given_fail_naïve_2_7_year_Teriflunomide | Parameters (L640) | Number of HCV tests pre-treatment assessment Cladribine given poor response to Teriflunomide naïve patients years 2_7 | 7 | 1.000 | 1.000 | 1.000 | Neurologists’opinion |
| 236 | Vol_pre_Cladribine_sierology_test_zooster_given_fail_naïve_2_7_year_Teriflunomide | Parameters (L641) | Number of zoster tests pre-treatment assessment Cladribine given poor response to Teriflunomide naïve patients years 2_7 | 7 | 1.000 | 1.000 | 1.000 | Neurologists’opinion |
| 237 | Vol_pre_Cladribine_sierology_test_quantiferon_given_fail_naïve_2_7_year_Teriflunomide | Parameters (L642) | Number of quantiferon test s pre-treatment assessment Cladribine given poor response to Teriflunomide naïve patients years 2_7 | 7 | 1.000 | 1.000 | 1.000 | Neurologists’opinion |
| **Administration - Cladribine** | | | | | | | | |
| 238 | Posology_Cladribine_given_fail_naïve_2_7_year_Teriflunomide | Parameters (L644) | Posology (milligrams *per* administration) Cladribine treatment given poor response to Teriflunomide naïve patients years 2_7 | 7 | 10.000 | 10.000 | 10.000 | Neurologists’opinion |
| 239 | Vol_administrations_Cladribine_given_fail_naïve_2_7_year_Teriflunomide | Parameters (L645) | Number of administrations per year Cladribine treatment given poor response to Teriflunomide naïve patients years 2_7 (Max: 2 years) | 7 | 12.000 | 12.000 | 12.000 | Neurologists’opinion |
| **Premedication - Ocrelizumab** | | | | | | | | |
| 240 | Posology_corticosteroids_premed_Ocrelizumab_given_fail_naïve_2_7_year_Teriflunomide | Parameters (L648) | Posology (grams *per* year) corticosteroids premedication Ocrelizumab treatment given poor response to Teriflunomide naïve patients years 2_7 | 6 | 1.000 | 1.000 | 1.000 | Neurologists’opinion |
| 241 | Posology_antihistaminics_premed_Ocrelizumab_given_fail_naïve_2_7_year_Teriflunomide | Parameters (L649) | Posology (milligrams *per* administration) antihistaminics premedication Ocrelizumab treatment given poor response to Teriflunomide naïve patients years 2_7 | 6 | 10.000 | 10.000 | 10.000 | Neurologists’opinion |

**Table SI18 – Materials and Methods - Parameters with no statistical distribution for base case analysis, OWSA and PSA**

| **Parameter number** | **Parameter**  **name** | **Spreadsheet**  **(point estimate cell)** | **Parameter**  **description** | **Observations** | **Point estimate** | **LL range** | **UL**  **range** | **Source** |
| --- | --- | --- | --- | --- | --- | --- | --- | --- |
| **RRMS naïve and experienced patients on Teriflunomide** | | | | | | | | |
| **Switch to other DMTs due to Teriflunomide ineffectiveness in naïve patients - All DMTs** | | | | | | | | |
| **Healthcare resources - Volume** | | | | | | | | |
| **Premedication - Ocrelizumab** | | | | | | | | |
| 242 | Vol_min_premed_nurse_Ocrelizumab_given_fail_naïve_2_7_year_Teriflunomide | Parameters (L650) | Nurse time (minutes *per* administration) for premedication Ocrelizumab treatment given poor response to Teriflunomide naïve patients years 2_7 | 6 | 60.000 | 60.000 | 60.000 | Neurologists’opinion |
| **Administration - Ocrelizumab** | | | | | | | | |
| 243 | Posology_Ocrelizuab_given_fail_naïve_2_7_year_Teriflunomide | Parameters (L652) | Posology (milligrams *per* administration) Ocrelizuab treatment given poor response to Teriflunomide naïve patients years 2_7 | 6 | 1200.000 | 1200.000 | 1200.000 | Neurologists’opinion |
| 244 | Vol_administrations_Ocrelizumab_given_fail_naïve_2nd_year_Teriflunomide | Parameters (L653) | Number of administrations per year Ocrelizuab treatment given poor response to Teriflunomide naïve patients 2^nd^ year | 6 | 3.000 | 3.000 | 3.000 | Neurologists’opinion |
| 245 | Vol_administrations_Ocrelizumab_given_fail_naïve_3_7_year_Teriflunomide | Parameters (L654) | Number of administrations per year Ocrelizuab treatment given poor response to Teriflunomide naïve patients years 3_7 | 6 | 2.000 | 12.000 | 2.000 | Neurologists’opinion |
| **Administration time - Ocrelizumab** | | | | | | | | |
| 246 | Vol_min_adm_nurse_Ocrelizumab_given_fail_naïve_2_7_year_Teriflunomide | Parameters (L656) | Nurse time (minutes *per* administration) for administration Ocrelizumab treatment given poor response to Teriflunomide naïve patients years 2_7 | 6 | 1.000 | 1.000 | 1.000 | Neurologists’opinion |
| **Administration disposables - Ocrelizumab** | | | | | | | | |
| 247 | Vol_adm_iv_line_Ocrelizumab_given_fail_naïve_2_7_year_Teriflunomide | Parameters (L658) | Number of iv lines for Ocrelizumab administration given poor response to Teriflunomide naïve patients years 2_7 | 6 | 1.000 | 1.000 | 1.000 | Neurologists’opinion |
| 248 | Vol_adm_needle_Ocrelizumab_given_fail_naïve_2_7_year_Teriflunomide | Parameters (L659) | Number of needles for Ocrelizumab administration given poor response to Teriflunomide naïve patients years 2_7 | 6 | 1.000 | 1.000 | 1.000 | Neurologists’opinion |
| 249 | Vol_adm_antiseptic_cc_Ocrelizumab_given_fail_naïve_2_7_year_Teriflunomide | Parameters (L660) | Volume (cubic centimeters *per* administration) of antiseptic for Ocrelizumab administration given poor response to Teriflunomide naïve patients years 2_7 naïve patients years 2_7 | 6 | 10.000 | 10.000 | 10.000 | Neurologists’opinion |
| **Postadministration disposables - Ocrelizumab** | | | | | | | | |
| 250 | Vol_postad_physio_Ocrelizumab_given_fail_naïve_2_7_year_Teriflunomide | Parameters (L662) | Number of physiological solutions postadministration Ocrelizumab given poor response to Teriflunomide naïve patients years 2_7 | 6 | 1.000 | 1.000 | 1.000 | Neurologists’opinion |
| **Postadministration time - Ocrelizumab** | | | | | | | | |
| 251 | Vol_postad_time_nurse_Ocrelizumab_given_fail_naïve_2_7_year_Teriflunomide | Parameters (L664) | Number of nurse time (minutes) for administration Ocrelizumab given poor response to Teriflunomide naïve patients years 2_7 | 6 | 90.000 | 90.000 | 90.000 | Neurologists’opinion |
| **Pre-treatment assessment - Alemtuzumab** | | | | | | | | |
| 252 | Vol_pre_Alemtuzumab_haepatic_test_ALT_given_fail_naïve_2_7_year_Teriflunomide | Parameters (L667) | Number of ALT tests pre-treatment assessment Alemtuzumab given poor response to Teriflunomide naïve patients years 2_7 | 5 | 1.000 | 1.000 | 1.000 | Neurologists’opinion |
| 253 | Vol_pre_Alemtuzumab_haepatic_test_AST_given_fail_naïve_2_7_year_Teriflunomide | Parameters (L668) | Number of AST tests pre-treatment assessment Alemtuzumab given poor response to Teriflunomide naïve patients years 2_7 | 5 | 1.000 | 1.000 | 1.000 | Neurologists’opinion |
| 254 | Vol_pre_Alemtuzumab_haepatic_test_gGT_given_fail_naïve_2_7_year_Teriflunomide | Parameters (L669) | Number of gGT tests pre-treatment assessment Alemtuzumab given poor response to Teriflunomide naïve patients years 2_7 | 5 | 1.000 | 1.000 | 1.000 | Neurologists’opinion |
| 255 | Vol_pre_Alemtuzumab_sierology_test_HBV_given_fail_naïve_2_7_year_Teriflunomide | Parameters (L670) | Number of HBV tests pre-treatment assessment Alemtuzumab given poor response to Teriflunomide naïve patients years 2_7 | 5 | 1.000 | 1.000 | 1.000 | Neurologists’opinion |

**Table SI19 – Materials and Methods - Parameters with no statistical distribution for base case analysis, OWSA and PSA**

| **Parameter number** | **Parameter**  **name** | **Spreadsheet**  **(point estimate cell)** | **Parameter**  **description** | **Observations** | **Point estimate** | **LL**  **range** | **UL**  **range** | **Source** |
| --- | --- | --- | --- | --- | --- | --- | --- | --- |
| **RRMS naïve and experienced patients on Teriflunomide** | | | | | | | | |
| **Switch to other DMTs due to Teriflunomide ineffectiveness in naïve patients - All DMTs** | | | | | | | | |
| **Healthcare resources - Volume** | | | | | | | | |
| **Switch to other drugs due to Teriflunomide ineffectiveness in naïve patients - All drugs** | | | | | | | | |
| **Pre-treatment assessment - Alemtuzumab** | | | | | | | | |
| 256 | Vol_pre_Alemtuzumab_sierology_test_HCV_given_fail_naïve_2_7_year_Teriflunomide | Parameters (L671) | Number of HBV tests pre-treatment assessment Alemtuzumab given poor response to Teriflunomide naïve patients years 2_7 | 5 | 1.000 | 1.000 | 1.000 | Neurologists’opinion |
| 257 | Vol_pre_Alemtuzumab_sierology_test_HPV_given_fail_naïve_2_7_year_Teriflunomide | Parameters (L672) | Number of HPV tests pre-treatment assessment Alemtuzumab given poor response to Teriflunomide naïve female patients years 2_7 | 5 | 1.000 | 1.000 | 1.000 | Neurologists’opinion |
| **Premedication - Alemtuzumab** | | | | | | | | |
| 258 | Posology_corticosteroids_premed_Alemtuzumab_given_fail_naïve_2_7_year_Teriflunomide | Parameters (L674) | Posology (grams *per* administration) corticosteroids premedication Alemtuzumab treatment given poor response to Teriflunomide naïve patients years 2_7 | 5 | 1.000 | 1.000 | 1.000 | Neurologists’opinion |
| 259 | Posology_antihistaminics_premed_Alemtuzumab_given_fail_naïve_2_7_year_Teriflunomide | Parameters (L675) | Posology (milligrams *per* administration) antihistaminics premedication Alemtuzumab treatment given poor response to Teriflunomide naïve patients years 2_7 | 5 | 10.000 | 10.000 | 10.000 | Neurologists’opinion |
| 260 | Vol_min_premed_nurse_Alemtuzumab_given_fail_naïve_2_7_year_Teriflunomide | Parameters (L676) | Nurse time (minutes *per* administration) for premeication Alemtuzumab treatment given poor response to Teriflunomide naïve patients years 2_7 | 5 | 60.000 | 60.000 | 60.000 | Neurologists’opinion |
| **Administration - Alemtuzumab** | | | | | | | | |
| 261 | Posology_Alemtuzumab_given_fail_naïve_2_7_year_Teriflunomide | Parameters (L678) | Posology (milligrams *per* administration) Alemtuzumab treatment given poor response to Teriflunomide naïve patients years 2_7 | 5 | 12.000 | 12.000 | 12.000 | Neurologists’opinion |
| 262 | Vol_administrations_Alemtuzumab_given_fail_naïve_2nd_year_Teriflunomide | Parameters (L679) | Number of administrations *per* year Alemtuzumab treatment given poor response to Teriflunomide naïve patients 2^nd^ year | 5 | 5.000 | 5.000 | 5.000 | Neurologists’opinion |
| 263 | Vol_administrations_Alemtuzumab_given_fail_naïve_3_7_year_Teriflunomide | Parameters (L680) | Number of administrations *per* year Alemtuzumab treatment given poor response to Teriflunomide naïve patients years 3_7 | 5 | 3.000 | 3.000 | 3.000 | Neurologists’opinion |
| **Administration disposables - Alemtuzumab** | | | | | | | | |
| 264 | Vol_adm_iv_line_Alemtuzumab_given_fail_naïve_2_7_year_Teriflunomide | Parameters (L682) | Number of iv lines for Alemtuzumab administration given poor response to Teriflunomide naïve patients years 2_7 | 5 | 1.000 | 1.000 | 1.000 | Neurologists’opinion |
| 265 | Vol_adm_needle_Alemtuzumab_given_fail_naïve_2_7_year_Teriflunomide | Parameters (L683) | Number of needles for Alemtuzumab administration given poor response to Teriflunomide naïve patients years 2_7 | 5 | 1.000 | 1.000 | 1.000 | Neurologists’opinion |
| 266 | Vol_adm_antiseptic_cc_Alemtuzumab_given_fail_naïve_2_7_year_Teriflunomide | Parameters (L684) | Volume (cubic centimeters *per* administration) of antiseptic for Alemtuzumab administration given poor response to Teriflunomide naïve patients years 2_7 naïve patients years 2_7 | 5 | 10.000 | 10.000 | 10.000 | Neurologists’opinion |
| **Postadministration disposables - Alemtuzumab** | | | | | | | | |
| 267 | Vol_postad_physio_Alemtuzumab_given_fail_naïve_2_7_year_Teriflunomide | Parameters (L686) | Number of physiological solutions postadministration Alemtuzumab given poor response to Teriflunomide naïve patients years 2_7 | 5 | 1.000 | 1.000 | 1.000 | Neurologists’opinion |
| **Postadministration time - Alemtuzumab** | | | | | | | | |
| 268 | Vol_postad_time_nurse_Alemtuzumab_given_fail_naïve_2_7_year_Teriflunomide | Parameters (L688) | Number of nurse time (minutes) for administration Alemtuzumab given poor response to Teriflunomide naïve patients years 2_7 | 5 | 90.000 | 90.000 | 90.000 | Neurologists’opinion |

**Table SI20 – Materials and Methods - Parameters with no statistical distribution for base case analysis, OWSA and PSA**

| **Parameter number** | **Parameter**  **name** | **Spreadsheet**  **(point estimate cell)** | **Parameter**  **description** | **Observations** | **Point estimate** | **LL range** | **UL**  **range** | **Source** |
| --- | --- | --- | --- | --- | --- | --- | --- | --- |
| **RRMS naïve and experienced patients on Teriflunomide** | | | | | | | | |
| **Non-Healthcare resources - Volume** | | | | | | | | |
| **Patient and caregiver's time** | | | | | | | | |
| 269 | Vol_min_adm_patient_Ocrelizumab_given_fail_naïve_2_7_year_Teriflunomide | Parameters (L703) | Number minutes patient time administration Ocrelizumab given poor response to Teriflunomide naïve patients years 2_7 | 6 | 210.000 | 210.000 | 210.000 | Neurologists’opinion |
| 270 | Vol_min_adm_patient_Alemtuzumab_given_fail_naïve_2_7_year_Teriflunomide | Parameters (L704) | Number minutes patient time administration Alemtuzumab given poor response to Teriflunomide naïve patients years 2_7 | 5 | 300.000 | 300.000 | 300.000 | Neurologists’opinion |
| **Discount rates** | | | | | | | | |
| 271 | disc_rate_cost | Parameters (L708) | Real social discount rate for costs for both naïve and experienced patients | 373 | 0.003 | 0.003 | 0.003 | [SI5.] |
| 272 | disc_rate_LYS_QALYs | Parameters (L709) | Real social discount rate for LYS and QALYs for both naïve and experienced patients | 373 | 0.003 | 0.003 | 0.003 | [SI5.] |
| **Healthcare resources - Unit and Yearly Costs** | | | | | | | | |
| **RRMS-related drugs** | | | | | | | | |
| 273 | uc_INHS_funded_Teriflunomide | Parameters (L792) | Unit cost (milligram) INHS-funded Teriflunomide for both naïve and experienced patients | 373 | €2.370 | €2.370 | €2.370 | [SI3.] |
| 274 | uc_INHS_funded_Natalizumab | Parameters (L793) | Unit cost (milligram) INHS-funded Natalizumab for naïve patients given poor response to Teriflunomide naïve patients years 2_7 | 15 | €5.420 | €5.420 | €5.420 | [SI3.] |
| 275 | uc_INHS_funded_Fingolimod | Parameters (L794) | Unit cost (milligram) INHS-funded Fingolimod for naïve patients given poor response to Teriflunomide naïve patients years 2_7 | 11 | €116.04 | €116.04 | €116.04 | [SI3.] |
| 276 | uc_INHS_funded_Cladribine | Parameters (L795) | Unit cost (milligram) INHS-funded Cladribine for naïve patients given poor response to Teriflunomide naïve patients years 2_7 | 7 | €191.87 | €191.87 | €191.87 | [SI3.] |
| 277 | uc_INHS_funded_Ocrelizumab | Parameters (L796) | Unit cost (milligram) INHS-funded Ocrelizumab for naïve patients given poor response to Teriflunomide naïve patients years 2_7 | 6 | €18.80 | €18.80 | €18.80 | [SI3.] |
| 278 | uc_INHS_funded_Alemtuzumab | Parameters (L797) | Unit cost (milligram) INHS-funded Alemtuzumab for naïve patients given poor response to Teriflunomide naïve patients years 2_7 | 5 | €662.77 | €662.77 | €662.77 | [SI3.] |
| **RRMS-related drugs - Relapse** | | | | | | | | |
| 279 | uc_INHS_funded_corticosteroids_given_RRMS_relapse_Teriflunomide | Parameters (L802) | Unit cost (gram) INHS-funded corticosteroid for RRMS relapse for both naïve and experienced patients | 373 | €35.54 | €35.54 | €35.54 | [SI3.] |
| **Non RRMS-related drugs - Premedication** | | | | | | | | |
| 280 | uc_INHS_funded_Corticosteroid_Natalizumab | Parameters (L804) | Unit cost (gram) INHS-funded corticosteroid for premedication Natalizumab for naïve patients given poor response to Teriflunomide naïve patients years 2_7 | 15 | €0.190 | €0.190 | €0.190 | [SI3.] |
| 281 | uc_INHS_funded_Corticosteroid_Ocrelizumab_Alemtuzumab | Parameters (L805) | Unit cost (gram) INHS-funded corticosteroid for prenedication Ocrelizumab and Alemtuzumab for naïve patients given poor response to Teriflunomide naïve patients years 2_7 | 11 | €35.54 | €35.54 | €35.54 | [SI3.] |
| 282 | uc_INHS_funded_Corticosteroid_Ocrelizumab_Alemtuzumab | Parameters (L805) | Unit cost (gram) INHS-funded corticosteroid for prenedication Ocrelizumab and Alemtuzumab for naïve patients given poor response to Teriflunomide naïve patients years 2_7 | 11 | €35.54 | €35.54 | €35.54 | [SI3.] |
| 283 | uc_INHS_funded_Antihistaminic_Ocrelizumab_Alemtuzumab | Parameters (L806) | Unit cost (milligram) INHS-funded antihistaminic for prenedication Ocrelizumab and Alemtuzumab for naïve patients given poor response to Teriflunomide naïve patients years 2_7 | 11 | €0.030 | €0.030 | €0.030 | [SI3.] |

**Table SI21 – Materials and Methods - Parameters with no statistical distribution for base case analysis, OWSA and PSA**

| **Parameter number** | **Parameter**  **name** | **Spreadsheet**  **(point estimate cell)** | **Parameter**  **description** | **Observations** | **Point estimate** | **LL**  **range** | **UL**  **range** | **Source** |
| --- | --- | --- | --- | --- | --- | --- | --- | --- |
| **RRMS naïve and experienced patients on Teriflunomide** | | | | | | | | |
| **Healthcare resources - Unit and Yearly Costs** | | | | | | | | |
| **Non RRMS-related drugs - Adverse events** | | | | | | | | |
| 284 | uc_OOP_funded_drug_rachialgy | Parameters (L818) | Unit cost (*per* table) OOP-funded drug for rachialgy for both naïve and experienced patients | 373 | €0.440 | €0.440 | €0.440 | [SI3.] |
| 285 | uc_INHS_funded_drug_hypertension | Parameters (L819) | Unit cost (*per* table) INHS-funded drug for hypertension for both naïve and experienced patients | 373 | €0.410 | €0.410 | €0.410 | [SI3.] |
| 286 | uc_INHS_funded_drug_haepatoprotection | Parameters (L820) | Unit cost (*per* table) INHS-funded drug for haepatoprotection for both naïve and experienced patients | 373 | €0.410 | €0.410 | €0.410 | [SI3.] |
| 287 | uc_INHS_funded_drug_minor_depression | Parameters (L821) | Unit cost (*per* table) INHS-funded drug for minor depression for both naïve and experienced patients | 373 | €0.200 | €0.200 | €0.200 | [SI3.] |
| 288 | uc_INHS_funded_drug_infection | Parameters (L822) | Unit cost (*per* table) INHS-funded drug for infection for both naïve and experienced patients | 373 | €1.250 | €1.250 | €1.250 | [SI3.] |

**Table SI22 – Materials and Methods - Parameters with statistical distribution for base case analysis, OWSA and PSA**

|  |  | |  | |  |  |  |  |  |  | **Parameters Beta distribution for PSA^a^** | |  |
| --- | --- | --- | --- | --- | --- | --- | --- | --- | --- | --- | --- | --- | --- |
| **Parameter number** | **Parameter**  **name** | **Spreadsheet**  **(point estimate cell)** | | **Parameter**  **description** | | **Observations** | **Mean** | **SE** | **LL 95% CI** | **UL 95% CI** | **Alfa** | **Beta** | **Source** |
| **RRMS naïve and experienced patients on Teriflunomide** | | | | | | | | | | | | | |
| **Anagraphics and demographics – Probability - Gender** | | | | | | | | | | | | | |
| 289 | Pr_Female_ naïve | Parameters  (L 27) | | Probability women among naïve RRMS patients on Teriflunomide | | 153 | 0.787 | 0.033 | 0. 719 | 0.848 | 120 | 33 | Neurologists’ opinion |
| 290 | Pr_Female_ exp | Parameters  (L 28) | | Probability women among experienced RRMS patients on Teriflunomide | | 220 | 0.771 | 0.028 | 0. 713 | 0.824 | 170 | 50 | Neurologists’ opinion |
| **Anagraphics and demographics – Probability - Employment and caregiving** | | | | | | | | | | | | | |
| 291 | Pr_Empl_naïve_1st_year | Parameters (L30) | | Probability employed among naïve RRMS patients on Teriflunomide 1st year | | 153 | 0.446 | 0.040 | 0.368 | 0.525 | 68 | 85 | Neurologists’ opinion |
| 292 | Pr_Empl_exp_1st_year | Parameters (L31) | | Probability employed among experienced RRMS patients on Teriflunomide 1st year | | 220 | 0.397 | 0.033 | 0.333 | 0.462 | 87 | 133 | Neurologists’ opinion |
| 293 | Pr_Empl_Red_naïve_2_7_years | Parameters  (L32) | | Probability of employment reduction among naïve RRMS patients on Teriflunomide years 2-7 | | 153 | 0.063 | 0.020 | 0.030 | 0.106 | 10 | 143 | Neurologists’ opinion |
| 294 | Pr_Empl_Red_exp_2_7_years | Parameters  (L33) | | Probability of employment reduction among experienced RRMS patients on Teriflunomide years 2-7 | | 220 | 0.033 | 0.012 | 0.013 | 0.060 | 7 | 213 | Neurologists’ opinion |
| 295 | Pr_Housekeepers_naïve_1st_year | Parameters (L34) | | Probability housekeeper among naïve RRMS patients on Teriflunomide 1st year | | 85 | 0.239 | 0.046 | 0.155 | 0.335 | 20 | 65 | Neurologists’ opinion |
| 296 | Pr_Housekeepers_exp_1st_year | Parameters (L35) | | Probability housekeeper among experienced RRMS patients on Teriflunomide 1st year | | 133 | 0.238 | 0.037 | 0.170 | 0.313 | 32 | 101 | Neurologists’ opinion |
| 297 | Pr_Housekeepers_Red_naïve_2_7_years | Parameters  (L36) | | Probability of housekeeper reduction among naïve RRMS patients on Teriflunomide years 2-7 | | 85 | 0.060 | 0.026 | 0.020 | 0.119 | 5 | 80 | Neurologists’ opinion |
| 298 | Pr_Housekeepers_Red_ exp _2_7_years | Parameters  (L37) | | Probability of housekeeper reduction among experienced RRMS patients on Teriflunomide years 2-7 | | 133 | 0.063 | 0.021 | 0.029 | 0.110 | 8 | 124 | Neurologists’ opinion |
| 299 | Pr_Care_giving_naïve_1st_year | Parameters (L38) | | Probability needing a caregiver among naïve RRMS patients on Teriflunomide 1st year | | 153 | 0.063 | 0.020 | 0.030 | 0.106 | 10 | 143 | Neurologists’ opinion |
| 300 | Pr_Care_giving_exp_1st_year | Parameters (L39) | | Probability needing a caregiver among experienced RRMS patients on Teriflunomide 1st year | | 220 | 0.150 | 0.024 | 0.106 | 0.200 | 33 | 187 | Neurologists’ opinion |
| 301 | Pr_Care_giving_naïve_2_7_year | Parameters (L40) | | Probability needing a caregiver among naïve RRMS patients on Teriflunomide years 2-7 | | 153 | 0.100 | 0.024 | 0.058 | 0.152 | 15 | 138 | Neurologists’ opinion |
| 302 | Pr_Care_giving_exp_2_7_year | Parameters (L41) | | Probability needing a caregiver among experienced RRMS patients on Teriflunomide years 2-7 | | 220 | 0.238 | 0.029 | 0.184 | 0.296 | 52 | 168 | Neurologists’ opinion |

^a^ Beta distribution was fitted to binomial data (e.g., parameters that encompass two categories, such as needing informal care or not), utility and disutility values ([SI1.]).

**Table SI23 – Materials and Methods - Parameters with statistical distribution for base case analysis, OWSA and PSA**

|  |  |  |  |  |  |  |  |  | **Parameters Beta distribution for PSA^a^** | |  |
| --- | --- | --- | --- | --- | --- | --- | --- | --- | --- | --- | --- |
| **Parameter number** | **Parameter**  **name** | **Spreadsheet**  **(point estimate cell)** | **Parameter**  **description** | **Observations** | **Mean** | **SE** | **LL 95% CI** | **UL 95% CI** | **Alfa** | **Beta** | **Source** |
| **RRMS naïve and experienced patients on Teriflunomide** | | | | | | | | | | | |
| **Healthcare resources – Probability - Disability aids** | | | | | | | | | | | |
| 303 | Pr_Disability_aids_naïve_1st_year | Parameters (L43) | Probability disability aids among naïve RRMS patients on Teriflunomide 1st year | 153 | 0.134 | 0.028 | 0.085 | 0.192 | 21 | 132 | [SI6.] |
| 304 | Pr_Disability_aids_exp_1st_year | Parameters (L44) | Probability disability aids among experienced RRMS patients on Teriflunomide 1st year | 220 | 0.134 | 0.023 | 0.092 | 0.182 | 29 | 191 | [SI6.] |
| 305 | Pr_Disability_aids_naïve_2nd_year | Parameters (L45) | Probability disability aids among naïve RRMS patients on Teriflunomide 2nd year | 153 | 0.134 | 0.028 | 0.085 | 0.192 | 21 | 132 | [SI6.] |
| 306 | Pr_Disability_aids_exp_2nd_year | Parameters (L46) | Probability disability aids among experienced RRMS patients on Teriflunomide 2nd year | 220 | 0.260 | 0.030 | 0.204 | 0.320 | 57 | 163 | [SI6.] |
| 307 | Pr_Disability_aids_naïve_3rd_year | Parameters (L47) | Probability disability aids among naïve RRMS patients on Teriflunomide 3rd year | 153 | 0.134 | 0.028 | 0.085 | 0.192 | 21 | 132 | [SI6.] |
| 308 | Pr_Disability_aids_exp_3rd_year | Parameters (L48) | Probability disability aids among experienced RRMS patients on Teriflunomide 3rd year | 220 | 0.260 | 0.030 | 0.204 | 0.320 | 57 | 163 | [SI6.] |
| 309 | Pr_Disability_aids_naïve_4th_year | Parameters (L49) | Probability disability aids among naïve RRMS patients on Teriflunomide 4th year | 153 | 0.134 | 0.028 | 0.085 | 0.192 | 21 | 132 | [SI6.] |
| 310 | Pr_Disability_aids_exp_4th _year | Parameters (L50) | Probability disability aids among experienced RRMS patients on Teriflunomide 4th year | 220 | 0.260 | 0.030 | 0.204 | 0.320 | 57 | 163 | [SI6.] |
| 311 | Pr_Disability_aids_naïve_5th_year | Parameters (L51) | Probability disability aids among naïve RRMS patients on Teriflunomide 5th year | 153 | 0.134 | 0.028 | 0.085 | 0.192 | 21 | 132 | [SI6.] |
| 312 | Pr_Disability_aids_exp_5th _year | Parameters (L52) | Probability disability aids among experienced RRMS patients on Teriflunomide 5^t^h year | 220 | 0.260 | 0.030 | 0.204 | 0.320 | 57 | 163 | [SI6.] |
| 313 | Pr_Disability_aids_naïve_6th_year | Parameters (L53) | Probability disability aids among naïve RRMS patients on Teriflunomide 6th year | 153 | 0.134 | 0.028 | 0.085 | 0.192 | 21 | 132 | [SI6.] |
| 314 | Pr_Disability_aids_exp_6th _year | Parameters (L54) | Probability disability aids among experienced RRMS patients on Teriflunomide 6th year | 220 | 0.404 | 0.033 | 0.340 | 0.469 | 89 | 131 | [SI6.] |
| 315 | Pr_Disability_aids_naïve_7th_year | Parameters (L55) | Probability disability aids among naïve RRMS patients on Teriflunomide 7th year | 153 | 0.260 | 0.035 | 0.194 | 0.332 | 40 | 113 | [SI6.] |
| 316 | Pr_Disability_aids_exp_7th _year | Parameters (L56) | Probability disability aids among experienced RRMS patients on Teriflunomide 7th year | 220 | 0.494 | 0.034 | 0.428 | 0.560 | 109 | 111 | [SI6.] |

^a^ Beta distribution was fitted to binomial data (eg, parameters that encompass two categories, such as needing disability aids among naïve RRMS patients on Teriflunomide 1st year or not) ([SM1.]).

**Table SI24 – Materials and Methods - Parameters with statistical distribution for base case analysis, OWSA and PSA**

|  |  |  |  |  |  |  |  |  | **Parameters Beta distribution for PSA^a^** | |  |
| --- | --- | --- | --- | --- | --- | --- | --- | --- | --- | --- | --- |
| **Parameter number** | **Parameter**  **name** | **Spreadsheet**  **(point estimate cell)** | **Parameter**  **description** | **Observations** | **Mean** | **SE** | **LL 95%**  **CI** | **UL 95%**  **CI** | **Alfa** | **Beta** | **Source** |
| **RRMS naïve and experienced patients on Teriflunomide** | | | | | | | | | | | |
| **Healthcare resources – Probability - Teriflunomide pre-treatment assessment** | | | | | | | | | | | |
| 317 | Pr_Lympho_subpop_test_pre_Teriflunomide | Parameters (L60) | Probability lymphocyte subpopulation test for both naïve and experienced RRMS patients before Teriflunomide treatment | 373 | 0.250 | 0.022 | 0.207 | 0.295 | 93 | 280 | Neurologists’ opinion |
| 318 | Pr_Neurologist_visit_pre_Teriflunomide | Parameters (L61) | Probability neurologist visit for both naïve and experienced RRMS patients before Teriflunomide treatment | 373 | 0.250 | 0.022 | 0.207 | 0.295 | 93 | 280 | Neurologists’ opinion |
| 319 | Pr_Dermatologist_visit_pre_Teriflunomide | Parameters (L62) | Probability dermatologist visit for both naïve and experienced RRMS patients before Teriflunomide treatment | 373 | 0.250 | 0.022 | 0.207 | 0.295 | 93 | 280 | Neurologists’ opinion |
| 320 | Pr_Cardiologist_visit_pre_Teriflunomide | Parameters (L63) | Probability cardiologist visit for both naïve and experienced RRMS patients before Teriflunomide treatment | 373 | 0.250 | 0.022 | 0.207 | 0.295 | 93 | 280 | Neurologists’ opinion |
| 321 | Pr_ECG_test_pre_Teriflunomide | Parameters (L64) | Probability ECG test for both naïve and experienced RRMS patients before Teriflunomide treatment | 373 | 0.250 | 0.022 | 0.207 | 0.295 | 93 | 280 | Neurologists’ opinion |
| 322 | Pr_Brain_MRI_pre_Teriflunomide | Parameters (L65) | Probability brain MRI for both naïve and experienced RRMS patients before Teriflunomide treatment | 373 | 0.250 | 0.022 | 0.207 | 0.295 | 93 | 280 | Neurologists’ opinion |
| 323 | Pr_Haepatic_test_Total_bilirubin_pre_Teriflunomide | Parameters (L69) | Probability total bilirubin test for both naïve and experienced RRMS patients before Teriflunomide treatment | 373 | 0.750 | 0.022 | 0.705 | 0.793 | 280 | 93 | Neurologists’ opinion |
| 324 | Pr_Haepatic_test_Direct_bilirubin_pre_Teriflunomide | Parameters (L71) | Probability direct bilirubin test for both naïve and experienced RRMS patients before Teriflunomide treatment | 373 | 0.250 | 0.022 | 0.207 | 0.295 | 93 | 280 | Neurologists’ opinion |
| 325 | Pr_Haepatic_test_Indirect_bilirubin_pre_Teriflunomide | Parameters (L72) | Probability indirect bilirubin test for both naïve and experienced RRMS patients before Teriflunomide treatment | 373 | 0.250 | 0.022 | 0.207 | 0.295 | 93 | 280 | Neurologists’ opinion |
| 326 | Pr_Haepatic_test_Alkaline_phosphatase_pre_Teriflunomide | Parameters (L73) | Probability alkaline_phosphatase test for both naïve and experienced RRMS patients before Teriflunomide treatment | 373 | 0.500 | 0.026 | 0.449 | 0.551 | 187 | 187 | Neurologists’ opinion |
| 327 | Pr_Sierology_test_HBV_pre_Teriflunomide | Parameters (L74) | Probability HBV antibodies test for both naïve and experienced RRMS patients before Teriflunomide treatment | 373 | 0.500 | 0.026 | 0.449 | 0.551 | 187 | 187 | Neurologists’ opinion |
| 328 | Pr_Sierology_test_HCV_pre_Teriflunomide | Parameters (L75) | Probability HCV antibodies test for both naïve and experienced RRMS patients before Teriflunomide treatment | 373 | 0.500 | 0.026 | 0.449 | 0.551 | 187 | 187 | Neurologists’ opinion |
| 329 | Pr_Sierology_test_HIV_pre_Teriflunomide | Parameters (L76) | Probability HIV antibodies test for both naïve and experienced RRMS patients before Teriflunomide treatment | 373 | 0.500 | 0.026 | 0.449 | 0.551 | 187 | 187 | Neurologists’ opinion |
| 330 | Pr_Sierology_test_zoster_pre_Teriflunomide | Parameters (L77) | Probability zooster virus test for both naïve and experienced RRMS patients before Teriflunomide treatment | 373 | 0.250 | 0.022 | 0.207 | 0.295 | 93 | 280 | Neurologists’ opinion |
| 331 | Pr_Kidney_test_Urea_pre_Teriflunomide | Parameters (L79) | Probability urea test for both naïve and experienced RRMS patients before Teriflunomide treatment | 373 | 0.500 | 0.026 | 0.449 | 0.551 | 187 | 187 | Neurologists’ opinion |
| 332 | Pr_Kidney_test_Urine_pre_Teriflunomide | Parameters (L80) | Probability urine test for both naïve and experienced RRMS patients before Teriflunomide treatment | 373 | 0.250 | 0.022 | 0.207 | 0.295 | 93 | 280 | Neurologists’ opinion |

^a^ Beta distribution was fitted to binomial data (e.g., parameters that encompass two categories, such as needing lymphocyte subpopulation test for both naïve and experienced RRMS patients before Teriflunomide treatment or not) ([SI1.]).

**Table SI25 – Materials and Methods - Parameters with statistical distribution for base case analysis, OWSA and PSA**

|  |  |  |  |  |  |  |  |  | **Parameters Beta distribution for PSA^a^** | |  |
| --- | --- | --- | --- | --- | --- | --- | --- | --- | --- | --- | --- |
| **Parameter number** | **Parameter**  **name** | **Spreadsheet**  **(point estimate cell)** | **Parameter**  **description** | **Observations** | **Mean** | **SE** | **LL 95% CI** | **UL 95% CI** | **Alfa** | **Beta** | **Source** |
| **RRMS naïve and experienced patients on Teriflunomide** | | | | | | | | | | | |
| **Healthcare resources – Probability - Teriflunomide liver monitoring** | | | | | | | | | | | |
| 333 | Pr_Haepatic_test_AST_monitoring_1st_year_Teriflunomide | Parameters (L84) | Probability AST test for both naïve and experienced RRMS patients for liver monitoring during Teriflunomide treatment 1st year | 373 | 0.750 | 0.022 | 0.705 | 0.793 | 280 | 93 | Neurologists’ opinion |
| 334 | Pr_Haepatic_test_gGT_monitoring_1st_year_Teriflunomide | Parameters (L85) | Probability gGT test for both naïve and experienced RRMS patients for liver monitoring during Teriflunomide treatment 1st year | 373 | 0.750 | 0.022 | 0.705 | 0.793 | 280 | 93 | Neurologists’ opinion |
| 335 | Pr_Haepatic_test_Total_bilirubin_monitoring_1st_year_Teriflunomide | Parameters (L86) | Probability total bilirubin test for both naïve and experienced RRMS patients for liver monitoring during Teriflunomide treatment 1st year | 373 | 0.250 | 0.022 | 0.207 | 0.295 | 93 | 280 | Neurologists’ opinion |
| 336 | Pr_Haepatic_test_Alkaline_phosphatase_monitoring_1st_year_Teriflunomide | Parameters (L87) | Probability alkaline phosphatase test for both naïve and experienced RRMS patients for liver monitoring during Teriflunomide treatment 1st year | 373 | 0.250 | 0.022 | 0.207 | 0.295 | 93 | 280 | Neurologists’ opinion |
| 337 | Pr_Haepatic_test_Total_bilirubin_monitoring_2_7_year_Teriflunomide | Parameters (L91) | Probability total bilirubin test for both naïve and experienced RRMS patients for liver monitoring during Teriflunomide treatment years 2-7 | 373 | 0.250 | 0.022 | 0.207 | 0.295 | 93 | 280 | Neurologists’ opinion |
| 338 | Pr_Haepatic_test_Alkaline_phosphatase_monitoring_2_7_year_Teriflunomide | Parameters (L92) | Probability alkaline phosphatase test for both naïve and experienced RRMS patients for liver monitoring during Teriflunomide treatment years 2-7 | 373 | 0.250 | 0.022 | 0.207 | 0.295 | 93 | 280 | Neurologists’ opinion |

^a^ Beta distribution was fitted to binomial data (e.g., parameters that encompass two categories, such as needing AST test for both naïve and experienced RRMS patients for liver monitoring during Teriflunomide treatment 1st year or not) ([SI1.]).

**Table SI26 – Materials and Methods - Parameters with statistical distribution for base case analysis, OWSA and PSA**

|  |  |  |  |  |  |  |  |  | **Parameters Beta distribution for PSA^a^** | |  |
| --- | --- | --- | --- | --- | --- | --- | --- | --- | --- | --- | --- |
| **Parameter number** | **Parameter**  **name** | **Spreadsheet**  **(point estimate cell)** | **Parameter**  **description** | **Observations** | **Mean** | **SE** | **LL 95% CI** | **UL 95% CI** | **Alfa** | **Beta** | **Source** |
| **RRMS naïve and experienced patients on Teriflunomide** | | | | | | | | | | | |
| **Healthcare resources – Probability - RRMS no relapse - Follow-up** | | | | | | | | | | | |
| 339 | Pr_Brain_MRI_NOC_fu_RRMS_NO_relapse_naïve_1st_year | Parameters (L95) | Probability brain MRI with no contrast for naïve RRMS patients for follow-up with no relapse during Teriflunomide treatment 1st year | 153 | 0.750 | 0.035 | 0.679 | 0.815 | 115 | 38 | Neurologists’ opinion |
| 340 | Pr_Brain_MRI_NOC_fu_RRMS_NO_relapse_exp_1st_year | Parameters (L96) | Probability brain MRI with no contrast for experienced RRMS patients for follow-up with no relapse during Teriflunomide treatment 1st year | 220 | 0.750 | 0.029 | 0.691 | 0.805 | 165 | 55 | Neurologists’ opinion |
| 341 | Pr_Brain_MRI_C_fu_RRMS_NO_relapse_naïve_1st_year | Parameters (L97) | Probability brain MRI with contrast for naïve RRMS patients for follow-up with no relapse during Teriflunomide treatment 1st year | 153 | 0.500 | 0.040 | 0.421 | 0.579 | 77 | 77 | Neurologists’ opinion |
| 342 | Pr_Brain_MRI_C_fu_RRMS_NO_relapse_exp_1st_year | Parameters (L98) | Probability brain MRI with contrast for experienced RRMS patients for follow-up with no relapse during Teriflunomide treatment 1st year | 220 | 0.500 | 0.034 | 0.434 | 0.566 | 110 | 110 | Neurologists’ opinion |
| 343 | Pr_Brain_plus_spinal_cord_MRI_fu_NO_RRMS_relapse_naïve_1st_year | Parameters (L99) | Probability brain plus spinal cord MRI with contrast for naïve RRMS patients for follow-up with no relapse during Teriflunomide treatment 1st year | 153 | 0.250 | 0.035 | 0.185 | 0.321 | 38 | 115 | Neurologists’ opinion |
| 344 | Pr_Brain_plus_spinal_cord_MRI_fu_NO_RRMS_relapse_exp_1st_year | Parameters (L100) | Probability brain plus spinal cord MRI with contrast for experienced RRMS patients for follow-up with no relapse during Teriflunomide treatment 1st year | 220 | 0.250 | 0.029 | 0.195 | 0.309 | 55 | 165 | Neurologists’ opinion |
| 345 | Pr_Blood_test_fu_RRMS_NO_relapse_naïve_1st_year | Parameters (L101) | Probability full blood test for naïve RRMS patients for follow-up with no relapse during Teriflunomide treatment 1st year | 153 | 0.250 | 0.035 | 0.185 | 0.321 | 38 | 115 | Neurologists’ opinion |
| 346 | Pr_Blood_test_fu_RRMS_NO_relapse_exp_1st_year | Parameters (L102) | Probability full blood test for experienced RRMS patients for follow-up with no relapse during Teriflunomide treatment 1st year | 220 | 0.250 | 0.029 | 0.195 | 0.309 | 55 | 165 | Neurologists’ opinion |
| 347 | Pr_Haepatic_test_ALT_fu_RRMS_NO_relapse_naïve_1st_year_Teriflunomide | Parameters (L103) | Probability ALT test for naïve RRMS patients for follow-up with no relapse during Teriflunomide treatment 1st year | 153 | 0.250 | 0.035 | 0.185 | 0.321 | 38 | 115 | Neurologists’ opinion |
| 348 | Pr_Haepatic_test_ALT_fu_RRMS_NO_relapse_exp_1st_year_Teriflunomide | Parameters (L104) | Probability ALT test for experienced RRMS patients for follow-up with no relapse during Teriflunomide treatment 1st year | 220 | 0.250 | 0.029 | 0.195 | 0.309 | 55 | 165 | Neurologists’ opinion |
| 349 | Pr_Haepatic_test_AST_fu_RRMS_NO_relapse_naïve_1st_year_Teriflunomide | Parameters (L105) | Probability AST test for naïve RRMS patients for follow-up with no relapse during Teriflunomide treatment 1st year | 153 | 0.250 | 0.035 | 0.185 | 0.321 | 38 | 115 | Neurologists’ opinion |
| 350 | Pr_Haepatic_test_AST_fu_RRMS_NO_relapse_exp_1st_year_Teriflunomide | Parameters (L106) | Probability AST test for experienced RRMS patients for follow-up with no relapse during Teriflunomide treatment 1st year | 220 | 0.250 | 0.029 | 0.195 | 0.309 | 55 | 165 | Neurologists’ opinion |

^a^ Beta distribution was fitted to binomial data (e.g., parameters that encompass two categories, such as needing brain MRI with no contrast for naïve RRMS patients for follow-up with no relapse during Teriflunomide treatment 1st year or not) ([SI1.]).

**Table SI27 – Materials and Methods - Parameters with statistical distribution for base case analysis, OWSA and PSA**

|  |  |  |  |  |  |  |  |  | **Parameters Beta distribution for PSA^a^** | |  |
| --- | --- | --- | --- | --- | --- | --- | --- | --- | --- | --- | --- |
| **Parameter number** | **Parameter**  **name** | **Spreadsheet**  **(point estimate cell)** | **Parameter**  **description** | **Observations** | **Mean** | **SE** | **LL 95% CI** | **UL 95% CI** | **Alfa** | **Beta** | **Source** |
| **RRMS naïve and experienced patients on Teriflunomide** | | | | | | | | | | | |
| **Healthcare resources – Probability - RRMS no relapse - Follow-up** | | | | | | | | | | | |
| 351 | Pr_Haepatic_test_gGT_fu_RRMS_NO_relapse_naïve_1st_year_Teriflunomide | Parameters (L107) | Probability gGT test for naïve RRMS patients for follow-up with no relapse during Teriflunomide treatment 1st year | 153 | 0.250 | 0.035 | 0.185 | 0.321 | 38 | 115 | Neurologists’ opinion |
| 352 | Pr_Haepatic_test_gGT_fu_RRMS_NO_relapse_exp_1st_year_Teriflunomide | Parameters (L108) | Probability gGT test for experienced RRMS patients for follow-up with no relapse during Teriflunomide treatment 1st year | 220 | 0.250 | 0.029 | 0.195 | 0.309 | 55 | 165 | Neurologists’ opinion |
| 353 | Pr_Haepatic_test_Alkaline_Phosphatase_fu_RRMS_NO_relapse_naïve_1st_year_Teriflunomide | Parameters (L109) | Probability alkaline_phosphatase test for naïve RRMS patients for follow-up with no relapse during Teriflunomide treatment 1st year | 153 | 0.250 | 0.035 | 0.185 | 0.321 | 38 | 115 | Neurologists’ opinion |
| 354 | Pr_Haepatic_test_Alkaline_Phosphatase_fu_RRMS_NO_relapse_exp_1st_year_Teriflunomide | Parameters (L110) | Probability alkaline_phosphatase test for experienced RRMS patients for follow-up with no relapse during Teriflunomide treatment 1st year | 220 | 0.250 | 0.029 | 0.195 | 0.309 | 55 | 165 | Neurologists’ opinion |
| 355 | Pr_Haepatic_test_Total_bilirubin_fu_RRMS_NO_relapse_naïve_1st_year_Teriflunomide | Parameters (L111) | Probability total bilirubine test for naïve RRMS patients for follow-up with no relapse during Teriflunomide treatment 1st year | 153 | 0.250 | 0.035 | 0.185 | 0.321 | 38 | 115 | Neurologists’ opinion |
| 356 | Pr_Haepatic_test_Total_bilirubin_fu_RRMS_NO_relapse_exp_1st_year_Teriflunomide | Parameters (L112) | Probability total bilirubine test for experienced RRMS patients for follow-up with no relapse during Teriflunomide treatment 1st year | 220 | 0.250 | 0.029 | 0.195 | 0.309 | 55 | 165 | Neurologists’ opinion |
| 357 | Pr_Kidney_test_Creatinine_fu_RRMS_NO_relapse_naïve_1st_year_Teriflunomide | Parameters (L113) | Probability creatinine test for naïve RRMS patients for follow-up with no relapse during Teriflunomide treatment 1st year | 153 | 0.250 | 0.035 | 0.185 | 0.321 | 38 | 115 | Neurologists’ opinion |
| 358 | Pr_Kidney_test_Creatinine_fu_RRMS_NO_relapse_exp_1st_year_Teriflunomide | Parameters (L114) | Probability creatinine test for experienced RRMS patients for follow-up with no relapse during Teriflunomide treatment 1st year | 220 | 0.250 | 0.029 | 0.195 | 0.309 | 55 | 165 | Neurologists’ opinion |
| 359 | Pr_Sierology_test_HBV_fu_RRMS_NO_relapse_naïve_1st_year_Teriflunomide | Parameters (L115) | Probability HBV test for naïve RRMS patients for follow-up with no relapse during Teriflunomide treatment 1st year | 153 | 0.250 | 0.035 | 0.185 | 0.321 | 38 | 115 | Neurologists’ opinion |
| 360 | Pr_Sierology_test_HBV_fu_RRMS_NO_relapse_exp_1st_year_Teriflunomide | Parameters (L116) | Probability HBV test for exp RRMS patients for follow-up with no relapse during Teriflunomide treatment 1st year | 220 | 0.250 | 0.029 | 0.195 | 0.309 | 55 | 165 | Neurologists’ opinion |
| 361 | Pr_Sierology_test_HCV_fu_RRMS_NO_relapse_naïve_1st_year_Teriflunomide | Parameters (L117) | Probability HCV test for naïve RRMS patients for follow-up with no relapse during Teriflunomide treatment 1st year | 153 | 0.250 | 0.035 | 0.185 | 0.321 | 38 | 115 | Neurologists’ opinion |
| 362 | Pr_Sierology_test_HCV_fu_RRMS_NO_relapse_exp_1st_year_Teriflunomide | Parameters (L118) | Probability HCV test for experienced RRMS patients for follow-up with no relapse during Teriflunomide treatment 1st year | 220 | 0.250 | 0.029 | 0.195 | 0.309 | 55 | 165 | Neurologists’ opinion |
| 363 | Pr_Sierology_test_HIV_fu_RRMS_NO_relapse_naïve_1st_year_Teriflunomide | Parameters (L119) | Probability HIV test for naïve RRMS patients for follow-up with no relapse during Teriflunomide treatment 1st year | 153 | 0.250 | 0.035 | 0.185 | 0.321 | 38 | 115 | Neurologists’ opinion |
| 364 | Pr_Sierology_test_HIV_fu_RRMS_NO_relapse_exp_1st_year_Teriflunomide | Parameters (L120) | Probability HIV test for experienced RRMS patients for follow-up with no relapse during Teriflunomide treatment 1st year | 220 | 0.250 | 0.029 | 0.195 | 0.309 | 55 | 165 | Neurologists’ opinion |

^a^ Beta distribution was fitted to binomial data (e.g., parameters that encompass two categories, such as needing gGT test for naïve RRMS patients for follow-up with no relapse during Teriflunomide treatment 1st year or not) ([SI1.]).

**Table SI28 – Materials and Methods - Parameters with statistical distribution for base case analysis, OWSA and PSA**

|  |  |  |  |  |  |  |  |  | **Parameters Beta distribution for PSA^a^** | |  |
| --- | --- | --- | --- | --- | --- | --- | --- | --- | --- | --- | --- |
| **Parameter number** | **Parameter**  **name** | **Spreadsheet**  **(point estimate cell)** | **Parameter**  **description** | **Observations** | **Mean** | **SE** | **LL 95% CI** | **UL 95% CI** | **Alfa** | **Beta** | **Source** |
| **RRMS naïve and experienced patients on Teriflunomide** | | | | | | | | | | | |
| **Healthcare resources – Probability - RRMS no relapse - Follow-up** | | | | | | | | | | | |
| 365 | Pr_Sierology_test_quantiferon_fu_RRMS_NO_relapse_naïve_1st_year_Teriflunomide | Parameters (L121) | Probability quantiferon test for naïve RRMS patients for follow-up with no relapse during Teriflunomide treatment 1st year | 153 | 0.250 | 0.035 | 0.185 | 0.321 | 38 | 115 | Neurologists’ opinion |
| 366 | Pr_Sierology_test_quantiferon_fu_RRMS_NO_relapse_exp_1st_year_Teriflunomide | Parameters (L122) | Probability quantiferon test for experienced RRMS patients for follow-up with no relapse during Teriflunomide treatment 1st year | 220 | 0.250 | 0.029 | 0.195 | 0.309 | 55 | 165 | Neurologists’ opinion |
| 367 | Pr_Sierology_test_zooster_fu_RRMS_NO_relapse_naïve_1st_year_Teriflunomide | Parameters (L123) | Probability zooster test for naïve RRMS patients for follow-up with no relapse during Teriflunomide treatment 1st year | 153 | 0.250 | 0.035 | 0.185 | 0.321 | 38 | 115 | Neurologists’ opinion |
| 368 | Pr_Sierology_test_ zooster_fu_RRMS_NO_relapse_exp_1st_year_Teriflunomide | Parameters (L124) | Probability zooster test for experienced RRMS patients for follow-up with no relapse during Teriflunomide treatment 1st year | 220 | 0.250 | 0.029 | 0.195 | 0.309 | 55 | 165 | Neurologists’ opinion |
| 369 | Pr_Physiology_test_vitamin_D_fu_RRMS_NO_relapse_naïve_1st_year_Teriflunomide | Parameters (L125) | Probability vitamin D test for naïve RRMS patients for follow-up with no relapse during Teriflunomide treatment 1st year | 153 | 0.250 | 0.035 | 0.185 | 0.321 | 38 | 115 | Neurologists’ opinion |
| 370 | Pr_Physiology_test_vitamin_D_fu_RRMS_NO_relapse_exp_1st_year_Teriflunomide | Parameters (L126) | Probability vitamin D test for experienced RRMS patients for follow-up with no relapse during Teriflunomide treatment 1st year | 220 | 0.250 | 0.029 | 0.195 | 0.309 | 55 | 165 | Neurologists’ opinion |
| 371 | Pr_Dermatologist_visit_fu_RRMS_NO_relapse_naïve_1st_year_Teriflunomide | Parameters (L129) | Probability dermatologist visit for naïve RRMS patients for follow-up with no relapse during Teriflunomide treatment 1st year | 153 | 0.250 | 0.035 | 0.185 | 0.321 | 38 | 115 | Neurologists’ opinion |
| 372 | Pr_Dermatologist_visit_fu_RRMS_NO_relapse_exp_1st_year_Teriflunomide | Parameters (L130) | Probability dermatologist visit for experienced RRMS patients for follow-up with no relapse during Teriflunomide treatment 1st year | 220 | 0.250 | 0.029 | 0.195 | 0.309 | 55 | 165 | Neurologists’ opinion |
| 373 | Pr_Cardiologist_visit_fu_RRMS_NO_relapse_naïve_1st_year_Teriflunomide | Parameters (L131) | Probability cardiologist visit for naïve RRMS patients for follow-up with no relapse during Teriflunomide treatment 1st year | 153 | 0.250 | 0.035 | 0.185 | 0.321 | 38 | 115 | Neurologists’ opinion |
| 374 | Pr_Cardiologist_visit_fu_RRMS_NO_relapse_exp_1st_year_Teriflunomide | Parameters (L132) | Probability cardiologist visit for experienced RRMS patients for follow-up with no relapse during Teriflunomide treatment 1st year | 220 | 0.250 | 0.029 | 0.195 | 0.309 | 55 | 165 | Neurologists’ opinion |
| 375 | Pr_Brain_MRI_NOC_fu_RRMS_NO_relapse_naïve_2_7_year | Parameters (L133) | Probability brain MRI with no contrast for naïve RRMS patients for follow-up with no relapse during Teriflunomide treatment years 2-7 | 153 | 0.500 | 0.040 | 0.421 | 0.579 | 77 | 77 | Neurologists’ opinion |
| 376 | Pr_Brain_MRI_NOC_fu_RRMS_NO_relapse_exp_2_7_year | Parameters (L134) | Probability brain MRI with no contrast for experienced RRMS patients for follow-up with no relapse during Teriflunomide treatment years 2-7 | 220 | 0.500 | 0.034 | 0.434 | 0.566 | 110 | 110 | Neurologists’ opinion |
| 377 | Pr_Brain_MRI_C_fu_RRMS_NO_relapse_naïve_2_7_year | Parameters (L135) | Probability brain MRI with contrast for naïve RRMS patients for follow-up with no relapse during Teriflunomide treatment years 2-7 | 153 | 0.500 | 0.040 | 0.421 | 0.579 | 77 | 77 | Neurologists’ opinion |
| 378 | Pr_Brain_MRI_C_fu_RRMS_NO_relapse_experienced_2_7_year | Parameters (L136) | Probability brain MRI with contrast for experienced RRMS patients for follow-up with no relapse during Teriflunomide treatment years 2-7 | 220 | 0.250 | 0.029 | 0.195 | 0.309 | 55 | 165 | Neurologists’ opinion |

^a^ Beta distribution was fitted to binomial data (e.g., parameters that encompass two categories, such as needing quantiferon test for naïve RRMS patients for follow-up with no relapse during Teriflunomide treatment 1st year or not) ([SI1.]).

**Table SI29 – Materials and Methods - Parameters with statistical distribution for base case analysis, OWSA and PSA**

|  |  |  |  |  |  |  |  |  | **Parameters Beta distribution for PSA^a^** | |  |
| --- | --- | --- | --- | --- | --- | --- | --- | --- | --- | --- | --- |
| **Parameter number** | **Parameter**  **name** | **Spreadsheet**  **(point estimate cell)** | **Parameter**  **description** | **Observations** | **Mean** | **SE** | **LL 95% CI** | **UL 95% CI** | **Alfa** | **Beta** | **Source** |
| **RRMS naïve and experienced patients on Teriflunomide** | | | | | | | | | | | |
| **Healthcare resources – Probability - RRMS no relapse - Follow-up** | | | | | | | | | | | |
| 379 | Pr_Brain_plus_spinal_cord_MRI_fu_NO_RRMS_relaps_naïve_2_7_year | Parameters (L137) | Probability brain plus spinal cord MRI with contrast for naïve RRMS patients for follow-up with no relapse during Teriflunomide treatment years 2-7 | 153 | 0.250 | 0.035 | 0.185 | 0.321 | 38 | 115 | Neurologists’ opinion |
| 380 | Pr_Brain_plus_spinal_cord_MRI_fu_NO_RRMS_relaps_exp_2_7_year | Parameters (L138) | Probability brain plus spinal cord MRI with contrast for experienced RRMS patients for follow-up with no relapse during Teriflunomide treatment years 2-7 | 220 | 0.250 | 0.029 | 0.195 | 0.309 | 55 | 165 | Neurologists’ opinion |
| 381 | Pr_Blood_test_fu_RRMS_NO_relapse_naïve_2_7_year | Parameters (L139) | Probability full blood test for naïve RRMS patients for follow-up with no relapse during Teriflunomide treatment years 2-7 | 153 | 0.500 | 0.040 | 0.421 | 0.579 | 77 | 77 | Neurologists’ opinion |
| 382 | Pr_Blood_test_fu_RRMS_NO_relapse_exp_2_7_year | Parameters (L140) | Probability full blood test for experienced RRMS patients for follow-up with no relapse during Teriflunomide treatment years 2-7 | 220 | 0.500 | 0.034 | 0.434 | 0.566 | 110 | 110 | Neurologists’ opinion |
| 383 | Pr_Haepatic_test_ALT_fu_RRMS_NO_relapse_naïve_2_7_year_Teriflunomide | Parameters (L141) | Probability ALT test for naïve RRMS patients for follow-up with no relapse during Teriflunomide treatment years 2-7 | 153 | 0.500 | 0.040 | 0.421 | 0.579 | 77 | 77 | Neurologists’ opinion |
| 384 | Pr_Haepatic_test_ALT_fu_RRMS_NO_relapse_exp_2_7_year_Teriflunomide | Parameters (L142) | Probability ALT test for experienced RRMS patients for follow-up with no relapse during Teriflunomide treatment years 2-7 | 220 | 0.500 | 0.034 | 0.434 | 0.566 | 110 | 110 | Neurologists’ opinion |
| 385 | Pr_Haepatic_test_AST_fu_RRMS_NO_relapse_naïve_2_7_year_Teriflunomide | Parameters (L143) | Probability AST test for naïve RRMS patients for follow-up with no relapse during Teriflunomide treatment years 2-7 | 153 | 0.500 | 0.040 | 0.421 | 0.579 | 77 | 77 | Neurologists’ opinion |
| 386 | Pr_Haepatic_test_ALT_fu_RRMS_NO_relapse_exp_2_7_year_Teriflunomide | Parameters (L144) | Probability AST test for experienced RRMS patients for follow-up with no relapse during Teriflunomide treatment years 2-7 | 220 | 0.500 | 0.034 | 0.434 | 0.566 | 110 | 110 | Neurologists’ opinion |
| 387 | Pr_Haepatic_test_gGT_fu_RRMS_NO_relapse_naïve_2_7_year_Teriflunomide | Parameters (L145) | Probability gGT test for naïve RRMS patients for follow-up with no relapse during Teriflunomide treatment years 2-7 | 153 | 0.500 | 0.040 | 0.421 | 0.579 | 77 | 77 | Neurologists’ opinion |
| 388 | Pr_Haepatic_test_ gGT _fu_RRMS_NO_relapse_exp_2_7_year_Teriflunomide | Parameters (L146) | Probability gGT test for experienced RRMS patients for follow-up with no relapse during Teriflunomide treatment years 2-7 | 220 | 0.500 | 0.034 | 0.434 | 0.566 | 110 | 110 | Neurologists’ opinion |
| 389 | Pr_Haepatic_test_Alkaline_Phosphatase_fu_RRMS_NO_relapse_naïve_2_7_year_Teriflunomide | Parameters (L147) | Probability alkaline_phosphatase test for naïve RRMS patients for follow-up with no relapse during Teriflunomide treatment years 2-7 | 153 | 0.250 | 0.035 | 0.185 | 0.321 | 38 | 115 | Neurologists’ opinion |
| 390 | Pr_Haepatic_test_Alkaline_Phosphatase_fu_RRMS_NO_relapse_exp_2_7_year_Teriflunomide | Parameters (L148) | Probability alkaline_phosphatase test for experienced RRMS patients for follow-up with no relapse during Teriflunomide treatment years 2-7 | 220 | 0.250 | 0.029 | 0.195 | 0.309 | 55 | 165 | Neurologists’ opinion |
| 391 | Pr_Haepatic_test_Total_bilirubin_fu_RRMS_NO_relapse_naïve_2_7_year_Teriflunomide | Parameters (L149) | Probability total bilirubine test for naïve RRMS patients for follow-up with no relapse during Teriflunomide treatment years 2-7 | 153 | 0.250 | 0.035 | 0.185 | 0.321 | 38 | 115 | Neurologists’ opinion |
| 392 | Pr_Haepatic_test_Total_bilirubin_fu_RRMS_NO_relapse_exp_2_7_year_Teriflunomide | Parameters (L150) | Probability total bilirubine test for experienced RRMS patients for follow-up with no relapse during Teriflunomide treatment years 2-7 | 220 | 0.250 | 0.029 | 0.195 | 0.309 | 55 | 165 | Neurologists’ opinion |

^a^ Beta distribution was fitted to binomial data (e.g., parameters that encompass two categories, such as needing brain plus spinal cord MRI with contrast for naïve RRMS patients for follow-up with no relapse or not) ([SI1.]).

**Table SI30 – Materials and Methods - Parameters with statistical distribution for base case analysis, OWSA and PSA**

|  |  |  |  |  |  |  |  |  | **Parameters Beta distribution for PSA^a^** | |  |
| --- | --- | --- | --- | --- | --- | --- | --- | --- | --- | --- | --- |
| **Parameter number** | **Parameter**  **name** | **Spreadsheet**  **(point estimate cell)** | **Parameter**  **description** | **Observations** | **Mean** | **SE** | **LL**  **95%**  **CI** | **UL**  **95%**  **CI** | **Alfa** | **Beta** | **Source** |
| **RRMS naïve and experienced patients on Teriflunomide** | | | | | | | | | | | |
| **Healthcare resources – Probability - RRMS no relapse - Follow-up** | | | | | | | | | | | |
| 393 | Pr_Kidney_test_Creatinine_fu_RRMS_NO_relapse_naïve_2_7_year_Teriflunomide | Parameters (L151) | Probability creatinine test for naïve RRMS patients for follow-up with no relapse during Teriflunomide treatment years 2-7 | 153 | 0.250 | 0.035 | 0.185 | 0.321 | 38 | 115 | Neurologists’ opinion |
| 394 | Pr_Kidney_test_Creatinine_fu_RRMS_NO_relapse_exp_2_7_year_Teriflunomide | Parameters (L152) | Probability creatinine test for experienced RRMS patients for follow-up with no relapse during Teriflunomide treatment years 2-7 | 220 | 0.250 | 0.029 | 0.195 | 0.309 | 55 | 165 | Neurologists’ opinion |
| 395 | Pr_Sierology_test_HBV_fu_RRMS_NO_relapse_naïve_2_7_year_Teriflunomide | Parameters (L153) | Probability HBV test for naïve RRMS patients for follow-up with no relapse during Teriflunomide treatment years 2-7 | 153 | 0.250 | 0.035 | 0.185 | 0.321 | 38 | 115 | Neurologists’ opinion |
| 396 | Pr_Sierology_test_HBV_fu_RRMS_NO_relapse_exp__2_7_year_Teriflunomide | Parameters (L154) | Probability HBV test for exp RRMS patients for follow-up with no relapse during Teriflunomide treatment years 2-7 | 220 | 0.250 | 0.029 | 0.195 | 0.309 | 55 | 165 | Neurologists’ opinion |
| 397 | Pr_Sierology_test_HCV_fu_RRMS_NO_relapse_naïve_2_7_year_Teriflunomide | Parameters (L155) | Probability HCV test for naïve RRMS patients for follow-up with no relapse during Teriflunomide treatment years 2-7 | 153 | 0.250 | 0.035 | 0.185 | 0.321 | 38 | 115 | Neurologists’ opinion |
| 398 | Pr_Sierology_test_HCV_fu_RRMS_NO_relapse_exp__2_7_year_Teriflunomide | Parameters (L156) | Probability HCV test for exp RRMS patients for follow-up with no relapse during Teriflunomide treatment years 2-7 | 220 | 0.250 | 0.029 | 0.195 | 0.309 | 55 | 165 | Neurologists’ opinion |
| 399 | Pr_Sierology_test_HIV_fu_RRMS_NO_relapse_naïve_2_7_year_Teriflunomide | Parameters (L157) | Probability HIV test for naïve RRMS patients for follow-up with no relapse during Teriflunomide treatment years 2-7 | 153 | 0.250 | 0.035 | 0.185 | 0.321 | 38 | 115 | Neurologists’ opinion |
| 400 | Pr_Sierology_test_HIV_fu_RRMS_NO_relapse_exp__2_7_year_Teriflunomide | Parameters (L158) | Probability HIV test for exp RRMS patients for follow-up with no relapse during Teriflunomide treatment years 2-7 | 220 | 0.250 | 0.029 | 0.195 | 0.309 | 55 | 165 | Neurologists’ opinion |
| 401 | Pr_Sierology_test_quantiferon_fu_RRMS_NO_relapse_naïve_2_7_year_Teriflunomide | Parameters (L159) | Probability quantiferon test for naïve RRMS patients for follow-up with no relapse during Teriflunomide treatment years 2-7 | 153 | 0.250 | 0.035 | 0.185 | 0.321 | 38 | 115 | Neurologists’ opinion |
| 402 | Pr_Sierology_test_quantiferon_fu_RRMS_NO_relapse_exp_2_7_year_Teriflunomide | Parameters (L160) | Probability quantiferon test for experienced RRMS patients for follow-up with no relapse during Teriflunomide treatment years 2-7 | 220 | 0.250 | 0.029 | 0.195 | 0.309 | 55 | 165 | Neurologists’ opinion |
| 403 | Pr_Sierology_test_zooster_fu_RRMS_NO_relapse_naïve_2_7_year_Teriflunomide | Parameters (L161) | Probability zooster test for naïve RRMS patients for follow-up with no relapse during Teriflunomide treatment years 2-7 | 153 | 0.250 | 0.035 | 0.185 | 0.321 | 38 | 115 | Neurologists’ opinion |
| 404 | Pr_Sierology_test_ zooster _fu_RRMS_NO_relapse_exp_2_7_year_Teriflunomide | Parameters (L162) | Probability zooster test for experienced RRMS patients for follow-up with no relapse during Teriflunomide treatment years 2-7 | 220 | 0.250 | 0.029 | 0.195 | 0.309 | 55 | 165 | Neurologists’ opinion |
| 405 | Pr_Physiology_test_vitamin_D_fu_RRMS_NO_relapse_naïve_2_7_year_Teriflunomide | Parameters (L163) | Probability vitamin D test for naïve RRMS patients for follow-up with no relapse during Teriflunomide treatment years 2-7 | 153 | 0.250 | 0.035 | 0.185 | 0.321 | 38 | 115 | Neurologists’ opinion |
| 406 | Pr_Physiology_test_vitamin_D_fu_RRMS_NO_relapse_exp_2_7_year_Teriflunomide | Parameters (L164) | Probability vitamin D test for experienced RRMS patients for follow-up with no relapse during Teriflunomide treatment years 2-7 | 220 | 0.250 | 0.029 | 0.195 | 0.309 | 55 | 165 | Neurologists’ opinion |
| 407 | Pr_Dermatologist_visit_fu_RRMS_NO_relapse_naïve_2_7_year_Teriflunomide | Parameters (L167) | Probability dermatologist visit for naïve RRMS patients for follow-up with no relapse during Teriflunomide treatment years 2-7 | 153 | 0.250 | 0.035 | 0.185 | 0.321 | 38 | 115 | Neurologists’ opinion |
| 408 | Pr_Dermatologist_visit_fu_RRMS_NO_relapse_exp__2_7_year_year_Teriflunomide | Parameters (L168) | Probability dermatologist visit for experienced RRMS patients for follow-up with no relapse during Teriflunomide treatment years 2-7 | 220 | 0.250 | 0.029 | 0.195 | 0.309 | 55 | 165 | Neurologists’ opinion |
| 409 | Pr_Cardiologist_visit_fu_RRMS_NO_relapse_naïve_2_7_year_Teriflunomide | Parameters (L169) | Probability cardiologist visit for naïve RRMS patients for follow-up with no relapse during Teriflunomide treatment years 2-7 | 153 | 0.250 | 0.035 | 0.185 | 0.321 | 38 | 115 | Neurologists’ opinion |
| 410 | Pr_Cardiologist_visit_fu_RRMS_NO_relapse_exp_2_7_year_Teriflunomide | Parameters (L170) | Probability cardiologist visit for experienced RRMS patients for follow-up with no relapse during Teriflunomide treatment years 2-7 | 220 | 0.250 | 0.029 | 0.195 | 0.309 | 55 | 165 | Neurologists’ opinion |

^a^ Beta distribution was fitted to binomial data (e.g., parameters that encompass two categories, such as needing creatinine test for naïve RRMS patients for follow-up with no relapse or not) ([SI1.]).

**Table SI31 – Materials and Methods - Parameters with statistical distribution for base case analysis, OWSA and PSA**

|  |  |  |  |  |  |  |  |  | **Parameters Beta distribution for PSA^a^** | |  |
| --- | --- | --- | --- | --- | --- | --- | --- | --- | --- | --- | --- |
| **Parameter number** | **Parameter**  **name** | **Spreadsheet**  **(point estimate cell)** | **Parameter**  **description** | **Observations** | **Mean** | **SE** | **LL 95% CI** | **UL 95% CI** | **Alfa** | **Beta** | **Source** |
| **RRMS naïve and experienced patients on Teriflunomide** | | | | | | | | | | | |
| **Clinical events – Probability - RRMS relapse - Incidence** | | | | | | | | | | | |
| 411 | Pr_RRMS_relapse_naïve_1st_year | Parameters (L172) | Probability relapse for naïve RRMS patients 1st year | 153 | 0.460 | 0.040 | 0.382 | 0.539 | 70 | 83 | [SI7.] |
| 412 | Pr_RRMS_relapse_exp_1st_year | Parameters (L173) | Probability relapse for experienced RRMS patients 1st year | 220 | 0.495 | 0.034 | 0.429 | 0.561 | 109 | 111 | [SI7.] |
| 413 | Pr_RRMS_relapse_naïve_2nd_year | Parameters (L174) | Probability relapse for naïve RRMS patients 2nd year | 153 | 0.495 | 0.040 | 0.416 | 0.574 | 76 | 77 | [SI7.] |
| 414 | Pr_RRMS_relapse_exp_2nd_year | Parameters (L175) | Probability relapse for experienced RRMS patients 2nd year | 220 | 0.670 | 0.032 | 0.607 | 0.730 | 147 | 73 | [SI7.] |
| 415 | Pr_RRMS_relapse_naïve_3rd_year | Parameters (L176) | Probability relapse for naïve RRMS patients 3rd year | 153 | 0.495 | 0.040 | 0.416 | 0.574 | 76 | 77 | [SI7.] |
| 416 | Pr_RRMS_relapse_ exp _3rd_year | Parameters (L177) | Probability relapse for experienced RRMS patients 3rd year | 220 | 0.670 | 0.032 | 0.607 | 0.730 | 147 | 73 | [SI7.] |
| 417 | Pr_RRMS_relapse_naïve_4th_year | Parameters (L178) | Probability relapse for naïve RRMS patients 4th year | 153 | 0.495 | 0.040 | 0.416 | 0.574 | 76 | 77 | [SI7.] |
| 418 | Pr_RRMS_relapse_ exp _4th_year | Parameters (L179) | Probability relapse for experienced RRMS patients 4th year | 220 | 0.181 | 0.026 | 0.133 | 0.234 | 40 | 180 | [SI7.] |
| 419 | Pr_RRMS_relapse_naïve_5th_year | Parameters (L180) | Probability relapse for naïve RRMS patients 5th year | 153 | 0.495 | 0.040 | 0.416 | 0.574 | 76 | 77 | [SI7.] |
| 420 | Pr_RRMS_relapse_ exp _5th_year | Parameters (L181) | Probability relapse for experienced RRMS patients 5th year | 220 | 0.181 | 0.026 | 0.133 | 0.234 | 40 | 180 | [SI7.] |
| 421 | Pr_RRMS_relapse_naïve_6th_year | Parameters (L182) | Probability relapse for naïve RRMS patients 6^th^ year | 153 | 0.495 | 0.040 | 0.416 | 0.574 | 76 | 77 | [SI7.] |
| 422 | Pr_RRMS_relapse_ exp _6th_year | Parameters (L183) | Probability relapse for experienced RRMS patients 6th year | 220 | 0.150 | 0.024 | 0.106 | 0.200 | 33 | 187 | [SI7.] |
| 423 | Pr_RRMS_relapse_naïve_7th_year | Parameters (L184) | Probability relapse for naïve RRMS patients 7th year | 153 | 0.670 | 0.038 | 0.594 | 0.742 | 102 | 51 | [SI7.] |
| 424 | Pr_RRMS_relapse_ exp _7th_year | Parameters (L185) | Probability relapse for experienced RRMS patients 7th year | 220 | 0.156 | 0.024 | 0.111 | 0.206 | 34 | 186 | [SI7.] |
| **Clinical events – Probability - RRMS remitting after relapse - Incidence** | | | | | | | | | | | |
| 425 | Cond_pr_remitting_given_RRMS_relapse | Parameters (L187) | Probability remitting given relapse for both naïve and experienced RRMS patients | 373 | 0.200 | 0.021 | 0.161 | 0.242 | 75 | 298 | [SI8.] |

^a^ Beta distribution was fitted to binomial data (e.g., parameters that encompass two categories, such as remitting given relapse or not) ([SI1.]).

**Table SI32 – Materials and Methods - Parameters with statistical distribution for base case analysis, OWSA and PSA**

|  |  |  |  |  |  |  |  |  | **Parameters Beta distribution for PSA^a^** | |  |
| --- | --- | --- | --- | --- | --- | --- | --- | --- | --- | --- | --- |
| **Parameter number** | **Parameter**  **name** | **Spreadsheet**  **(point estimate cell)** | **Parameter**  **description** | **Observations** | **Mean** | **SE** | **LL 95%**  **CI** | **UL 95%**  **CI** | **Alfa** | **Beta** | **Source** |
| **RRMS naïve and experienced patients on Teriflunomide** | | | | | | | | | | | |
| **Healthcare resources – Probability - RRMS relapse - Management** | | | | | | | | | | | |
| 426 | Cond_pr_Corticosteroids_given_RRMS_relapse_1st_year_Teriflunomide | Parameters (L190) | Conditional probability corticosteroids given RRMS relapse for both naïve and experienced patients 1st year | 373 | 0.250 | 0.022 | 0.207 | 0.295 | 93 | 280 | Neurologists’ opinion |
| 427 | Cond_pr_Corticosteroids_DH_admin_given_RRMS_relapse_1st_year_Teriflunomide | Parameters (L191) | Conditional probability corticosteroids administration in DH given RRMS relapse for both naïve and experienced patients 1st year | 373 | 0.250 | 0.022 | 0.207 | 0.295 | 93 | 280 | Neurologists’ opinion |
| 428 | Cond_pr_Blood_test_given_RRMS_relapse_1st_yea_Teriflunomide | Parameters (L192) | Conditional probability full blood test given RRMS relapse for both naïve and experienced patients 1st year | 373 | 0.250 | 0.022 | 0.207 | 0.295 | 93 | 280 | Neurologists’ opinion |
| 429 | Cond_pr_Metabolic_test_blood_sugar_given_RRMS_relapse_1st_year_Teriflunomide | Parameters (L193) | Conditional probability blood sugar test given RRMS relapse for both naïve and experienced patients 1^s^t year | 373 | 0.250 | 0.022 | 0.207 | 0.295 | 93 | 280 | Neurologists’ opinion |
| 430 | Cond_pr_Kidney_test_Creatinine_given_RRMS_relapse_1st_year_Teriflunomide | Parameters (L194) | Conditional probability creatinine test given RRMS relapse for both naïve and experienced patients 1st year | 373 | 0.250 | 0.022 | 0.207 | 0.295 | 93 | 280 | Neurologists’ opinion |
| 431 | Cond_pr_Urine_test_Creatinine_given_RRMS_relapse_1st_year_Teriflunomide | Parameters (L195) | Conditional probability urine test given RRMS relapse for both naïve and experienced patients 1st year | 373 | 0.250 | 0.022 | 0.207 | 0.295 | 93 | 280 | Neurologists’ opinion |
| 432 | Cond_pr_Haepatic_test_ALT_given_RRMS_relapse_1st_year_Teriflunomide | Parameters (L196) | Conditional probability ALT test given RRMS relapse for both naïve and experienced patients 1st year | 373 | 0.250 | 0.022 | 0.207 | 0.295 | 93 | 280 | Neurologists’ opinion |
| 433 | Cond_pr_Haepatic_test_AST_given_RRMS_relapse_1st_year_Teriflunomide | Parameters (L197) | Conditional probability AST test given RRMS relapse for both naïve and experienced patients 1st year | 373 | 0.250 | 0.022 | 0.207 | 0.295 | 93 | 280 | Neurologists’ opinion |
| 434 | Cond_pr_Haepatic_test_gGT_given_RRMS_relapse_1st_year_Teriflunomide | Parameters (L198) | Conditional probability gGT test given RRMS relapse for both naïve and experienced patients 1st year | 373 | 0.250 | 0.022 | 0.207 | 0.295 | 93 | 280 | Neurologists’ opinion |
| 435 | Cond_pr_Brain_MRI_C_given_RRMS_relapse_1st_year_Teriflunomide | Parameters (L199) | Conditional probability brain MRI with contrast given RRMS relapse for both naïve and experienced patients 1st year | 373 | 0.125 | 0.017 | 0.093 | 0.160 | 47 | 236 | Neurologists’ opinion |
| 436 | Cond_pr_Brain_plus_spinal_cord_MRI_C_given_RRMS_relapse_1st_year_Teriflunomide | Parameters (L200) | Conditional probability brain plus spinal cord MRI with contrast given RRMS relapse for both naïve and experienced patients 1st year | 373 | 0.175 | 0.020 | 0.138 | 0.215 | 65 | 308 | Neurologists’ opinion |
| 437 | Cond_pr_Corticosteroids_given_RRMS_relapse_2_7_year_Teriflunomide | Parameters (L202) | Conditional probability corticosteroids given RRMS relapse for both naïve and experienced patients years 2-7 | 373 | 0.988 | 0.006 | 0.974 | 0.996 | 368 | 5 | Neurologists’ opinion |
| 438 | Cond_pr_Corticosteroids_DH_admin_given_RRMS_relapse_2_7_year _Teriflunomide | Parameters (L203) | Conditional probability corticosteroids administration in DH given RRMS relapse for both naïve and experienced patients years 2-7 | 373 | 0.250 | 0.022 | 0.207 | 0.295 | 93 | 280 | Neurologists’ opinion |
| 439 | Cond_pr_Blood_test_given_RRMS_relapse_2_7_year_Teriflunomide | Parameters (L204) | Conditional probability full blood test given RRMS relapse for both naïve and experienced patients years 2-7 | 373 | 0.250 | 0.022 | 0.207 | 0.295 | 93 | 280 | Neurologists’ opinion |
| 440 | Cond_pr_Metabolic_test_blood_sugar_given_RRMS_relapse_2_7_year_Teriflunomide | Parameters (L205) | Conditional probability blood sugar test given RRMS relapse for both naïve and experienced patients years 2-7 | 373 | 0.250 | 0.022 | 0.207 | 0.295 | 93 | 280 | Neurologists’ opinion |
| 441 | Cond_pr_Kidney_test_Creatinine_given_RRMS_relapse_2_7_year_Teriflunomide | Parameters (L206) | Conditional probability creatinine test given RRMS relapse for both naïve and experienced patients years 2-7 | 373 | 0.250 | 0.022 | 0.207 | 0.295 | 93 | 280 | Neurologists’ opinion |
| 442 | Cond_pr_Kidney_test_Urine_given_RRMS_relapse_2_7_year_Teriflunomide | Parameters (L207) | Conditional probability urine test given RRMS relapse for both naïve and experienced patients years 2-7 | 373 | 0.250 | 0.022 | 0.207 | 0.295 | 93 | 280 | Neurologists’ opinion |

^a^ Beta distribution was fitted to binomial data (e.g., parameters that encompass two categories, such as needing corticosteroids given RRMS relapse or not) ([SI1.]).

**Table SI33 – Materials and Methods - Parameters with statistical distribution for base case analysis, OWSA and PSA**

|  |  |  |  |  |  |  |  |  | **Parameters Beta distribution for PSA^a^** | |  |
| --- | --- | --- | --- | --- | --- | --- | --- | --- | --- | --- | --- |
| **Parameter number** | **Parameter**  **name** | **Spreadsheet**  **(point estimate cell)** | **Parameter**  **description** | **Observations** | **Mean** | **SE** | **LL**  **95%**  **CI** | **UL**  **95%**  **CI** | **Alfa** | **Beta** | **Source** |
| **RRMS naïve and experienced patients on Teriflunomide** | | | | | | | | | | | |
| **Healthcare resources – Probability - RRMS relapse - Management** | | | | | | | | | | | |
| 443 | Cond_pr_Haepatic_test_ALT_given_RRMS_relapse_2_7_year _Teriflunomide | Parameters (L208) | Conditional probability ALT test given RRMS relapse for both naïve and experienced patients years 2-7 | 373 | 0.250 | 0.022 | 0.207 | 0.295 | 93 | 280 | Neurologists’ opinion |
| 444 | Cond_pr_Haepatic_test_AST_given_RRMS_relapse_2_7_year _Teriflunomide | Parameters (L209) | Conditional probability AST test given RRMS relapse for both naïve and experienced patients years 2-7 | 373 | 0.250 | 0.022 | 0.207 | 0.295 | 93 | 280 | Neurologists’ opinion |
| 445 | Cond_pr_Haepatic_test_gGT_given_RRMS_relapse_1st_year_Teriflunomide | Parameters (L210) | Conditional probability gGT test given RRMS relapse for both naïve and experienced patients years 2-7 | 373 | 0.250 | 0.022 | 0.207 | 0.295 | 93 | 280 | Neurologists’ opinion |
| 446 | Cond_pr_Brain_MRI_C_given_RRMS_relapse_1st_year_Teriflunomide | Parameters (L211) | Conditional probability brain MRI with contrast given RRMS relapse for both naïve and experienced patients years 2-7 | 373 | 0.125 | 0.017 | 0.093 | 0.160 | 47 | 236 | Neurologists’ opinion |
| 447 | Cond_pr_Brain_plus_spinal_cord_MRI_C_given_RRMS_relapse_1st_year_Teriflunomide | Parameters (L212) | Conditional probability brain plus spinal cord MRI with contrast given RRMS relapse for both naïve and experienced patients years 2-7 | 373 | 0.175 | 0.020 | 0.138 | 0.215 | 65 | 308 | Neurologists’ opinion |
| **Healthcare resources – Probability - RRMS relapse - Follow-up** | | | | | | | | | | | |
| 448 | Cond_pr_Brain_MRI_C_fu_given_RRMS_relapse_1st_year_Teriflunomide | Parameters (L216) | Conditional probability brain MRI with contrast for follow-up given RRMS relapse for both naïve and experienced patients 1st year | 373 | 0.500 | 0.026 | 0.449 | 0.551 | 187 | 187 | Neurologists’ opinion |
| 449 | Cond_pr_Brain_ plus_spinal_cord MRI_fu_given_RRMS_relapse_1st_year_Teriflunomide | Parameters (L217) | Conditional probability brain plus spinal cord MRI with contrast for follow-up given RRMS relapse for both naïve and experienced patients 1st year | 373 | 0.175 | 0.020 | 0.138 | 0.215 | 65 | 308 | Neurologists’ opinion |
| 450 | Cond_pr_Neurologist_visit_fu_given_RRMS_relapse_1st_year_Teriflunomide | Parameters (L218) | Conditional probability brain neurologist visit for follow-up given RRMS relapse for both naïve and experienced patients 1st year | 373 | 0.825 | 0.020 | 0.785 | 0.862 | 308 | 65 | Neurologists’ opinion |
| 451 | Cond_pr_Blood_test_fu_given_RRMS_relapse_2_7_year_Teriflunomide | Parameters (L219) | Conditional probability full blood test for follow-up given RRMS relapse for both naïve and experienced patients years 2-7 | 373 | 0.250 | 0.022 | 0.207 | 0.295 | 93 | 280 | Neurologists’ opinion |
| 452 | Cond_pr_Kidney_test_fu_Creatinine_given_RRMS_relapse_2_7_year_Teriflunomide | Parameters (L220) | Conditional probability creatinine test for follow-up given RRMS relapse for both naïve and experienced patients years 2-7 | 373 | 0.250 | 0.022 | 0.207 | 0.295 | 93 | 280 | Neurologists’ opinion |
| 453 | Cond_pr_Haepatic_test_fu_ALT_given_RRMS_relapse_2_7_year _Teriflunomide | Parameters (L221) | Conditional probability ALT test for follow-up given RRMS relapse for both naïve and experienced patients years 2-7 | 373 | 0.250 | 0.022 | 0.207 | 0.295 | 93 | 280 | Neurologists’ opinion |
| 454 | Cond_pr_Haepatic_test_fu_AST_given_RRMS_relapse_2_7_year _Teriflunomide | Parameters (L222) | Conditional probability AST test for follow-up given RRMS relapse for both naïve and experienced patients years 2-7 | 373 | 0.250 | 0.022 | 0.207 | 0.295 | 93 | 280 | Neurologists’ opinion |
| 455 | Cond_pr_Haepatic_test_fu_gGT_given_RRMS_relapse_2_7_year Teriflunomide | Parameters (L223) | Conditional probability gGT test for follow-up given RRMS relapse for both naïve and experienced patients years 2-7 | 373 | 0.250 | 0.022 | 0.207 | 0.295 | 93 | 280 | Neurologists’ opinion |
| 456 | Cond_pr_Neurologist_visit_fu_given_RRMS_relapse_2_7_year_Teriflunomide | Parameters (L224) | Conditional probability brain neurologist visit for follow-up given RRMS relapse for both naïve and experienced patients years 2-7 | 373 | 0.825 | 0.020 | 0.785 | 0.862 | 308 | 65 | Neurologists’ opinion |
| 457 | Cond_pr_Brain_MRI_C_fu_given_RRMS_relapse_1st_year_Teriflunomide | Parameters (L225) | Conditional probability brain MRI with contrast for follow-up given RRMS relapse for both naïve and experienced patients years 2-7 | 373 | 0.500 | 0.026 | 0.449 | 0.551 | 187 | 187 | Neurologists’ opinion |
| 458 | Cond_pr_Brain_ plus_spinal_cord MRI_fu_given_RRMS_relapse_2_7_year_Teriflunomide | Parameters (L226) | Conditional probability brain plus spinal cord MRI with contrast for follow-up given RRMS relapse for both naïve and experienced patients years 2-7 | 373 | 0.175 | 0.020 | 0.138 | 0.215 | 65 | 308 | Neurologists’ opinion |
| **Healthcare resources – Probability - Adverse events incidence - Teriflunomide** | | | | | | | | | | | |
| 459 | Incidence_nausea_vomiting_1st_year_Teriflunomide | Parameters (L228) | Incidence nausea and vomiting due to Teriflunomide therapy for both naïve and experienced patients 1st year | 373 | 0.246 | 0.022 | 0.204 | 0.291 | 92 | 281 | Neurologists’ opinion |
| 460 | Incidence_nausea_vomiting_2_7_year_Teriflunomide | Parameters (L229) | Incidence nausea and vomiting due to Teriflunomide therapy for both naïve and experienced patients years 2-7 | 373 | 0.246 | 0.022 | 0.204 | 0.291 | 92 | 281 | Neurologists’ opinion |

^a^ Beta distribution was fitted to binomial data (e.g., parameters that encompass two categories, such as needing brain MRI with contrast for follow-up given RRMS relapse or not) ([SI1.]).

**Table SI34 – Materials and Methods - Parameters with statistical distribution for base case analysis, OWSA and PSA**

|  |  |  |  |  |  |  |  |  | **Parameters Beta**  **distribution for PSA^a^** | |  |
| --- | --- | --- | --- | --- | --- | --- | --- | --- | --- | --- | --- |
| **Parameter number** | **Parameter**  **name** | **Spreadsheet**  **(point estimate cell)** | **Parameter**  **description** | **Observations** | **Mean** | **SE** | **LL**  **95%**  **CI** | **UL**  **95%**  **CI** | **Alfa** | **Beta** | **Source** |
| **RRMS naïve and experienced patients on Teriflunomide** | | | | | | | | | | | |
| **Clinical events – Probability - Adverse events incidence - Teriflunomide** | | | | | | | | | | | |
| 461 | Incidence_hair_thinning_1st_year_Teriflunomide | Parameters (L230) | Incidence hair thinning due to Teriflunomide therapy for both naïve and experienced patients 1st year | 373 | 0.043 | 0.010 | 0.024 | 0.065 | 16 | 357 | Neurologists’ opinion |
| 462 | Incidence_fatigue_1st_year_Teriflunomide | Parameters (L231) | Incidence fatigue due to Teriflunomide therapy for both naïve and experienced patients 1st year | 373 | 0.013 | 0.006 | 0.004 | 0.027 | 5 | 368 | Neurologists’ opinion |
| 463 | Incidence_fatigue_2_7_year _Teriflunomide | Parameters (L232) | Incidence fatigue due to Teriflunomide therapy for both naïve and experienced patients years 2-7 | 373 | 0.013 | 0.006 | 0.004 | 0.027 | 5 | 368 | Neurologists’ opinion |
| 464 | Incidence_rachialgy_1st_year_Teriflunomide | Parameters (L233) | Incidence rachialgy due to Teriflunomide therapy for both naïve and experienced patients 1st year | 373 | 0.004 | 0.003 | 0.000 | 0.013 | 2 | 371 | Neurologists’ opinion |
| 465 | Incidence_ rachialgy _2_7_year _Teriflunomide | Parameters (L234) | Incidence rachialgy due to Teriflunomide therapy for both naïve and experienced patients years 2-7 | 373 | 0.004 | 0.003 | 0.000 | 0.013 | 2 | 371 | Neurologists’ opinion |
| 466 | Incidence_hypertension_1st_year_Teriflunomide | Parameters (L235) | Incidence hypertension due to Teriflunomide therapy for both naïve and experienced patients 1st year | 373 | 0.046 | 0.011 | 0.027 | 0.069 | 17 | 356 | Neurologists’ opinion |
| 467 | Incidence_hypertension_2_7_year _Teriflunomide | Parameters (L236) | Incidence hypertension due to Teriflunomide therapy for both naïve and experienced patients years 2-7 | 373 | 0.058 | 0.012 | 0.037 | 0.084 | 22 | 351 | Neurologists’ opinion |
| 468 | Incidence_minor_depression_1st_year_Teriflunomide | Parameters (L237) | Incidence minor depression due to Teriflunomide therapy for both naïve and experienced patients 1st year | 373 | 0.009 | 0.005 | 0.002 | 0.020 | 3 | 370 | Neurologists’ opinion |
| 469 | Incidence_minor_depression_2_7_year _Teriflunomide | Parameters (L238) | Incidence minor depression due to Teriflunomide therapy for both naïve and experienced patients years 2-7 | 373 | 0.004 | 0.003 | 0.002 | 0.013 | 2 | 371 | Neurologists’ opinion |
| 470 | Incidence_haepatoprotection_1st_year_Teriflunomide | Parameters (L239) | Incidence haepatoprotection due to Teriflunomide therapy for both naïve and experienced patients 1st year | 373 | 0.018 | 0.007 | 0.007 | 0.033 | 7 | 366 | Neurologists’ opinion |
| 471 | Incidence_ haepatoprotection _2_7_year _Teriflunomide | Parameters (L240) | Incidence haepatoprotection due to Teriflunomide therapy for both naïve and experienced patients years 2-7 | 373 | 0.018 | 0.007 | 0.007 | 0.033 | 7 | 366 | Neurologists’ opinion |
| 472 | Incidence_infection_1st_year_Teriflunomide | Parameters (L241) | Incidence infection due to Teriflunomide therapy for both naïve and experienced patients 1st year | 373 | 0.042 | 0.010 | 0.024 | 0.064 | 16 | 357 | Neurologists’ opinion |
| 473 | Incidence_ infection _2_7_year _Teriflunomide | Parameters (L242) | Incidence infection due to Teriflunomide therapy for both naïve and experienced patients years 2-7 | 373 | 0.022 | 0.008 | 0.010 | 0.039 | 8 | 365 | Neurologists’ opinion |
| **Healthcare resources – Probability - Adverse events management - Teriflunomide** | | | | | | | | | | | |
| 474 | Cond_pr_Holter_press_given_hypertension_1st_year_Teriflunomide | Parameters (L244) | Conditional probability Holter test given hypertension for both naïve and experienced patients 1st year | 373 | 0.001 | 0.002 | 0.000 | 0.007 | 0 | 373 | Neurologists’ opinion |
| 475 | Cond_pr_ECG_given_hypertension_1st_year_Teriflunomide | Parameters (L245) | Conditional probability ECG test given hypertension for both naïve and experienced patients 1st year | 373 | 0.013 | 0.006 | 0.004 | 0.027 | 5 | 368 | Neurologists’ opinion |
| 476 | Cond_pr_Echocardiogram_given_hypertension_1st_year_Teriflunomide | Parameters (L246) | Conditional probability echocardiogram test given hypertension for both naïve and experienced patients 1st year | 373 | 0.013 | 0.006 | 0.004 | 0.027 | 5 | 368 | Neurologists’ opinion |
| 477 | Cond_pr_ Cardiologist_visit _given_hypertension_1st_year_Teriflunomide | Parameters (L247) | Conditional probability cardiologist visit given hypertension for both naïve and experienced patients 1st year | 373 | 0.034 | 0.009 | 0.018 | 0.054 | 13 | 360 | Neurologists’ opinion |
| 478 | Cond_pr_Drug_given_hypertension_1st_year_Teriflunomide | Parameters (L248) | Conditional probability drug therapy given hypertension for both naïve and experienced patients 1st year | 373 | 0.046 | 0.011 | 0.027 | 0.069 | 17 | 356 | Neurologists’ opinion |
| 479 | Cond_pr_Holter_press_given_hypertension_2_7_year _Teriflunomide | Parameters (L249) | Conditional probability Holter test given hypertension for both naïve and experienced patients years 2-7 | 373 | 0.001 | 0.002 | 0.000 | 0.007 | 0 | 373 | Neurologists’ opinion |
| 480 | Cond_pr_ Cardiologist_visit _given_hypertension_2_7_year Teriflunomide | Parameters (L250) | Conditional probability cardiologist visit given hypertension for both naïve and experienced patients years 2-7 | 373 | 0.046 | 0.011 | 0.027 | 0.069 | 17 | 356 | Neurologists’ opinion |
| 481 | Cond_pr_Drug_given_hypertension_2_7_year _Teriflunomide | Parameters (L251) | Conditional probability drug therapy given hypertension for both naïve and experienced patients years 2-7 | 373 | 0.058 | 0.012 | 0.037 | 0.084 | 22 | 351 | Neurologists’ opinion |
| 482 | Cond_pr_Drug_air_thinning_given_air_thinning_1st_year_Teriflunomide | Parameters (L252) | Conditional probability drug therapy given air thinning n for both naïve and experienced patients 1st year | 373 | 0.018 | 0.007 | 0.007 | 0.033 | 7 | 366 | Neurologists’ opinion |

^a^ Beta distribution was fitted to binomial data (e.g., parameters that encompass two categories, such as needing cardiologist visit given hypertension or not) ([SI1.]).

**Table SI35 – Materials and Methods - Parameters with statistical distribution for base case analysis, OWSA and PSA**

|  |  |  |  |  |  |  |  |  | **Parameters Beta distribution for PSA^a^** | |  |
| --- | --- | --- | --- | --- | --- | --- | --- | --- | --- | --- | --- |
| **Parameter number** | **Parameter**  **name** | **Spreadsheet**  **(point estimate cell)** | **Parameter**  **description** | **Observations** | **Mean** | **SE** | **LL**  **95%**  **CI** | **UL**  **95%**  **CI** | **Alfa** | **Beta** | **Source** |
| **RRMS naïve and experienced patients on Teriflunomide** | | | | | | | | | | | |
| **Healthcare resources – Probability - Adverse events management - Teriflunomide** | | | | | | | | | | | |
| 483 | Cond_pr_Drug_minor_depression_given_minor_depression_1st_year_Teriflunomide | Parameters (L253) | Conditional probability drug therapy given minor depression for both naïve and experienced patients 1st year | 373 | 0.009 | 0.005 | 0.002 | 0.020 | 3 | 370 | Neurologists’ opinion |
| 484 | Cond_pr_Drug_ rachialgy _given_rachialgy_1st_year_Teriflunomide | Parameters (L254) | Conditional probability drug therapy given rachialgy for both naïve and experienced patients 1st year | 373 | 0.004 | 0.003 | 0.002 | 0.013 | 2 | 371 | Neurologists’ opinion |
| 485 | Cond_pr_Drug_minor_depression_given_minor_depression_1st_year_Teriflunomide | Parameters (L255) | Conditional probability drug therapy given minor depression for both naïve and experienced patients 1st year | 373 | 0.009 | 0.005 | 0.002 | 0.020 | 3 | 370 | Neurologists’ opinion |
| 486 | Cond_pr_Drug_minor_depression_given_minor_depression_2_7_year _Teriflunomide | Parameters (L256) | Conditional probability drug therapy given minor depression for both naïve and experienced patients years 2-7 | 373 | 0.009 | 0.005 | 0.002 | 0.020 | 3 | 370 | Neurologists’ opinion |
| 487 | Cond_pr_Drug_given_hepatoprotection_1st_year_Teriflunomide | Parameters (L257) | Conditional probability drug therapy given hepatoprotection for both naïve and experienced patients years 2-7 | 373 | 0.018 | 0.007 | 0.007 | 0.033 | 7 | 366 | Neurologists’ opinion |
| 488 | Cond_pr_Haepatic_ultrasound_given_hepatoprotection_1st_year_Teriflunomide | Parameters (L258) | Conditional probability haepatic ultrasound given hepatoprotection for both naïve and experienced patients 1st year | 373 | 0.018 | 0.007 | 0.007 | 0.033 | 7 | 366 | Neurologists’ opinion |
| 489 | Cond_pr_Haepatic_test_ALT_given_hepatoprotection_1st_year_Teriflunomide | Parameters (L259) | Conditional probability ALT test given hepatoprotection for both naïve and experienced patients 1st year | 373 | 0.018 | 0.007 | 0.007 | 0.033 | 7 | 366 | Neurologists’ opinion |
| 490’ | Cond_pr_Haepatic_test_AST_given_hepatoprotection_1st_year_Teriflunomide | Parameters (L260) | Conditional probability AST test given hepatoprotection for both naïve and experienced patients 1st year | 373 | 0.018 | 0.007 | 0.007 | 0.033 | 7 | 366 | Neurologists’ opinion |
| 491 | Cond_pr_Haepatic_test_gGT_given_hepatoprotection_1st_year_Teriflunomide | Parameters (L261) | Conditional probability gGT test given hepatoprotection for both naïve and experienced patients 1st year | 373 | 0.018 | 0.007 | 0.007 | 0.033 | 7 | 366 | Neurologists’ opinion |
| 492 | Cond_pr_Haepatic_test_total_bilirubin_given_hepatoprotection_1st_year_Teriflunomide | Parameters (L262) | Conditional probability total bilirubin test given hepatoprotection for both naïve and experienced patients 1st year | 373 | 0.018 | 0.007 | 0.007 | 0.033 | 7 | 366 | Neurologists’ opinion |
| 493 | Cond_pr_Haepatic_test_albumine_given_hepatoprotection_1st_year_Teriflunomide | Parameters (L263) | Conditional probability albumine test given hepatoprotection for both naïve and experienced patients 1st year | 373 | 0.018 | 0.007 | 0.007 | 0.033 | 7 | 366 | Neurologists’ opinion |
| 494 | Cond_pr_Haepatologist_visit_given_hepatoprotection_1st_year_Teriflunomide | Parameters (L264) | Conditional probability haepatologist visit given hepatoprotection for both naïve and experienced patients 1st year | 373 | 0.018 | 0.007 | 0.007 | 0.033 | 7 | 366 | Neurologists’ opinion |
| 495 | Cond_pr_Drug_given_hepatoprotection_2_7_year_Teriflunomide | Parameters (L265) | Conditional probability drug therapy given hypertension for both naïve and experienced patients years 2-7 | 373 | 0.018 | 0.007 | 0.007 | 0.033 | 7 | 366 | Neurologists’ opinion |
| 496 | Cond_pr_Haepatic_ultrasound_given_hepatoprotection_2_7_year year_Teriflunomide | Parameters (L266) | Conditional probability haepatic ultrasound given hepatoprotection for both naïve and experienced patients years 2-7 | 373 | 0.018 | 0.007 | 0.007 | 0.033 | 7 | 366 | Neurologists’ opinion |
| 497 | Cond_pr_Haepatic_test_ALT_given_hepatoprotection_2_7_year _Teriflunomide | Parameters (L267) | Conditional probability ALT test given hepatoprotection for both naïve and experienced patients years 2-7 | 373 | 0.018 | 0.007 | 0.007 | 0.033 | 7 | 366 | Neurologists’ opinion |
| 498 | Cond_pr_Haepatic_test_AST_given_hepatoprotection_2_7_year _Teriflunomide | Parameters (L268) | Conditional probability AST test given hepatoprotection for both naïve and experienced patients years 2-7 | 373 | 0.018 | 0.007 | 0.007 | 0.033 | 7 | 366 | Neurologists’ opinion |
| 499 | Cond_pr_Haepatic_test_gGT_given_hepatoprotection_2_7_year _Teriflunomide | Parameters (L269) | Conditional probability gGT test given hepatoprotection for both naïve and experienced patients years 2-7 | 373 | 0.018 | 0.007 | 0.007 | 0.033 | 7 | 366 | Neurologists’ opinion |
| 500 | Cond_pr_Haepatic_test_total_bilirubin_given_hepatoprotection__2_7_year Teriflunomide | Parameters (L270) | Conditional probability total bilirubin test given hepatoprotection for both naïve and experienced patients years 2-7 | 373 | 0.018 | 0.007 | 0.007 | 0.033 | 7 | 366 | Neurologists’ opinion |
| 501 | Cond_pr_Haepatic_test_albumine_given_hepatoprotection_2_7_year_Teriflunomide | Parameters (L271) | Conditional probability albumine test given hepatoprotection for both naïve and experienced patients years 2-7 | 373 | 0.018 | 0.007 | 0.007 | 0.033 | 7 | 366 | Neurologists’ opinion |

^a^ Beta distribution was fitted to binomial data (e.g., parameters that encompass two categories, such as needing drug therapy given minor depression or not) and utility values ([SI1.]).

**Table SI36 – Materials and Methods - Parameters with statistical distribution for base case analysis, OWSA and PSA**

|  |  |  |  |  |  |  |  |  | **Parameters Beta distribution for PSA^a^** | |  |
| --- | --- | --- | --- | --- | --- | --- | --- | --- | --- | --- | --- |
| **Parameter number** | **Parameter**  **name** | **Spreadsheet**  **(point estimate cell)** | **Parameter**  **description** | **Observations** | **Mean** | **SE** | **LL 95%**  **CI** | **UL 95%**  **CI** | **Alfa** | **Beta** | **Source** |
| **RRMS naïve and experienced patients on Teriflunomide** | | | | | | | | | | | |
| **Healthcare resources – Probability - Adverse events management - Teriflunomide** | | | | | | | | | | | |
| 502 | Cond_pr_Haepatologist_visit_given_hepatoprotection_2_7_year _Teriflunomide | Parameters (L272) | Conditional probability haepatologist visit given hepatoprotection for both naïve and experienced patients years 2-7 | 373 | 0.018 | 0.007 | 0.007 | 0.033 | 7 | 366 | Neurologists’ opinion |
| 503 | Cond_pr_Drug_infection_given_infection _1st_year_Teriflunomide | Parameters (L273) | Conditional probability drug therapy given infection for both naïve and experienced patients 1st year | 373 | 0.042 | 0.010 | 0.024 | 0.064 | 16 | 357 | Neurologists’ opinion |
| 504 | Cond_pr_Infectivologist_visit_given_infection_1st_year_Teriflunomide | Parameters (L274) | Conditional probability infectivologist visit given infection for both naïve and experienced patients 1st year | 373 | 0.020 | 0.007 | 0.008 | 0.036 | 7 | 366 | Neurologists’ opinion |
| 505 | Cond_pr_Drug_infection_given_infection _2_7_year _Teriflunomide | Parameters (L275) | Conditional probability drug therapy given infection for both naïve and experienced patients years 2-7 | 373 | 0.042 | 0.010 | 0.024 | 0.064 | 16 | 357 | Neurologists’ opinion |
| **Clinical events – Probability - Switch to other drugs due to Teriflunomide ineffectiveness in naïve patients - All drugs** | | | | | | | | | | | |
| 506 | Pr_Faillure_naïve_2_7_year_Teriflunomide | Parameters (L277) | Probability Teriflunomide lacks effectiveness in RRMS naïve patients years 2_7 | 153 | 0. 100 | 0.024 | 0.058 | 0.152 | 15 | 138 | Neurologists’ opinion |
| **Switch to other drugs due to Teriflunomide ineffectiveness in naïve patients - Natalizumab** | | | | | | | | | | | |
| **Healthcare resources – Probability - Pre-treatment assessment - Natalizumab** | | | | | | | | | | | |
| 507 | Cond_pr_pre_Natalizumab_haepatic_test_total_bilirubin_given_fail_naïve_2_7_year_Teriflunomide | Parameters (L289) | Conditional probability total bilirubin test pre-treatment assessment Natalizumab given poor response to Teriflunomide naïve patients years 2_7 | 15 | 0.333 | 0.121 | 0.129 | 0.179 | 5 | 10 | Neurologists’ opinion |
| **Healthcare resources – Probability - During treatment assessment - Natalizumab** | | | | | | | | | | | |
| 508 | Cond_pr_during_Natalizumab_haepatic_test_total_bilirubin_given_fail_naïve_2_7_year_Teriflunomide | Parameters (L299) | Conditional probability total bilirubin test during treatment assessment Natalizumab given poor response to Teriflunomide naïve patients years 2_7 | 15 | 0.333 | 0.121 | 0.129 | 0.179 | 5 | 10 | Neurologists’ opinion |
| 509 | Cond_pr_during_Natalizumab_sierology_test_JCV_given_fail_naïve_2_7_year_Teriflunomide | Parameters (L303) | Conditional probability JCV test during treatment assessment Natalizumab given poor response to Teriflunomide naïve patients years 2_7 | 15 | 0.667 | 0.121 | 0.421 | 0.871 | 10 | 5 | Neurologists’ opinion |
| **Healthcare resources – Probability - Administration setting - Natalizumab** | | | | | | | | | | | |
| 510 | Cond_pr_adm_DH_Natalizumab_given_fail_naïve_2_7_year_Teriflunomide | Parameters (L305) | Conditional probability DH administration Natalizumab given poor response to Teriflunomide naïve patients years 2_7 | 15 | 0.667 | 0.121 | 0.421 | 0.871 | 10 | 5 | Neurologists’ opinion |
| 511 | Cond_pr_adm_Outpatient_Natalizumab_given_fail_naïve_2_7_year_Teriflunomide | Parameters (L306) | Conditional probability outpatient administration Natalizumab given poor response to Teriflunomide naïve patients years 2_7 | 15 | 0.333 | 0.121 | 0.129 | 0.179 | 5 | 10 | Neurologists’ opinion |
| **Healthcare resources – Probability - Premedication - Natalizumab** | | | | | | | | | | | |
| 512 | Cond_pr_premed_corticosteroids_Natalizumab_given_fail_naïve_2_7_year_Teriflunomide | Parameters (L308) | Conditional probability premedication with corticosteroids Natalizumab given poor response to Teriflunomide naïve patients years 2_7 | 15 | 0.667 | 0.121 | 0.421 | 0.871 | 10 | 5 | Neurologists’ opinion |
| 513 | Cond_pr_premed_time_nurse_Natalizumab_given_fail_naïve_2_7_year_Teriflunomide | Parameters (L309) | Conditional probability nurse time for premedication Natalizumab given poor response to Teriflunomide naïve patients years 2_7 | 15 | 0.667 | 0.121 | 0.421 | 0.871 | 10 | 5 | Neurologists’ opinion |

^a^ Beta distribution was fitted to binomial data (e.g., parameters that encompass two categories, such as needing nurse’s time before Natalizumab administration or not) and utility values ([SI1.]).

**Table SI37 – Materials and Methods - Parameters with statistical distribution for base case analysis, OWSA and PSA**

|  |  |  |  |  |  |  |  |  | **Parameters Beta distribution for PSA^a^** | |  |
| --- | --- | --- | --- | --- | --- | --- | --- | --- | --- | --- | --- |
| **Parameter number** | **Parameter**  **name** | **Spreadsheet**  **(point estimate cell)** | **Parameter**  **description** | **Observations** | **Mean** | **SE** | **LL 95%**  **CI** | **UL 95%**  **CI** | **Alfa** | **Beta** | **Source** |
| **RRMS naïve and experienced patients on Teriflunomide** | | | | | | | | | | | |
| **Switch to other DMTs due to Teriflunomide ineffectiveness in naïve patients – All DMTs** | | | | | | | | | | | |
| **Healthcare resources – Probability - Administration time - Natalizumab** | | | | | | | | | | | |
| 514 | Cond_pr_adm_time_pharmacist_Natalizumab_given_fail_naïve_2_7_year_Teriflunomide | Parameters (L311) | Conditional probability pharmacist time for administration Natalizumab given poor response to Teriflunomide naïve patients years 2_7 | 15 | 0.333 | 0.121 | 0.129 | 0.179 | 5 | 10 | Neurologists’ opinion |
| **Healthcare resources – Probability - Administration disposable - Natalizumab** | | | | | | | | | | | |
| 515 | Cond_pr_adm_CVC_Natalizumab_given_fail_naïve_2_7_year_Teriflunomide | Parameters (L317) | Conditional probability CVC for administration Natalizumab given poor response to Teriflunomide naïve patients years 2_7 | 15 | 0.333 | 0.121 | 0.129 | 0.179 | 5 | 10 | Neurologists’ opinion |
| 516 | Cond_pr_adm_gauze_Natalizumab_given_fail_naïve_2_7_year_Teriflunomide | Parameters (L318) | Conditional probability gauze for administration Natalizumab given poor response to Teriflunomide naïve patients years 2_7 | 15 | 0.333 | 0.121 | 0.129 | 0.179 | 5 | 10 | Neurologists’ opinion |
| **Healthcare resources – Probability - Postadministration disposable - Natalizumab** | | | | | | | | | | | |
| 517 | Cond_pr_postad_physio_Natalizumab_given_fail_naïve_2_7_year_Teriflunomide | Parameters (L320) | Conditional probability physiological solution postadministration Natalizumab given poor response to Teriflunomide naïve patients years 2_7 | 15 | 0.667 | 0.121 | 0.421 | 0.871 | 10 | 5 | Neurologists’ opinion |
| **Healthcare resources – Probability - Postadministration time - Ocrelizumab** | | | | | | | | | | | |
| 518 | Cond_pr_postad_time_nurse_Ocrelizumab_given_fail_naïve_2_7_year_Teriflunomide | Parameters (L359) | Conditional probability nurse time postadministration Ocrelizumab given poor response to Teriflunomide naïve patients years 2_7 | 6 | 0.333 | 0.192 | 0.053 | 0.716 | 2 | 4 | Neurologists’ opinion |
| **Healthcare resources – Probability - Pre-treatment assessment - Alemtuzumab** | | | | | | | | | | | |
| 519 | Cond_pr_pre_Alemtuzumab_sierology_test_HPV_given_fail_naïve_2_7_year_Teriflunomide | Parameters (L367) | Conditional probability HPV test Alemtuzumab given poor response to Teriflunomide female naïve patients years 2_7 | 5 | 0.818 | 0.173 | 0.420 | 0.996 | 4 | 1 | Neurologists’ opinion |
| **Healthcare resources – Probability - Postadministration time - Alemtuzumab** | | | | | | | | | | | |
| 520 | Cond_pr_postad_time_nurse_Alemtuzumab_given_fail_naïve_2_7_year_Teriflunomide | Parameters (L381) | Conditional probability nurse time postadministration Alemtuzumab given poor response to Teriflunomide naïve patients years 2_7 | 5 | 0.250 | 0.195 | 0.015 | 0.665 | 1 | 4 | Neurologists’ opinion |
| **Utility** | | | | | | | | | | | |
| 521 | First_year_bkd_utility_naïve | Parameters (L728) | Background utility naïve patients 1st year | 153 | 0.705 | 0.037 | 0.631 | 0.774 | 108 | 45 | [SI9.] |
| 522 | First_year_bkd_utility_exp | Parameters (L729) | Background utility experienced patients 1st year | 220 | 0.574 | 0.033 | 0.508 | 0.638 | 126 | 94 | [SI9.] |
| 523 | Second_year_bkd_utility_naïve | Parameters (L730) | Background utility naïve patients 2nd year | 153 | 0.574 | 0.040 | 0.495 | 0.651 | 88 | 65 | [SI9.] |
| 524 | Second_year_bkd_utility_exp | Parameters (L731) | Background utility experienced patients 2nd year | 220 | 0.610 | 0.033 | 0.545 | 0.673 | 134 | 86 | [SI9.] |
| 525 | Third_year_bkd_utility_naïve | Parameters (L732) | Background utility naïve patients 3rd year | 153 | 0.574 | 0.040 | 0.495 | 0.651 | 88 | 65 | [SI9.] |
| 526 | Third_year_bkd_utility_exp | Parameters (L733) | Background utility experienced patients 3rd year | 220 | 0.610 | 0.033 | 0.545 | 0.673 | 134 | 86 | [SI9.] |
| 527 | Fourth_year_bkd_utility_naïve | Parameters (L734) | Background utility naïve patients 4th year | 153 | 0.574 | 0.040 | 0.495 | 0.651 | 88 | 65 | [SI9.] |
| 528 | Fourth_year_bkd_utility_exp | Parameters (L735) | Background utility experienced patients 4th year | 220 | 0.518 | 0.034 | 0.452 | 0.584 | 114 | 106 | [SI9.] |

^a^ Beta distribution was fitted to binomial data (e.g., parameters that encompass two categories, such as needing nurse’s time after Alemtuzumab administration or not) and utility values ([SI1.]).

**Table SI38 – Materials and Methods - Parameters with statistical distribution for base case analysis, OWSA and PSA**

|  |  |  |  |  |  |  |  |  | **Parameters Beta distribution for PSA^a^** | |  |
| --- | --- | --- | --- | --- | --- | --- | --- | --- | --- | --- | --- |
| **Parameter number** | **Parameter**  **name** | **Spreadsheet**  **(point estimate cell)** | **Parameter**  **description** | **Observations** | **Mean** | **SE** | **LL 95% CI** | **UL 95% CI** | **Alfa** | **Beta** | **Source** |
| **RRMS naïve and experienced patients on Teriflunomide** | | | | | | | | | | | |
| **Utility** | | | | | | | | | | | |
| 529 | Fifth_year_bkd_utility_naïve | Parameters (L736) | Background utility naïve patients 5th year | 153 | 0.574 | 0.040 | 0.495 | 0.651 | 88 | 65 | [SI9.] |
| 530 | Fifth_year_bkd_utility_exp | Parameters (L737) | Background utility experienced patients 5th year | 220 | 0.518 | 0.034 | 0.452 | 0.584 | 114 | 106 | [SI9.] |
| 531 | Sixth_year_bkd_utility_naïve | Parameters (L738) | Background utility naïve patients 6th year | 153 | 0.574 | 0.040 | 0.495 | 0.651 | 88 | 65 | [SI9.] |
| 532 | Sixth_year_bkd_utility_exp | Parameters (L739) | Background utility experienced patients 6th year | 220 | 0.462 | 0.034 | 0.397 | 0.528 | 101 | 118 | [SI9.] |
| 533 | Seventh_year_bkd_utility_naïve | Parameters (L740) | Background utility naïve patients 7th year | 153 | 0.610 | 0.039 | 0.535 | 0.686 | 93 | 60 | [SI9.] |
| 534 | Seventh_year_bkd_utility_exp | Parameters (L741) | Background utility experienced patients 7th year | 220 | 0.297 | 0.031 | 0.239 | 0.359 | 65 | 155 | [SI9.] |
| 535 | Male_related_utility | Parameters (L742) | Male patients related utility for both naïve and experienced patients years 1-7 | 83 | 0.017 | 0.014 | 0.001 | 0.054 | 1 | 82 | [SI9.] |
| 536 | Disease_experience_utility | Parameters (L743) | Disease experience utility for both naïve and experienced patients years 1-7 | 373 | 0.002 | 0.002 | 0.000 | 0.008 | 1 | 372 | [SI9.] |
| 537 | Nausea_vomiting_disutility_Teriflunomide | Parameters (L744) | Nausea and vomiting disutility for both naïve and experienced patients years 1-7 | 373 | 0.001 | 0.002 | 0.000 | 0.006 | 0 | 373 | [SI10.] |
| 538 | Hair_thinning_disutility_Teriflunomide | Parameters (L745) | Nausea and vomiting disutility for both naïve and experienced patients years 1-7 | 373 | 0.114 | 0.016 | 0.084 | 0.148 | 43 | 330 | [SI10.] |
| 539 | Fatigue_disutility_Teriflunomide | Parameters (L746) | Fatigue disutility for both naïve and experienced patients years 1-7 | 373 | 0.001 | 0.002 | 0.000 | 0.007 | 1 | 372 | [SI10.] |
| 540 | Rachyalgia_disutility_Teriflunomide | Parameters (L747) | Rachyalgia disutility for both naïve and experienced patients years 1-7 | 373 | 0.003 | 0.003 | 0.000 | 0.011 | 1 | 372 | [SI10.] |
| 541 | Hypertension_disutility_Teriflunomide | Parameters (L748) | Hypertension disutility for both naïve and experienced patients years 1-7 | 373 | 0.037 | 0.010 | 0.020 | 0.059 | 14 | 359 | [SI11.] |
| 542 | Minor_depression_disutility_Teriflunomide | Parameters (L749) | Minor depression disutility for both naïve and experienced patients years 1-7 | 373 | 0.380 | 0.025 | 0.331 | 0.430 | 142 | 231 | [SI12.] |
| 543 | Pneumonia_disutility_Teriflunomide | Parameters (L750) | Pneumonia disutility for both naïve and experienced patients years 1-7 | 373 | 0.440 | 0.026 | 0.390 | 0.491 | 164 | 209 | [SI13.] |
| 544 | Haepatoprotection_disutility_Teriflunomide | Parameters (L751) | Haepatoprotectiondisutility for both naïve and experienced patients years 1-7 | 373 | 0.250 | 0.022 | 0.207 | 0.295 | 93 | 280 | [SI13.] |
| 545 | Recent_relapse_disutility | Parameters (L752) | Recent relapse disutility for both naïve and experienced patients years 1-7 | 373 | 0.071 | 0.047 | 0.207 | 0.099 | 26 | 347 | [SI9.] |

^a^ Beta distribution was fitted to utility and disutility values ([SI1.]).

**Table SI39 – Materials and Methods - Parameters with statistical distribution for base case analysis, OWSA and PSA**

|  |  |  |  |  |  |  |  |  | **Parameter Dirichlet distribution for PSA^a^** | |  |  | |
| --- | --- | --- | --- | --- | --- | --- | --- | --- | --- | --- | --- | --- | --- |
| **Parameter**  **number** | **Parameter**  **name** | **Spreadsheet**  **(point estimate cell)** | **Parameter**  **description** | **Observations** | **Mean** | **SE** | **LI 95% CI** | **UL 95% CI** | **Alfa** |  |  |  | **Source** |
| **RRMS naïve and experienced patients on Teriflunomide** | | | | | | | | | | | | | |
| **Switch to other DMTs due to Teriflunomide ineffectiveness in naïve patients – All DMTs** | | | | | | | | | | | | | |
| 546 | Cond_pr_Natalizumab_given_fail_naïve_2_7_year_Teriflunomide | Parameters (L278) | Conditional probability Natalizumab given poor response to Teriflunomide naïve patients years 2_7 | 15 | 0.300 | 0.117 | 0.106 | 0.543 | 5 |  |  |  | Neurologists’ opinion |
| 547 | Cond_pr_Fingolimod_given_fail_naïve_2_7_year_Teriflunomide | Parameters (L279) | Conditional probability Fingolimod given poor response to Teriflunomide naïve patients years 2_7 | 11 | 0.300 | 0.140 | 0.080 | 0.590 | 3 |  |  |  | Neurologists’ opinion |
| 548 | Cond_pr_Cladribine_given_fail_naïve_2_7_year_Teriflunomide | Parameters (L280) | Conditional probability Cladribine given poor response to Teriflunomide naïve patients years 2_7 | 7 | 0.200 | 0.146 | 0.017 | 0.528 | 1 |  |  |  | Neurologists’ opinion |
| 549 | Cond_pr_Ocrelizumab_given_fail_naïve_2_7_year_Teriflunomide | Parameters (L281) | Conditional probability Ocrelizumab given poor response to Teriflunomide naïve patients years 2_7 | 6 | 0.175 | 0.155 | 0.006 | 0.534 | 1 |  |  |  | Neurologists’ opinion |
| 550 | Cond_pr_ Alemtuzumab _given_fail_naïve_2_7_year_Teriflunomide | Parameters (L282) | Conditional probability Alemtuzumab given poor response to Teriflunomide naïve patients years 2_7 | 5 | 0.025 | 0.070 | 0.000 | 0.226 | 0 |  |  |  | Neurologists’ opinion |

^a^ Dirichlet distribution was assigned to multinomial data (e.g., parameters that include ≥3 categories, such as switching to one out of the five different DMTs given Terifunomide ineffectiveness in RRMS naïve patients) ([SI1.]).

**Table SI40 – Materials and Methods - Parameters with statistical distribution for base case analysis, OWSA and PSA**

|  |  |  |  |  | |  |  |  |  | **Parameters Gamma distribution per PSA^a^** | |  |
| --- | --- | --- | --- | --- | --- | --- | --- | --- | --- | --- | --- | --- |
| **Parameter number** | **Parameter**  **name** | **Spreadsheet**  **(point estimate cell)** | **Parameter**  **description** | **Observations** | | **Mean** | **SE^b^** | **LL 95% CI** | **UL 95% CI** | **Alfa** | **Beta** | **Source** |
| **RRMS naïve and experienced patients on Teriflunomide** | | | | | | | | | | | | |
| **Healthcare resources - Liver monitoring - Teriflunomide** | | | | | | | | | | | | |
| 551 | Vol_Haepatic_test_ALT_monitoring_1st_year_Teriflunomide | Parameters (L414) | Number of ALT tests for liver monitoring Teriflunomide for both naïve and experienced patients 1st year | 373 | 10.50 | | 4.20 | 3.95 | 20.19 | 6 | 2 | Neurologists’ opinion |
| 552 | Vol_Haepatic_test_AST_monitoring_1st_year_Teriflunomide | Parameters (L415) | Number of AST tests for liver monitoring Teriflunomide for both naïve and experienced patients 1st year | 373 | 9.33 | | 3.73 | 3.51 | 17.95 | 6 | 1 | Neurologists’ opinion |
| 553 | Vol_Haepatic_test_gGT_monitoring_1st_year_Teriflunomide | Parameters (L416) | Number of gGT tests for liver monitoring Teriflunomide for both naïve and experienced patients 1st year | 373 | 9.33 | | 3.73 | 3.51 | 17.95 | 6 | 1 | Neurologists’ opinion |
| 554 | Vol_Haepatic_test_Total_bilirubin_monitoring_1st_year_Teriflunomide | Parameters (L417) | Number of total bilirubin tests for liver monitoring Teriflunomide for both naïve and experienced patients 1st year | 373 | 7.00 | | 2.80 | 2.63 | 13.46 | 6 | 1 | Neurologists’ opinion |
| 555 | Vol_Haepatic_test_Alkaline_phosphatase_monitoring_1st_year_Teriflunomide | Parameters (L418) | Number of alkaline phosphatase tests for liver monitoring Teriflunomide for both naïve and experienced patients 1st year | 373 | 7.00 | | 2.80 | 2.63 | 13.46 | 6 | 1 | Neurologists’ opinion |
| 556 | Vol_Haepatic_test_ALT_monitoring_2_7_year_Teriflunomide | Parameters (L419) | Number of ALT tests for liver monitoring Teriflunomide for both naïve and experienced patients years 2_7 | 373 | 3.00 | | 1.20 | 1.13 | 5.77 | 6 | 0 | Neurologists’ opinion |
| 557 | Vol_Haepatic_test_AST_monitoring_2_7_year_Teriflunomide | Parameters (L420) | Number of AST tests for liver monitoring Teriflunomide for both naïve and experienced patients years 2_7 | 373 | 2.67 | | 1.07 | 1.00 | 5.13 | 6 | 0 | Neurologists’ opinion |
| 558 | Vol_Haepatic_test_gGT_monitoring_2_7_year_Teriflunomide | Parameters (L421) | Number of gGT tests for liver monitoring Teriflunomide for both naïve and experienced patients years 2_7 | 373 | 2.67 | | 1.07 | 1.00 | 5.13 | 6 | 0 | Neurologists’ opinion |
| 559 | Vol_Haepatic_test_Total_bilirubin_monitoring_2_7_year_Teriflunomide | Parameters (L422) | Number of total bilirubin tests for liver monitoring Teriflunomide for both naïve and experienced patients years 2_7 | 373 | 2.00 | | 0.80 | 0.75 | 3.85 | 6 | 0 | Neurologists’ opinion |
| 560 | Vol_Haepatic_test_Alkaline_phosphatase_monitoring_2_7_year_Teriflunomide | Parameters (L423) | Number of alkaline phosphatase tests for liver monitoring Teriflunomide for both naïve and experienced patients years 2_7 | 373 | 2.00 | | 0.80 | 0.75 | 3.85 | 6 | 0 | Neurologists’ opinion |
| **Healthcare resources - RRMS no relapse - Follow-up** | | | | | | | | | | | | |
| 561 | Vol_Brain_MRI_NOC_fu_RRMS_NO_relapse_naïve_1st_year | Parameters (L425) | Number of brain MRIs without contrast naïve RRMS patients for follow-up with no relapse during Teriflunomide treatment 1st year | 153 | 3.83 | | 1.53 | 1.44 | 7.37 | 6 | 1 | Neurologists’ opinion |
| 562 | Vol_Brain_MRI_NOC_fu_RRMS_NO_relapse_exp_1st_year | Parameters (L426) | Number of brain MRIs without contrast experienced RRMS patients for follow-up with no relapse during Teriflunomide treatment 1st year | 220 | 3.50 | | 1.40 | 1.32 | 6.73 | 6 | 1 | Neurologists’ opinion |

^a^ Gamma distribution represents uncertainty in resource consumption ([SI1.]).

^b^ If the analytical calculation of SE was unfeasible, a CV=40% of the sample mean has been imposed ([SI1.], [SI14.]).

**Table SI41 – Materials and Methods - Parameters with statistical distribution for base case analysis, OWSA and PSA**

|  |  |  |  |  | |  |  |  |  | | **Parameters Gamma distribution per PSA^a^** | |  |
| --- | --- | --- | --- | --- | --- | --- | --- | --- | --- | --- | --- | --- | --- |
| **Parameter number** | **Parameter**  **name** | **Spreadsheet**  **(point estimate cell)** | **Parameter**  **description** | **Observations** | | **Mean** | **SE^b^** | **LL 95% CI** | **UL 95% CI** | | **Alfa** | **Beta** | **Source** |
| **RRMS naïve and experienced patients on Teriflunomide** | | | | | | | | | | | | | |
| **Healthcare resources - RRMS no relapse - Follow-up** | | | | | | | | | | | | | |
| 563 | Vol_Brain_MRI_C_fu_RRMS_NO_relapse_naïve_1st_year | Parameters (L427) | Number of brain MRIs with contrast naïve RRMS patients for follow-up with no relapse during Teriflunomide treatment 1st year | | 153 | 0.65 | 0.26 | 0.25 | 1.25 | 6 | | 0 | Neurologists’ opinion |
| 564 | Vol_Brain_MRI_C_fu_RRMS_NO_relapse_exp_1st_year | Parameters (L428) | Number of brain MRIs with contrast experienced RRMS patients for follow-up with no relapse during Teriflunomide treatment 1st year | | 220 | 0.65 | 0.26 | 0.25 | 1.25 | 6 | | 0 | Neurologists’ opinion |
| 565 | Vol_Brain_plus_spinal_cord_MRI_fu_RRMS_NO_relapse_naïve_1st_year | Parameters (L429) | Number of brain plus spinal cord MRIs with contrast naïve RRMS patients for follow-up with no relapse during Teriflunomide treatment 1st year | | 153 | 2.00 | 0.80 | 0.75 | 3.85 | 6 | | 0 | Neurologists’ opinion |
| 566 | Vol_Brain_plus_spinal_cord_MRI_fu_RRMS_NO_relapse_exp_1st_year | Parameters (L430) | Number of brain plus spinal cord MRIs with contrast experienced RRMS patients for follow-up with no relapse during Teriflunomide treatment 1st year | | 220 | 1.00 | 0.40 | 0.38 | 1.92 | 6 | | 0 | Neurologists’ opinion |
| 567 | Vol_Blood_test_fu_RRMS_NO_relapse_naïve_1st_year | Parameters (L431) | Number of blood tests naïve RRMS patients for follow-up with no relapse during Teriflunomide treatment 1st year | | 153 | 2.00 | 0.80 | 0.75 | 3.85 | 6 | | 0 | Neurologists’ opinion |
| 568 | Vol_Blood_test_fu_RRMS_NO_relapse_exp_1st_year | Parameters (L432) | Number of blood tests experienced RRMS patients for follow-up with no relapse during Teriflunomide treatment 1st year | | 220 | 2.00 | 0.80 | 0.75 | 3.85 | 6 | | 0 | Neurologists’ opinion |
| 569 | Vol_Haepatic_test_ALT_fu_RRMS_NO_relapse_naïve_1st_year_Teriflunomide | Parameters (L433) | Number of ALT tests naïve RRMS patients for follow-up with no relapse during Teriflunomide treatment 1st year | | 153 | 2.00 | 0.80 | 0.75 | 3.85 | 6 | | 0 | Neurologists’ opinion |
| 570 | Vol_Haepatic_test_ALT_fu_RRMS_NO_relapse_exp_1st_year_Teriflunomide | Parameters (L434) | Number of ALT tests experienced RRMS patients for follow-up with no relapse during Teriflunomide treatment 1st year | | 220 | 2.00 | 0.80 | 0.75 | 3.85 | 6 | | 0 | Neurologists’ opinion |
| 571 | Vol_Haepatic_test_AST_fu_RRMS_NO_relapse_naïve_1st_year_Teriflunomide | Parameters (L435) | Number of AST tests naïve RRMS patients for follow-up with no relapse during Teriflunomide treatment 1st year | | 153 | 2.00 | 0.80 | 0.75 | 3.85 | 6 | | 0 | Neurologists’ opinion |
| 572 | Vol_Haepatic_test_AST_fu_RRMS_NO_relapse_exp_1st_year_Teriflunomide | Parameters (L436) | Number of AST tests experienced RRMS patients for follow-up with no relapse during Teriflunomide treatment 1st year | | 220 | 2.00 | 0.80 | 0.75 | 3.85 | 6 | | 0 | Neurologists’ opinion |
| 573 | Vol_Haepatic_test_gGT_fu_RRMS_NO_relapse_naïve_1st_year_Teriflunomide | Parameters (L437) | Number of gGT tests naïve RRMS patients for follow-up with no relapse during Teriflunomide treatment 1st year | | 153 | 2.00 | 0.80 | 0.75 | 3.85 | 6 | | 0 | Neurologists’ opinion |
| 574 | Vol_Haepatic_test_gGT_fu_RRMS_NO_relapse_exp_1st_year_Teriflunomide | Parameters (L438) | Number of gGT tests experienced RRMS patients for follow-up with no relapse during Teriflunomide treatment 1st year | | 220 | 2.00 | 0.80 | 0.75 | 3.85 | 6 | | 0 | Neurologists’ opinion |

^a^ Gamma distribution represents uncertainty in resource consumption ([SI1.]).

^b^ If the analytical calculation of SE was unfeasible, a CV=40% of the sample mean has been imposed ([SI1.], [SI14.]).

**Table SI42 – Materials and Methods - Parameters with statistical distribution for base case analysis, OWSA and PSA**

|  |  |  |  |  |  |  |  |  | **Parameters Gamma distribution per PSA^a^** | |  |
| --- | --- | --- | --- | --- | --- | --- | --- | --- | --- | --- | --- |
| **Parameter number** | **Parameter**  **name** | **Spreadsheet**  **(point estimate cell)** | **Parameter**  **description** | **Observations** | **Mean** | **SE^b^** | **LL 95% CI** | **UL 95% CI** | **Alfa** | **Beta** | **Source** |
| **RRMS naïve and experienced patients on Teriflunomide** | | | | | | | | | | | |
| **Healthcare resources - RRMS no relapse - Follow-up** | | | | | | | | | | | |
| 575 | Vol_Haepatic_test_Alkaline_phosphatase_fu_RRMS_NO_relapse_naïve_1st_year_Teriflunomide | Parameters (L439) | Number of alkaline phosphatase tests naïve RRMS patients for follow-up with no relapse during Teriflunomide treatment 1st year | 153 | 2.00 | 0.80 | 0.75 | 3.85 | 6 | 0 | Neurologists’ opinion |
| 576 | Vol_Haepatic_test_Alkaline_phosphatase_fu_RRMS_NO_relapse_exp_1st_year_Teriflunomide | Parameters (L440) | Number of alkaline phosphatase tests experienced RRMS patients for follow-up with no relapse during Teriflunomide treatment 1st year | 220 | 2.00 | 0.80 | 0.75 | 3.85 | 6 | 0 | Neurologists’ opinion |
| 577 | Vol_Haepatic_test_Total_bilirubine_fu_RRMS_NO_relapse_naïve_1st_year_Teriflunomide | Parameters (L441) | Number of total bilirubine tests naïve RRMS patients for follow-up with no relapse during Teriflunomide treatment 1st year | 153 | 2.00 | 0.80 | 0.75 | 3.85 | 6 | 0 | Neurologists’ opinion |
| 578 | Vol_Haepatic_test_Total_bilirubine_fu_RRMS_NO_relapse_exp_1st_year_Teriflunomide | Parameters (L442) | Number of total bilirubine tests experienced RRMS patients for follow-up with no relapse during Teriflunomide treatment 1st year | 220 | 2.00 | 0.80 | 0.75 | 3.85 | 6 | 0 | Neurologists’ opinion |
| 579 | Vol_Kidney_test_Creatinine_fu_RRMS_NO_relapse_naïve_1st_year_Teriflunomide | Parameters (L443) | Number of creatinine tests naïve RRMS patients for follow-up with no relapse during Teriflunomide treatment 1st year | 153 | 2.00 | 0.80 | 0.75 | 3.85 | 6 | 0 | Neurologists’ opinion |
| 580 | Vol_Kidney_test_Creatinine_fu_RRMS_NO_relapse_exp_1st_year_Teriflunomide | Parameters (L444) | Number of creatinine tests experienced RRMS patients for follow-up with no relapse during Teriflunomide treatment 1st year | 220 | 2.00 | 0.80 | 0.75 | 3.85 | 6 | 0 | Neurologists’ opinion |
| 581 | Vol_Sierology_test_HBV_fu_RRMS_NO_relapse_naïve_1st_year_Teriflunomide | Parameters (L445) | Number of HBV tests naïve RRMS patients for follow-up with no relapse during Teriflunomide treatment 1st year | 153 | 2.00 | 0.80 | 0.75 | 3.85 | 6 | 0 | Neurologists’ opinion |
| 582 | Vol_Sierology_test_HBV_fu_RRMS_NO_relapse_exp_1st_year_Teriflunomide | Parameters (L446) | Number of HBV tests experienced RRMS patients for follow-up with no relapse during Teriflunomide treatment 1st year | 220 | 2.00 | 0.80 | 0.75 | 3.85 | 6 | 0 | Neurologists’ opinion |

^a^ Gamma distribution represents uncertainty in resource consumption ([SI1.]).

^b^ If the analytical calculation of SE was unfeasible, a CV=40% of the sample mean has been imposed ([SI1.], [SI14.]).

**Table SI43 – Materials and Methods - Parameters with statistical distribution for base case analysis, OWSA and PSA**

|  |  |  |  |  |  |  |  |  | **Parameters Gamma distribution per PSA^a^** | |  |
| --- | --- | --- | --- | --- | --- | --- | --- | --- | --- | --- | --- |
| **Parameter number** | **Parameter**  **name** | **Spreadsheet**  **(point estimate cell)** | **Parameter**  **description** | **Observations** | **Mean** | **SE^b^** | **LL 95% CI** | **UL 95% CI** | **Alfa** | **Beta** | **Source** |
| **RRMS naïve and experienced patients on Teriflunomide** | | | | | | | | | | | |
| **Healthcare resources - RRMS no relapse - Follow-up** | | | | | | | | | | | |
| 583 | Vol_Sierology_test_HCV_fu_RRMS_NO_relapse_naïve_1st_year_Teriflunomide | Parameters (L447) | Number of HCV tests naïve RRMS patients for follow-up with no relapse during Teriflunomide treatment 1st year | 153 | 2.00 | 0.80 | 0.75 | 3.85 | 6 | 0 | Neurologists’ opinion |
| 584 | Vol_Sierology_test_HCV_fu_RRMS_NO_relapse_exp_1st_year_Teriflunomide | Parameters (L448) | Number of HCV tests experienced RRMS patients for follow-up with no relapse during Teriflunomide treatment 1st year | 220 | 2.00 | 0.80 | 0.75 | 3.85 | 6 | 0 | Neurologists’ opinion |
| 585 | Vol_Sierology_test_HIV_fu_RRMS_NO_relapse_naïve_1st_year_Teriflunomide | Parameters (L449) | Number of HIV tests naïve RRMS patients for follow-up with no relapse during Teriflunomide treatment 1st year | 153 | 2.00 | 0.80 | 0.75 | 3.85 | 6 | 0 | Neurologists’ opinion |
| 586 | Vol_Sierology_test_HIV_fu_RRMS_NO_relapse_exp_1st_year_Teriflunomide | Parameters (L450) | Number of HIV tests experienced RRMS patients for follow-up with no relapse during Teriflunomide treatment 1st year | 220 | 2.00 | 0.80 | 0.75 | 3.85 | 6 | 0 | Neurologists’ opinion |
| 587 | Vol_Sierology_test_quantiferon_fu_RRMS_NO_relapse_naïve_1st_year_Teriflunomide | Parameters (L451) | Number of quantiferon tests naïve RRMS patients for follow-up with no relapse during Teriflunomide treatment 1st year | 153 | 2.00 | 0.80 | 0.75 | 3.85 | 6 | 0 | Neurologists’ opinion |
| 588 | Vol_Sierology_test_quantiferon_fu_RRMS_NO_relapse_exp_1st_year_Teriflunomide | Parameters (L452) | Number of quantiferon tests experienced RRMS patients for follow-up with no relapse during Teriflunomide treatment 1st year | 220 | 2.00 | 0.80 | 0.75 | 3.85 | 6 | 0 | Neurologists’ opinion |
| 589 | Vol_Sierology_test_zooster_fu_RRMS_NO_relapse_naïve_1st_year_Teriflunomide | Parameters (L453) | Number of zooster tests naïve RRMS patients for follow-up with no relapse during Teriflunomide treatment 1st year | 153 | 2.00 | 0.80 | 0.75 | 3.85 | 6 | 0 | Neurologists’ opinion |
| 590 | Vol_Sierology_test_zooster_fu_RRMS_NO_relapse_exp_1st_year_Teriflunomide | Parameters (L454) | Number of zooster tests experienced RRMS patients for follow-up with no relapse during Teriflunomide treatment 1st year | 220 | 2.00 | 0.80 | 0.75 | 3.85 | 6 | 0 | Neurologists’ opinion |
| 591 | Vol_Physiology_test_vitamin_D_fu_RRMS_NO_relapse_naïve_1st_year_Teriflunomide | Parameters (L455) | Number of vitamin D tests naïve RRMS patients for follow-up with no relapse during Teriflunomide treatment 1st year | 153 | 1.00 | 0.40 | 0.38 | 1.92 | 6 | 0 | Neurologists’ opinion |
| 592 | Vol_Physiology_test_vitamin_D_fu_RRMS_NO_relapse_exp_1st_year_Teriflunomide | Parameters (L456) | Number of vitamin D tests experienced RRMS patients for follow-up with no relapse during Teriflunomide treatment 1st year | 220 | 1.00 | 0.40 | 0.38 | 1.92 | 6 | 0 | Neurologists’ opinion |
| 593 | Vol_Neurologist_visit_fu_RRMS_NO_relapse_naïve_1st_year_Teriflunomide | Parameters (L457) | Number of neurologist visits naïve RRMS patients for follow-up with no relapse during Teriflunomide treatment 1st year | 153 | 1.16 | 0.46 | 0.43 | 2.21 | 6 | 0 | Neurologists’ opinion |
| 594 | Vol_Neurologist_visit_fu_RRMS_NO_relapse_exp_1st_year_Teriflunomide | Parameters (L458) | Number of neurologist visists experienced RRMS patients for follow-up with no relapse during Teriflunomide treatment 1st year | 220 | 3.00 | 1.20 | 0.43 | 2.22 | 6 | 0 | Neurologists’ opinion |

^a^ Gamma distribution represents uncertainty in resource consumption ([SI1.]).

^b^ If the analytical calculation of SE was unfeasible, a CV=40% of the sample mean has been imposed ([SI1.], [SI14.]).

**Table SI44 – Materials and Methods - Parameters with statistical distribution for base case analysis, OWSA and PSA**

|  |  |  |  |  |  |  |  |  | **Parameters Gamma distribution per PSA^a^** | |  |
| --- | --- | --- | --- | --- | --- | --- | --- | --- | --- | --- | --- |
| **Parameter number** | **Parameter**  **name** | **Spreadsheet**  **(point estimate cell)** | **Parameter**  **description** | **Observations** | **Mean** | **SE^b^** | **LL 95% CI** | **UL 95% CI** | **Alfa** | **Beta** | **Source** |
| **RRMS naïve and experienced patients on Teriflunomide** | | | | | | | | | | | |
| **Healthcare resources - RRMS no relapse - Follow-up** | | | | | | | | | | | |
| 595 | Vol_Brain_MRI_NOC_fu_RRMS_NO_relapse_ naïve _2_7_year | Parameters (L462) | Number of brain MRIs without contrast naïve RRMS patients for follow-up with no relapse during Teriflunomide treatment years 2-7 | 153 | 0.90 | 0.36 | 0.34 | 1.73 | 6 | 0 | Neurologists’ opinion |
| 596 | Vol_Brain_MRI_NOC_fu_RRMS_NO_relapse_exp_2_7_year | Parameters (L463) | Number of brain MRIs without contrast experienced RRMS patients for follow-up with no relapse during Teriflunomide treatment years 2-7 | 220 | 0.90 | 0.36 | 0.34 | 1.73 | 6 | 0 | Neurologists’ opinion |
| 597 | Vol_Brain_MRI_C_fu_RRMS_NO_relapse_naïve_2_7_year | Parameters (L464) | Number of brain MRIs with contrast naïve RRMS patients for follow-up with no relapse during Teriflunomide treatment years 2-7 | 153 | 2.50 | 1.00 | 0.94 | 4.81 | 6 | 0 | Neurologists’ opinion |
| 598 | Vol_Brain_MRI_C_fu_RRMS_NO_relapse_exp_2_7_year | Parameters (L465) | Number of brain MRIs with contrast experienced RRMS patients for follow-up with no relapse during Teriflunomide treatment years 2-7 | 220 | 1.00 | 0.40 | 0.38 | 1.92 | 6 | 0 | Neurologists’ opinion |
| 599 | Vol_full_blood_test_fu_RRMS_NO_relapse_naïve_2_7_year | Parameters (L469) | Number of full blood tests naïve RRMS patients for follow-up with no relapse during Teriflunomide treatment years 2-7 | 153 | 1.41 | 0.57 | 0.53 | 2.72 | 6 | 0 | Neurologists’ opinion |
| 600 | Vol_full_blood_test_fu_RRMS_NO_relapse_exp_2_7_year | Parameters (L470) | Number of full blood tests experienced RRMS patients for follow-up with no relapse during Teriflunomide treatment years 2-7 | 220 | 3.00 | 1.20 | 1.13 | 5.77 | 6 | 0 | Neurologists’ opinion |
| 601 | Vol_Haepatic_test_ALT_fu_RRMS_NO_relapse_naïve_2_7_year Teriflunomide | Parameters (L471) | Number of ALT tests naïve RRMS patients for follow-up with no relapse during Teriflunomide treatment years 2-7 | 153 | 3.00 | 1.20 | 1.13 | 5.77 | 6 | 0 | Neurologists’ opinion |
| 602 | Vol_Haepatic_test_ALT_fu_RRMS_NO_relapse_exp_2_7_year Teriflunomide | Parameters (L472) | Number of ALT tests experienced RRMS patients for follow-up with no relapse during Teriflunomide treatment years 2-7 | 220 | 3.00 | 1.20 | 1.13 | 5.77 | 6 | 0 | Neurologists’ opinion |
| 603 | Vol_Haepatic_test_AST_fu_RRMS_NO_relapse_naïve_2_7_year Teriflunomide | Parameters (L473) | Number of AST tests naïve RRMS patients for follow-up with no relapse during Teriflunomide treatment years 2-7 | 153 | 3.00 | 1.20 | 1.13 | 5.77 | 6 | 0 | Neurologists’ opinion |
| 604 | Vol_Haepatic_test_AST_fu_RRMS_NO_relapse_exp_2_7_year Teriflunomide | Parameters (L474) | Number of AST tests experienced RRMS patients for follow-up with no relapse during Teriflunomide treatment years 2-7 | 220 | 3.00 | 1.20 | 1.13 | 5.77 | 6 | 0 | Neurologists’ opinion |
| 605 | Vol_Haepatic_test_gGT_fu_RRMS_NO_relapse_naïve_2_7_year Teriflunomide | Parameters (L475) | Number of gGT tests naïve RRMS patients for follow-up with no relapse during Teriflunomide treatment years 2-7 | 153 | 3.00 | 1.20 | 1.13 | 5.77 | 6 | 0 | Neurologists’ opinion |
| 606 | Vol_Haepatic_test_gGT _fu_RRMS_NO_relapse_exp_2_7_year Teriflunomide | Parameters (L476) | Number of gGT tests experienced RRMS patients for follow-up with no relapse during Teriflunomide treatment years 2-7 | 220 | 3.00 | 1.20 | 1.13 | 5.77 | 6 | 0 | Neurologists’ opinion |

^a^ Gamma distribution represents uncertainty in resource consumption ([SI1.]).

^b^ If the analytical calculation of SE was unfeasible, a CV=40% of the sample mean has been imposed ([SI1.], [SI14.]).

**Table SI45 – Materials and Methods - Parameters with statistical distribution for base case analysis, OWSA and PSA**

|  |  |  |  |  |  |  |  |  | **Parameters Gamma distribution per PSA^a^** | |  |
| --- | --- | --- | --- | --- | --- | --- | --- | --- | --- | --- | --- |
| **Parameter number** | **Parameter**  **name** | **Spreadsheet**  **(point estimate cell)** | **Parameter**  **description** | **Observations** | **Mean** | **SE^b^** | **LL 95% CI** | **UL 95% CI** | **Alfa** | **Beta** | **Source** |
| **RRMS naïve and experienced patients on Teriflunomide** | | | | | | | | | | | |
| **Healthcare resources - RRMS no relapse - Follow-up** | | | | | | | | | | | |
| 607 | Vol_Haepatic_test_Alkaline_phosphatase_fu_RRMS_NO_relapse_naïve_2_7_year Teriflunomide | Parameters (L477) | Number of alkaline phosphatase tests naïve RRMS patients for follow-up with no relapse during Teriflunomide treatment years 2-7 | 153 | 2.00 | 0.80 | 0.75 | 3.85 | 6 | 0 | Neurologists’ opinion |
| 608 | Vol_Haepatic_test_Alkaline_phosphatase_fu_RRMS_NO_relapse_exp_2_7_year Teriflunomide | Parameters (L478) | Number of alkaline phosphatase tests experienced RRMS patients for follow-up with no relapse during Teriflunomide treatment years 2-7 | 220 | 2.00 | 0.80 | 0.75 | 3.85 | 6 | 0 | Neurologists’ opinion |
| 609 | Vol_Haepatic_test_Total_bilirubine_fu_RRMS_NO_relapse_naïve_2_7_year Teriflunomide | Parameters (L479) | Number of total bilirubine tests naïve RRMS patients for follow-up with no relapse during Teriflunomide treatment years 2-7 | 153 | 2.00 | 0.80 | 0.75 | 3.85 | 6 | 0 | Neurologists’ opinion |
| 610 | Vol_Haepatic_test_Total_bilirubine_fu_RRMS_NO_relapse_exp_2_7_year Teriflunomide | Parameters (L480) | Number of total bilirubine tests experienced RRMS patients for follow-up with no relapse during Teriflunomide treatment years 2-7 | 220 | 2.00 | 0.80 | 0.75 | 3.85 | 6 | 0 | Neurologists’ opinion |
| 611 | Vol_Kidney_test_Creatinine_fu_RRMS_NO_relapse_naïve_2_7_Teriflunomide | Parameters (L481) | Number of creatinine tests naïve RRMS patients for follow-up with no relapse during Teriflunomide treatment years 2-7 | 153 | 3.00 | 1.20 | 1.13 | 5.77 | 6 | 0 | Neurologists’ opinion |
| 612 | Vol_Kidney_test_Creatinine_fu_RRMS_NO_relapse_exp_2_7_Teriflunomide | Parameters (L482) | Number of creatinine tests experienced RRMS patients for follow-up with no relapse during Teriflunomide treatment years 2-7 | 220 | 3.00 | 1.20 | 1.13 | 5.77 | 6 | 0 | Neurologists’ opinion |
| 613 | Vol_Sierology_test_HBV_fu_RRMS_NO_relapse_naïve_2_7_year_Teriflunomide | Parameters (L483) | Number of HBV tests naïve RRMS patients for follow-up with no relapse during Teriflunomide treatment years 2-7 | 153 | 3.00 | 1.20 | 1.13 | 5.77 | 6 | 0 | Neurologists’ opinion |
| 614 | Vol_Sierology_test_HBV_fu_RRMS_NO_relapse_exp_2_7_year_Teriflunomide | Parameters (L484) | Number of HBV tests experienced RRMS patients for follow-up with no relapse during Teriflunomide treatment years 2-7 | 220 | 3.00 | 1.20 | 1.13 | 5.77 | 6 | 0 | Neurologists’ opinion |
| 615 | Vol_Sierology_test_HCV_fu_RRMS_NO_relapse_naïve_2_7_year_Teriflunomide | Parameters (L485) | Number of HCV tests naïve RRMS patients for follow-up with no relapse during Teriflunomide treatment years 2-7 | 153 | 3.00 | 1.20 | 1.13 | 5.77 | 6 | 0 | Neurologists’ opinion |
| 616 | Vol_Sierology_test_HCV_fu_RRMS_NO_relapse_exp_2_7_year_Teriflunomide | Parameters (L486) | Number of HCV tests experienced RRMS patients for follow-up with no relapse during Teriflunomide treatment years 2-7 | 220 | 3.00 | 1.20 | 1.13 | 5.77 | 6 | 0 | Neurologists’ opinion |
| 617 | Vol_Sierology_test_HIV_fu_RRMS_NO_relapse_naïve_2_7_year_Teriflunomide | Parameters (L487) | Number of HIV tests naïve RRMS patients for follow-up with no relapse during Teriflunomide treatment years 2-7 | 153 | 3.00 | 1.20 | 1.13 | 5.77 | 6 | 0 | Neurologists’ opinion |
| 618 | Vol_Sierology_test_HIV_fu_RRMS_NO_relapse_exp_2_7_year_Teriflunomide | Parameters (L488) | Number of HIV tests experienced RRMS patients for follow-up with no relapse during Teriflunomide treatment years 2-7 | 220 | 3.00 | 1.20 | 1.13 | 5.77 | 6 | 0 | Neurologists’ opinion |

^a^ Gamma distribution represents uncertainty in resource consumption ([SI1.]).

^b^ If the analytical calculation of SE was unfeasible, a CV=40% of the sample mean has been imposed ([SI1.], [SI14.]).

**Table SI46 – Materials and Methods - Parameters with statistical distribution for base case analysis, OWSA and PSA**

|  |  |  |  |  |  |  |  |  | **Parameters Gamma distribution per PSA^a^** | |  |
| --- | --- | --- | --- | --- | --- | --- | --- | --- | --- | --- | --- |
| **Parameter number** | **Parameter**  **name** | **Spreadsheet**  **(point estimate cell)** | **Parameter**  **description** | **Observations** | **Mean** | **SE^b^** | **LL 95% CI** | **UL 95% CI** | **Alfa** | **Beta** | **Source** |
| **RRMS naïve and experienced patients on Teriflunomide** | | | | | | | | | | | |
| **Healthcare resources - RRMS no relapse - Follow-up** | | | | | | | | | | | |
| 619 | Vol_Sierology_test_quantiferon_fu_RRMS_NO_relapse_naïve_2_7_year_Teriflunomide | Parameters (L489) | Number of quantiferon tests naïve RRMS patients for follow-up with no relapse during Teriflunomide treatment years 2-7 | 153 | 3.00 | 1.20 | 1.13 | 5.77 | 6 | 0 | Neurologists’ opinion |
| 620 | Vol_Sierology_test_quantiferon_fu_RRMS_NO_relapse_exp_2_7_year_Teriflunomide | Parameters (L490) | Number of quantiferon tests experienced RRMS patients for follow-up with no relapse during Teriflunomide treatment years 2-7 | 220 | 3.00 | 1.20 | 1.13 | 5.77 | 6 | 0 | Neurologists’ opinion |
| 621 | Vol_Sierology_test_zooster_fu_RRMS_NO_relapse_naïve_2_7_year_Teriflunomide | Parameters (L491) | Number of zooster tests naïve RRMS patients for follow-up with no relapse during Teriflunomide treatment years 2-7 | 153 | 3.00 | 1.20 | 1.13 | 5.77 | 6 | 0 | Neurologists’ opinion |
| 622 | Vol_Sierology_test_zooster_fu_RRMS_NO_relapse_exp_2_7_year_Teriflunomide | Parameters (L492) | Number of zooster tests experienced RRMS patients for follow-up with no relapse during Teriflunomide treatment years 2-7 | 220 | 3.00 | 1.20 | 1.13 | 5.77 | 6 | 0 | Neurologists’ opinion |
| 623 | Vol_Physiology_test_vitamin_D_fu_RRMS_NO_relapse_naïve_2_7_year_Teriflunomide | Parameters (L493) | Number of vitamin D tests naïve RRMS patients for follow-up with no relapse during Teriflunomide treatment years 2-7 | 153 | 1.00 | 0.40 | 0.38 | 1.92 | 6 | 0 | Neurologists’ opinion |
| 624 | Vol_Physiology_test_vitamin_D_fu_RRMS_NO_relapse_exp_2_7_year_Teriflunomide | Parameters (L494) | Number of vitamin D tests experienced RRMS patients for follow-up with no relapse during Teriflunomide treatment years 2-7 | 220 | 1.00 | 0.40 | 0.38 | 1.92 | 6 | 0 | Neurologists’ opinion |
| 625 | Vol_Neurologist_visit_fu_RRMS_NO_relapse_naïve_2_7_year_Teriflunomide | Parameters (L495) | Number of neurologist visits for naïve RRMS patients for follow-up with no relapse during Teriflunomide treatment years 2-7 | 153 | 2.00 | 0.80 | 0.75 | 3.85 | 6 | 0 | Neurologists’ opinion |
| 626 | Vol_Neurologist_visit_fu_RRMS_NO_relapse_exp _2_7_year_Teriflunomide | Parameters (L496) | Number of neurologist visits for experienced RRMS patients for follow-up with no relapse during Teriflunomide treatment years 2-7 | 220 | 2.00 | 0.80 | 0.75 | 3.85 | 6 | 0 | Neurologists’ opinion |
| **Healthcare resources - RRMS relapse - Follow-up** | | | | | | | | | | | |
| 627 | Vol_Brain_MRI_fu_RRMS_relapse_1st_year_Teriflunomide | Parameters (L527) | Number of brain MRIs with contrast for both naïve and experienced RRMS patients for follow-up with relapse during Teriflunomide treatment 1st year | 373 | 2.00 | 0.80 | 0.75 | 3.85 | 6 | 0 | Neurologists’ opinion |
| 628 | Vol_Neurologist_visit_fu_RRMS_relapse_1st_year_Teriflunomide | Parameters (L529) | Number of neurologist visits for both naïve and experienced RRMS patients for follow-up with relapse during Teriflunomide treatment 1st year | 373 | 1.50 | 0.60 | 0.56 | 2.89 | 6 | 0 | Neurologists’ opinion |
| 629 | Vol_Blood_test_fu_RRMS_relapse_2_7_year_Teriflunomide | Parameters (L530) | Number of full blood tests for both naïve and experienced RRMS patients for follow-up with relapse during Teriflunomide treatment years 2-7 | 373 | 4.00 | 1.60 | 1.51 | 7.69 | 6 | 1 | Neurologists’ opinion |

^a^ Gamma distribution represents uncertainty in resource consumption ([SI1.]).

^b^ If the analytical calculation of SE was unfeasible, a CV=40% of the sample mean has been imposed ([SI1.], [SI14.]).

**Table SI47 – Materials and Methods - Parameters with statistical distribution for base case analysis, OWSA and PSA**

|  |  |  |  |  |  |  |  |  | **Parameters Gamma distribution per PSA^a^** | |  |
| --- | --- | --- | --- | --- | --- | --- | --- | --- | --- | --- | --- |
| **Parameter number** | **Parameter**  **name** | **Spreadsheet**  **(point estimate cell)** | **Parameter**  **description** | **Observations** | **Mean** | **SE^b^** | **LL 95% CI** | **UL 95% CI** | **Alfa** | **Beta** | **Source** |
| **RRMS naïve and experienced patients on Teriflunomide** | | | | | | | | | | | |
| **Healthcare resources - RRMS relapse - Follow-up** | | | | | | | | | | | |
| 630 | Vol_Kidney_test_Creatinine_fu_RRMS_relapse_2_7_year_Teriflunomide | Parameters (L531) | Number of creatinine tests for both naïve and experienced RRMS patients for follow-up with relapse during Teriflunomide treatment years 2-7 | 373 | 4.00 | 1.60 | 1.51 | 7.69 | 6 | 1 | Neurologists’ opinion |
| 631 | Vol_Haepatic_test_ALT_fu_RRMS_relapse_2_7_year_Teriflunomide | Parameters (L532) | Number of ALT tests for both naïve and experienced RRMS patients for follow-up with relapse during Teriflunomide treatment years 2-7 | 373 | 4.00 | 1.60 | 1.51 | 7.69 | 6 | 1 | Neurologists’ opinion |
| 632 | Vol_Haepatic_test_AST_fu_RRMS_relapse_2_7_year_Teriflunomide | Parameters (L533) | Number of AST tests for both naïve and experienced RRMS patients for follow-up with relapse during Teriflunomide treatment years 2-7 | 373 | 4.00 | 1.60 | 1.51 | 7.69 | 6 | 1 | Neurologists’ opinion |
| 633 | Vol_Haepatic_test_gGT_fu_RRMS_relapse_2_7_year_Teriflunomide | Parameters (L534) | Number of gGT tests for both naïve and experienced RRMS patients for follow-up with relapse during Teriflunomide treatment years 2-7 | 373 | 4.00 | 1.60 | 1.51 | 7.69 | 6 | 1 | Neurologists’ opinion |
| 634 | Vol_Neurologist_visit_fu_RRMS_relapse_2_7_year_Teriflunomide | Parameters (L535) | Number of neurologist visits for both naïve and experienced RRMS patients for follow-up with relapse during Teriflunomide treatment years 2-7 | 373 | 1.50 | 0.60 | 0.56 | 2.89 | 6 | 0 | Neurologists’ opinion |
| 635 | Vol_ Brain_MRI _fu_RRMS_relapse_2_7_year_Teriflunomide | Parameters (L536) | Number of brain MRIs with contrast for both naïve and experienced RRMS patients for follow-up with relapse during Teriflunomide treatment years 2-7 | 373 | 2.00 | 0.80 | 0.75 | 3.85 | 6 | 0 | Neurologists’ opinion |
| **Healthcare resources – Adverse events - Teriflunomide** | | | | | | | | | | | |
| 636 | Therapy_dur_hair_thinning_1st_year_only_Teriflunomide | Parameters (L540) | Threrapy duration (days) for hair thinning for both naïve and experienced RRMS patients for adverse events during Teriflunomide treatment 1st year | 373 | 91.31 | 36.53 | 34.37 | 175.60 | 6 | 15 | Neurologists’ opinion |
| 637 | Therapy_dur_rachialgy_1st_year_only_Teriflunomide | Parameters (L542) | Threrapy duration (days) for rachialgy for both naïve and experienced RRMS patients for adverse events during Teriflunomide treatment 1st year | 373 | 7.02 | 2.81 | 2.64 | 13.51 | 6 | 1 | Neurologists’ opinion |

^a^ Gamma distribution represents uncertainty in resource consumption ([SI1.]).

^b^ If the analytical calculation of SE was unfeasible, a CV=40% of the sample mean has been imposed ([SI1.], [SI14.]).

**Table SI48 – Materials and Methods - Parameters with statistical distribution for base case analysis, OWSA and PSA**

|  |  |  |  |  |  |  |  |  | **Parameters Gamma distribution per PSA^a^** | |  |
| --- | --- | --- | --- | --- | --- | --- | --- | --- | --- | --- | --- |
| **Parameter number** | **Parameter**  **name** | **Spreadsheet**  **(point estimate cell)** | **Parameter**  **description** | **Observations** | **Mean** | **SE^b^** | **LL 95% CI** | **UL 95%**  **CI** | **Alfa** | **Beta** | **Source** |
| **RRMS naïve and experienced patients on Teriflunomide** | | | | | | | | | | | |
| **Healthcare resources – Adverse events - Teriflunomide** | | | | | | | | | | | |
| 638 | Therapy_dur_rachialgy_2_7_year_Teriflunomide | Parameters (L544) | Threrapy duration (days) for rachialgy for both naïve and experienced RRMS patients for adverse events during Teriflunomide treatment years 2-7 | 373 | 7.02 | 2.81 | 2.64 | 13.51 | 6 | 1 | Neurologists’ opinion |
| 639 | Therapy_dur_haepatoprotection_1st_year_only_Teriflunomide | Parameters (L560) | Threrapy duration (days) for haepatoprotection for both naïve and experienced RRMS patients for adverse events during Teriflunomide treatment 1st year | 373 | 186.63 | 73.05 | 68.73 | 351.20 | 6 | 29 | Neurologists’ opinion |
| 640 | Therapy_dur_haepatoprotection_2_7_year_Teriflunomide | Parameters (L562) | Threrapy duration (days) for haepatoprotection for both naïve and experienced RRMS patients for adverse events during Teriflunomide treatment years 2-7 | 373 | 186.63 | 73.05 | 68.73 | 351.20 | 6 | 29 | Neurologists’ opinion |
| 641 | Therapy_dur_infection_1st_year_only_Teriflunomide | Parameters (L578) | Threrapy duration (days) for infection for both naïve and experienced RRMS patients for adverse events during Teriflunomide treatment 1st year | 373 | 10.03 | 4.01 | 3.78 | 19.30 | 6 | 2 | Neurologists’ opinion |
| 642 | Therapy_dur_infection_2_7_year_Teriflunomide | Parameters (L580) | Threrapy duration (days) for infection for both naïve and experienced RRMS patients for adverse events during Teriflunomide treatment years 2-7 | 373 | 10.03 | 4.01 | 3.78 | 19.30 | 6 | 2 | Neurologists’ opinion |
| **Non-healthcare resources – Volume** | | | | | | | | | | | |
| 643 | Vol_Caregivers_1st_year_naïve | Parameters (L691) | Number of caregivers for naïve RRMS patients during Teriflunomide treatment 1st year | 153 | 1.00 | 0.40 | 0.38 | 1.92 | 6 | 0 | Neurologists’ opinion |
| 644 | Vol_Caregivers_1st_year_exp | Parameters (L692) | Number of caregivers for experienced RRMS patients during Teriflunomide treatment 1st year | 220 | 1.00 | 0.40 | 0.38 | 1.92 | 6 | 0 | Neurologists’ opinion |
| 645 | Vol_Caregivers_2_7_year_naïve | Parameters (L693) | Number of caregivers for naïve RRMS patients during Teriflunomide treatment years 2-7 | 153 | 1.13 | 0.45 | 0.42 | 2.16 | 6 | 0 | Neurologists’ opinion |
| 646 | Vol_Caregivers_2_7_year_exp | Parameters (L694) | Number of caregivers for experienced RRMS patients during Teriflunomide treatment years 2-7 | 220 | 1.13 | 0.45 | 0.42 | 2.16 | 6 | 0 | Neurologists’ opinion |
| 647 | Km_from_home_to_go_naïve | Parameters (L695) | Kilometers from home to hospital (one-way trip) for naïve RRMS patients during Teriflunomide treatment years 1-7 | 153 | 58.75 | 23.50 | 22.11 | 112.98 | 6 | 9 | Neurologists’ opinion |
| 648 | Km_from_home_to_go_exp | Parameters (L696) | Kilometers from home to hospital (one-way trip) for experienced RRMS patients during Teriflunomide treatment years 1-7 | 153 | 56.25 | 22.50 | 21.17 | 108.17 | 6 | 9 | Neurologists’ opinion |
| 649 | Minutes_Transportation_naïve | Parameters (L697) | Minutes transportation from home to hospital (one-way trip) for naïve RRMS patients during Teriflunomide treatment years 1-7 | 153 | 70.50 | 28.20 | 26.53 | 135.58 | 6 | 11 | Neurologists’ opinion |
| 650 | Minutes_Transportation_exp | Parameters (L698) | Minutes transportation from home to hospital (one-way trip) for experienced RRMS patients during Teriflunomide treatment years 1-7 | 220 | 67.50 | 27.00 | 25.40 | 129.81 | 6 | 11 | Neurologists’ opinion |
| 651 | Pkg_minutes | Parameters (L699) | Parking time for both naïve and experienced RRMS patients during Teriflunomide treatment years 1-7 | 373 | 30.00 | 12.00 | 11.29 | 57.69 | 6 | 5 | Neurologists’ opinion |

^a^ Gamma distribution represents uncertainty in resource consumption ([SI1.]).

^b^ If the analytical calculation of SE was unfeasible, a CV=40% of the sample mean has been imposed ([SI1.], [SI14.]).

**Table SI49 – Materials and Methods - Parameters with statistical distribution for base case analysis, OWSA and PSA**

|  |  |  |  |  |  |  |  |  | **Parameters Gamma distribution per PSA^a^** | |  |
| --- | --- | --- | --- | --- | --- | --- | --- | --- | --- | --- | --- |
| **Parameter number** | **Parameter**  **name** | **Spreadsheet**  **(point estimate cell)** | **Parameter**  **description** | **Observations** | **Mean** | **SE^b^** | **LL 95% CI** | **UL 95% CI** | **Alfa** | **Beta** | **Source** |
| **RRMS naïve and experienced patients on Teriflunomide** | | | | | | | | | | | |
| **Patient and caregiver's time^a^** | | | | | | | | | | | |
| 652 | Vol_min_adm_patient_Natalizumab_given_fail_naïve_2_7_year_Teriflunomide | Parameters  (L702) | Number minutes patient time administration Natalizumab given poor response to Teriflunomide naïve patients years 2_7 | 15 | 100.40 | 40.00 | 37.63 | 192.31 | 6 | 16 | Neurologists’ opinion |
| 653 | Add_time_for_health_care_serv_min | Parameters (L705) | Number minutes patient additional time for receiving healthcare services during Teriflunomide treatment years 1-7 | 373 | 30.00 | 12.00 | 11.29 | 57.69 | 6 | 5 | Neurologists’ opinion |
| **EDSS score** | | | | | | | | | | | |
| 654 | EDSS_naïve_1st_year | Parameters (L712) | EDSS for naïve RRMS patients during Teriflunomide treatment 1st year | 153 | 2.50 | 1.00 | 1.00 | 5.00 | 6 | 0 | Neurologists’ opinion |
| 655 | EDSS_exp_1st_year | Parameters (L713) | EDSS for experienced RRMS patients during Teriflunomide treatment 1st year | 220 | 3.50 | 1.40 | 1.50 | 6.50 | 6 | 1 | Neurologists’ opinion |
| 656 | EDSS_naïve_2nd_year | Parameters (L714) | EDSS for naïve RRMS patients during Teriflunomide treatment 2nd year | 153 | 3.00 | 1.20 | 1.00 | 6.00 | 6 | 0 | Neurologists’ opinion |
| 657 | EDSS_exp_2nd_year | Parameters (L715) | EDSS for experienced RRMS patients during Teriflunomide treatment 2nd year | 220 | 4.00 | 1.60 | 1.50 | 7.50 | 6 | 1 | Neurologists’ opinion |
| 658 | EDSS_naïve_3rd_year | Parameters (L716) | EDSS for naïve RRMS patients during Teriflunomide treatment 3rd year | 153 | 3.00 | 1.20 | 1.00 | 6.00 | 6 | 0 | Neurologists’ opinion |
| 659 | EDSS_exp_3rd_year | Parameters (L717) | EDSS for experienced RRMS patients during Teriflunomide treatment 3rd year | 220 | 4.50 | 1.80 | 1.50 | 8.50 | 6 | 1 | Neurologists’ opinion |
| 660 | EDSS_naïve_4th_year | Parameters (L718) | EDSS for naïve RRMS patients during Teriflunomide treatment 4th year | 153 | 3.00 | 1.20 | 1.00 | 6.00 | 6 | 0 | Neurologists’ opinion |
| 661 | EDSS_exp_4th_year | Parameters (L719) | EDSS for experienced RRMS patients during Teriflunomide treatment 4th year | 220 | 5.00 | 2.00 | 2.00 | 9.50 | 6 | 1 | Neurologists’ opinion |
| 662 | EDSS_naïve_5th_year | Parameters (L720) | EDSS for naïve RRMS patients during Teriflunomide treatment 5th year | 153 | 3.50 | 1.40 | 1.50 | 6.50 | 6 | 1 | Neurologists’ opinion |
| 663 | EDSS_exp_5th_year | Parameters (L721) | EDSS for experienced RRMS patients during Teriflunomide treatment 5th year | 220 | 5.50 | 2.20 | 2.00 | 10.00 | 6 | 1 | Neurologists’ opinion |
| 664 | EDSS_naïve_6th_year | Parameters (L722) | EDSS for naïve RRMS patients during Teriflunomide treatment 6th year | 153 | 3.50 | 1.40 | 1.50 | 6.50 | 6 | 1 | Neurologists’ opinion |
| 665 | EDSS_exp_6th_year | Parameters (L723) | EDSS for experienced RRMS patients during Teriflunomide treatment 6th year | 220 | 6.50 | 2.60 | 2.00 | 10.00 | 6 | 1 | Neurologists’ opinion |
| 666 | EDSS_naïve_7th_year | Parameters (L724) | EDSS for naïve RRMS patients during Teriflunomide treatment 7th year | 153 | 4.00 | 1.60 | 1.50 | 7.50 | 6 | 1 | Neurologists’ opinion |
| 667 | EDSS_exp_7th_year | Parameters (L725) | EDSS for experienced RRMS patients during Teriflunomide treatment 7th year | 220 | 7.50 | 3.00 | 3.00 | 10.00 | 6 | 1 | Neurologists’ opinion |

^a^ Gamma distribution represents uncertainty in resource consumption and EDSS score ([SI1.]).

^b^ If the analytical calculation of SE was unfeasible, a CV=40% of the sample mean has been imposed ([SI1.], [SI14.]).

**Table SI50 – Materials and Methods - Parameters with statistical distribution for base case analysis, OWSA and PSA (€2019)**

|  |  |  |  |  |  |  |  | **Parameters Normal distribution per PSA^a^** | |  |
| --- | --- | --- | --- | --- | --- | --- | --- | --- | --- | --- |
| **Parameter number** | **Parameter**  **name** | **Spreadsheet**  **(point estimate cell)** | **Parameter**  **description** | **Mean** | **SE^b^** | **LL**  **95%**  **CI** | **UL**  **95%**  **CI** | **Mean** | **SE** | **Source** |
| **RRMS naïve and experienced patients on Teriflunomide** | | | | | | | | | | |
| **Healthcare resources - Unit and yearly costs** | | | | | | | | | | |
| **Tests and visits** | | | | | | | | | |  |
| 668 | uc_INHS_funded_full_blood_test | Parameters (L756) | Unit cost INHS-funded full blood count in outpatient setting for both naïve and experienced RRMS patients | €3.17 | €0.32 | €2.55 | €3.79 | €3.17 | €0.32 | [SI15.] |
| 669 | uc_INHS_funded_lympho_subpop_test | Parameters (L757) | Unit cost INHS-funded lymphocyte subpopulation in outpatient setting for both naïve and experienced RRMS patients | €17.09 | €1.71 | €13.74 | €20.44 | €17.09 | €1.71 | [SI15.] |
| 670 | uc_INHS_funded_haepatologist_visit | Parameters (L758) | Unit cost INHS-funded haepatologist visit in outpatient setting for both naïve and experienced RRMS patients | €20.66 | €2.07 | €16.61 | € 24.71 | €20.66 | €2.07 | [SI15.] |
| 671 | uc_INHS_funded_infectivologist_visit | Parameters (L759) | Unit cost INHS-funded infectivologist visit in outpatient setting for both naïve and experienced RRMS patients | €20.66 | €2.07 | €16.61 | € 24.71 | €20.66 | €2.07 | [SI15.] |
| 672 | uc_INHS_funded_neurologist_visit | Parameters (L760) | Unit cost INHS-funded neurologist visit in outpatient setting for both naïve and experienced RRMS patients | €20.66 | €2.07 | €16.61 | € 24.71 | €20.66 | €2.07 | [SI15.] |
| 673 | uc_INHS_funded_dermatologist_visit | Parameters (L761) | Unit cost INHS-funded dermatologist visit in outpatient setting for both naïve and experienced RRMS patients | €20.66 | €2.07 | €16.61 | € 24.71 | €20.66 | €2.07 | [SI15.] |
| 674 | uc_INHS_funded_cardiolologist_visit | Parameters (L762) | Unit cost INHS-funded cardiolologist visit in outpatient setting for both naïve and experienced RRMS patients | €20.66 | €2.07 | €16.61 | € 24.71 | €20.66 | €2.07 | [SI15.] |
| 675 | uc_INHS_funded_holter_pressure | Parameters (L763) | Unit cost INHS-funded holter test in outpatient setting infection for both naïve and experienced RRMS patients | €41.32 | €4.13 | €33.22 | € 49.42 | €41.32 | €4.13 | [SI15.] |
| 676 | uc_INHS_funded_echocardio | Parameters (L764) | Unit cost INHS-funded echocardiogram test in outpatient setting both naïve and experienced RRMS patients | €51.65 | €5.17 | €41.53 | €61.77 | €51.65 | €5.17 | [SI15.] |
| 677 | uc_INHS_funded_ECG | Parameters (L765) | Unit cost INHS-funded ECG test in outpatient setting for both naïve and experienced RRMS patients | €11.62 | €1.16 | €9.34 | €13.90 | €11.62 | €1.16 | [SI15.] |
| 678 | uc_INHS_funded_brain_MRI_plus_spinal_w_C | Parameters (L766) | Unit cost INHS-funded brain plus spinal cord MRI without contrast in outpatient setting for both naïve and experienced RRMS patients | €282.38 | €28.24 | €227. 03 | €337.73 | €282.38 | €28.24 | [SI15.] |
| 679 | uc_INHS_funded_brain_MRI_wo_C | Parameters (L767) | Unit cost INHS-funded brain MRI without contrast in outpatient setting for both naïve and experienced RRMS patients | €166.58 | €16.66 | €133.93 | €199.23 | €166.58 | €16.66 | [SI15.] |
| 680 | uc_INHS_funded_brain_MRI_w_C | Parameters (L768) | Unit cost INHS-funded brain MRI with contrast in outpatient setting for both naïve and experienced RRMS patients | €166.58 | €16.66 | €133.93 | €199.23 | €166.58 | €16.66 | [SI15.] |
| 681 | uc_INHS_funded_haepatic_ultrasound | Parameters (L769) | Unit cost INHS-funded haepatic ultrasound in outpatient setting for both naïve and experienced RRMS patients | €43.90 | €4.39 | €133.93 | €35.30 | €52.50 | €4.39 | [SI15.] |
| 682 | uc_INHS_funded_OTC | Parameters (L770) | Unit cost INHS-funded hapatic ultrasound in outpatient setting for both naïve and experienced RRMS patients | €35.30 | €3.53 | €28.38 | €42.22 | €35.30 | €3.53 | [SI16.] |
| 683 | uc_INHS_funded_haepatic_test_ALT | Parameters (L771) | Unit cost INHS-funded ALT test in outpatient setting for both naïve and experienced RRMS patients | €1.00 | €0.10 | €0.80 | €1.20 | €1.00 | €0.10 | [SI15.] |
| 684 | uc_INHS_funded_haepatic_test_AST | Parameters (L772) | Unit cost INHS-funded AST test in outpatient setting for both naïve and experienced RRMS patients | €1.04 | €0.10 | €0.84 | €1.24 | €1.04 | €0.10 | [SI15.] |
| 685 | uc_INHS_funded_haepatic_test_gGT | Parameters (L773) | Unit cost INHS-funded gGT test in outpatient setting for both naïve and experienced RRMS patients | €1.13 | €0.11 | €0.91 | €1.35 | €1.13 | €0.11 | [SI15.] |
| 686 | uc_INHS_funded_haepatic_test_total_bilirubin | Parameters (L774) | Unit cost INHS-funded total bilirubin test in outpatient setting for both naïve and experienced RRMS patients | €1.13 | €0.11 | €0.91 | €1.35 | €1.13 | €0.11 | [SI15.] |
| 687 | uc_INHS_funded_haepatic_test_direct_bilirubin | Parameters (L775) | Unit cost INHS-funded direct bilirubin test in outpatient setting for both naïve and experienced RRMS patients | €1.41 | €0.14 | €1.13 | €1.69 | €1.41 | €0.14 | [SI15.] |
| 688 | uc_INHS_funded_haepatic_test_indirect_bilirubin | Parameters (L776) | Unit cost INHS-funded indirect bilirubin test in outpatient setting for both naïve and experienced RRMS patients | €1.41 | €0.14 | €1.13 | €1.69 | €1.41 | €0.14 | [SI15.] |
| 689 | uc_INHS_funded_haepatic_test_alkaline_phosphatase | Parameters (L777) | Unit cost INHS-funded alkaline phosphatase test in outpatient setting for both naïve and experienced RRMS patients | €1.04 | €0.10 | €0.84 | €1.24 | €1.04 | €0.10 | [SI15.] |
| 690 | uc_INHS_funded_haepatic_test_albumine | Parameters (L778) | Unit cost INHS-funded albumine test in outpatient setting for both naïve and experienced RRMS patients | €1.42 | €0.14 | €1.14 | €1.70 | €1.42 | €0.14 | [SI15.] |
| 691 | uc_INHS_funded_metabolic_test_blood_sugar | Parameters (L779) | Unit cost INHS-funded blood sugar test in outpatient setting for both naïve and experienced RRMS patients | €1.17 | €0.12 | €0.94 | €1.40 | €1.17 | €0.12 | [SI15.] |
| 692 | uc_INHS_funded_physiology_test_vitamine_D | Parameters (L780) | Unit cost INHS-funded vitamin D test in outpatient setting for both naïve and experienced RRMS patients | €15.86 | €1.59 | €12.75 | €18.97 | €15.86 | €1.59 | [SI15.] |
| 693 | uc_INHS_funded_sierology_test_quantiferon | Parameters (L781) | Unit cost INHS-funded quantiferon test in outpatient setting for both naïve and experienced RRMS patients | €20.59 | €2.06 | €16.55 | €24.63 | €20.59 | €2.06 | [SI15.] |
| 694 | uc_INHS_funded_sierology_test_HBV | Parameters (L782) | Unit cost INHS-funded HBV test in outpatient setting for both naïve and experienced RRMS patients | €10.01 | €1.00 | €8.05 | €11.97 | €10.01 | €1.00 | [SI15.] |

^a^ Normal distribution was fitted to unit costs ([SI1.]).

^b^ If the analytical calculation of SE was unfeasible, a CV=40% of the sample mean has been imposed ([SI1.], [SI14.]).

**Table SI51 – Materials and Methods - Parameters with statistical distribution for base case analysis, OWSA and PSA (€2019)**

|  |  |  |  |  |  |  |  | **Parameters Normal distribution per PSA^a^** | |  |
| --- | --- | --- | --- | --- | --- | --- | --- | --- | --- | --- |
| **Parameter number** | **Parameter**  **name** | **Spreadsheet**  **(point estimate cell)** | **Parameter**  **description** | **Mean** | **SE^b^** | **LL 95% CI** | **UL 95% CI** | **Mean** | **SE** | **Source** |
| **RRMS naïve and experienced patients on Teriflunomide** | | | | | | | | | | |
| **Healthcare resources - Unit and yearly costs** | | | | | | | | | | |
| **Tests and visits** | | | | | | | | | | |
| 695 | uc_INHS_funded_sierology_test_HCV | Parameters (L783) | Unit cost INHS-funded HCV test in outpatient setting for both naïve and experienced RRMS patients | €10.01 | €1.00 | €8.05 | €11.97 | €10.01 | €1.00 | [SI15.] |
| 696 | uc_INHS_funded_sierology_test_HIV | Parameters (L784) | Unit cost INHS-funded HIV test in outpatient setting for both naïve and experienced RRMS patients | €10.90 | €1.09 | €8.05 | €8.76 | €13.04 | €1.09 | [SI15.] |
| 697 | uc_INHS_funded_sierology_test_HPV | Parameters (L785) | Unit cost INHS-funded HPV test in outpatient setting for naïve female RRMS patients | €72.70 | €7.27 | €8.05 | €58.45 | €86.95 | €7.27 | [SI15.] |
| 698 | uc_INHS_funded_sierology_test_zooster | Parameters (L786) | Unit cost INHS-funded zooster test in outpatient setting for both naïve and experienced RRMS patients | €9.41 | €0.94 | €7.57 | €11.25 | €9.41 | €0.94 | [SI15.] |
| 699 | uc_INHS_funded_sierology_test_JCV | Parameters (L787) | Unit cost INHS-funded JCV test in outpatient setting for both naïve and experienced RRMS patients | €63.52 | €6.35 | €51.07 | €75.97 | €63.52 | €6.35 | [SI15.] |
| 700 | uc_INHS_funded_kidney_test_creatinine | Parameters (L788) | Unit cost INHS-funded creatinine test in outpatient setting for both naïve and experienced RRMS patients | €1.13 | €0.11 | €0.91 | €1.35 | €1.13 | €0.11 | [SI15.] |
| 701 | uc_INHS_funded_kidney_test_urea | Parameters (L789) | Unit cost INHS-funded urea test in outpatient setting for both naïve and experienced RRMS patients | €1.13 | €0.11 | €0.91 | €1.35 | €1.13 | €0.11 | [SI15.] |
| 702 | uc_INHS_funded_kidney_test_urine | Parameters (L790) | Unit cost INHS-funded urea test in outpatient setting for both naïve and experienced RRMS patients | €1.13 | €0.11 | €0.91 | €1.35 | €1.13 | €0.11 | [SI15.] |
| **RRMS-related drugs administration setting** | | | | | | | | | |  |
| 703 | uc_INHS_funded_Adm_Outpatient_one_half | Parameters (L799) | One-half unit cost per minute INHS-funded outpatient setting administration for both naïve and experienced RRMS patients | €0.05 | €0.005 | €0.04 | €0.06 | €0.05 | €0.005 | [SI15.] |
| 704 | uc_INHS_funded_Adm_DH_one_half | Parameters (L800) | One-half unit cost per minute INHS-funded DH setting administration for both naïve and experienced RRMS patients | €0.31 | €0.03 | €0.25 | €0.37 | €0.31 | €0.03 | [SI17.] |
| **Non RRMS-related drugs - Administration** | | | | | | | | | |  |
| 705 | uc_Hospital_funded_Physio_Natalizumab_adm | Parameters (L808) | Unit cost (100 ml bag) hospital-funded physiological solution for Natalizumab administration given poor response to Teriflunomide naïve patients years 2_7 | €6.69 | €0.67 | €5.38 | €8.00 | €6.69 | €0.67 | [SI3.] |
| 706 | uc_Hospital_funded_iv_line_Natalizumab_Ocrelizumab_Alemtuzumab_adm | Parameters (L809) | Unit cost hospital-funded iv line for Natalizumab, Ocrelizumab and Alemtuzumab administration given poor response to Teriflunomide naïve patients years 2_7 | €0.59 | €0.06 | €0.47 | €0.71 | €0.59 | €0.06 | [SI18.] |
| 707 | uc_Hospital_funded_iv_needle_Natalizumab_Ocrelizumab_Alemtuzumab_adm | Parameters (L810) | Unit cost hospital-funded needle for Natalizumab, Ocrelizumab and Alemtuzumab administration given poor response to Teriflunomide naïve patients years 2_7 | €0.72 | €0.07 | €0.58 | €0.86 | €0.72 | €0.07 | [SI19.] |

^a^ Normal distribution was fitted to unit costs ([SI1.]).

^b^ If the analytical calculation of SE was unfeasible, a CV=40% of the sample mean has been imposed ([SI1.], [SI14.]).

**Table SI52 – Materials and Methods - Parameters with statistical distribution for base case analysis, OWSA and PSA (€2019)**

|  |  |  |  |  |  |  |  | **Parameters Normal distribution per PSA^a^** | |  |
| --- | --- | --- | --- | --- | --- | --- | --- | --- | --- | --- |
| **Parameter number** | **Parameter**  **name** | **Spreadsheet**  **(point estimate cell)** | **Parameter**  **description** | **Mean** | **SE^b^** | **LL**  **95%**  **CI** | **UL**  **95%**  **CI** | **Mean** | **SE** | **Source** |
| **RRMS naïve and experienced patients on Teriflunomide** | | | | | | | | | | |
| **Healthcare resources - Unit and yearly costs** | | | | | | | | | | |
| **Non RRMS-related drugs - Administration** | | | | | | | | |  |  |
| 708 | uc_OOP_funded_antiseptic_Natalizumab_Ocrelizumab_Alemtuzumab | Parameters (L811) | Unit cost (*per* cubic centimeter) hospital-funded antiseptic for Natalizumab administration given poor response to Teriflunomide naïve patients years 2_7 | €0.01 | €0.001 | €0.01 | €0.02 | €0.01 | €0.001 | [SI3.] |
| 709 | uc_OOP_funded_CVC_Natalizumab_adm | Parameters (L812) | Unit cost hospital-funded CVC for Natalizumab administration given poor response to Teriflunomide naïve patients years 2_7 | €150.88 | €15.09 | €0.47 | €0.71 | €150.88 | €15.09 | [SI20.]; [SI21.] |
| 710 | uc_OOP_funded_ev_gauze_Natalizumab _adm | Parameters (L813) | Unit cost hospital-funded gauze for Natalizumab administration given poor response to Teriflunomide naïve patients years 2_7 | €0.01 | €0.001 | €0.01 | €0.02 | €0.01 | €0.001 | [SI22.] |
| **Non RRMS-related drugs - Postmedication** | | | | | | | | |  |  |
| 711 | uc_OOP_funded_Physio_Natalizumab_Ocrelizumab_Alemtuzumab | Parameters (L814) | Unit cost (*per* 100 milliliters bag) hospital-funded physiological solution for Natalizumab, Ocrelizumab, Alemtuzumab postmedication given poor response to Teriflunomide naïve patients years 2_7 | €0.01 | €0.001 | €0.01 | €0.02 | €0.01 | €0.001 | [SI3.] |
| **Non RRMS-related drugs – Adverse events** | | | | | | | | |  |  |
| 712 | uc_OOP_funded_CP_drug_hair_thinning_per_table | Parameters (L816) | Unit cost (*per* table) OOP-funded drug therapy for hair thinning due to Teriflunomide therapy for both naïve and experienced patients 1st year | €0.77 | €0.08 | €0.62 | €0.92 | €0.77 | €0.08 | [SI23.] |
| **Disability aids** | | | | | | | | |  |  |
| 713 | Yearly_cost_INHS_funded_Mobility_Aids | Parameters (L823) | Yearly cost INHS-funded mobility aids | €5610.20 | €561.02 | €4510.62 | €6709.78 | €5610.20 | €561.02 | [SI24.]; [SI25.] |
| 714 | Yearly_cost_OOP_funded_Mobility_Aids | Parameters (L824) | Yearly cost OOP-funded mobility aids | €3282.08 | €328.21 | €2638.80 | €3925.36 | €3282.08 | €328.21 | [SI24.]; [SI25.] |
| 715 | Yearly_cost_OOP_funded_Non_mobility_Aids | Parameters (L825) | Yearly cost OOP-funded non-mobility aids | €3283.30 | €328.33 | €2639.79 | €3926.82 | €3283.30 | €328.33 | [SI24.]; [SI25.] |
| **Non-Healthcare resources - Unit Cost** | | | | | | | | | |  |
| 716 | uc_OOP_funded_Pkg_per_minute | Parameters (L828) | Unit cost (*per* minute) OOP-funded parking for both naïve and experienced patients years 1_7 | €0.03 | €0.003 | €0.04 | €0.04 | €0.03 | €0.003 | [SI26.] |
| 717 | uc_OOP_funded_Car_per_kn | Parameters (L829) | Unit cost (*per* kilometer) OOP-funded car transportation for both naïve and experienced patients years 1_7 | €0.42 | €0.04 | €0.34 | €0.51 | €0.42 | €0.04 | [SI27.] |
| 718 | uc_OOP_funded_Home_paid_help_minute | Parameters (L830) | Unit cost (*per* minute) OOP-funded housekeeper time for both naïve and experienced patients years 1_7 | €0.18 | €0.02 | €0.14 | €0.21 | €0.18 | €0.02 | [SI28.]; [SI29.]; [SI30.] |
| 719 | uc_minute_Patient_time_naïve | Parameters (L831) | Unit cost (per minute) patient time for naïve patients years 1_7 | €0.38 | €0.04 | €0.31 | €0.46 | €0.38 | €0.02 | [SI25.]; [SI31.]; [SI32.]; [SI21.] |
| 720 | uc_minute_Patient_time_exp | Parameters (L832) | Unit cost (*per* minute) patient time for experienced patients years 1_7 | €0.38 | €0.04 | €0.31 | €0.46 | €0.38 | €0.02 | [SI28.]; [SI31.]; [SI32.]; [SI21.] |
| 721 | uc_minute_Caregive_time | Parameters (L833) | Unit cost (*per* minute) caregiver time for both naïve and experienced patients years 1_7 | €0.22 | €0.02 | €0.18 | €0.27 | €0.22 | €0.02 | [SI28.]; [SI31.]; [SI32.]; [SI21.] |

^a^ Normal distribution was fitted to unit costs ([SI1.]).

^b^ If the analytical calculation of SE was unfeasible, a CV=40% of the sample mean has been imposed ([SI1.], [SI14.]).

**Table SI53 – Results - SSA – Adherence to Teriflunomide (€2019)**

| **Parameter variation** | **Incremental Cost**  **(ΔC)** | **Incremental QALYs**  **(ΔQALYs)** | **ICUR**  **(ΔC/ΔQALYs)^a^** | **Δ vs. base case analysis ICUR** |
| --- | --- | --- | --- | --- |
| **Healthcare sector perspective** | | | |  |
| **Basecase CUA** | | | | |
| Adherence=100% | -€1042.68 | 0.480 | Teriflunomide in RRMS  naïve patients is strongly dominant vs RRMS experienced patients | - |
| **OWSA** |  |  |  |  |
| Adherence=90% | €286.60 | 0.432 | €663.01 | +130.54% |
| Adherence=80% | €1615.89 | 0.384 | €4205.33 | +293.72% |
| Adherence=70% | €2945.18 | 0.336 | €8759.74 | +503.52% |
| Adherence=60% | €4274.47 | 0.288 | €14,832.28 | +783.25% |
| Adherence=50% | €5603.75 | 0.240 | €23,333.85 | +1174.87% |
| Adherence=40% | €6933.04 | 0.192 | €36,086.21 | +1762.31% |
| **Societal perspective** | | | |  |
| **Basecase CUA** | | | | |
| Adherence=100% | -€6782.81 | 0.480 | Teriflunomide in RRMS  naïve patients is strongly dominant vs RRMS experienced patients | - |
| **SSA** |  |  |  |  |
| Adherence=90% | -€5453.52 | 0.432 | Teriflunomide in RRMS  naïve patients is strongly dominant vs RRMS experienced patients | - |
| Adherence=80% | -€4124.24 | 0.384 | Teriflunomide in RRMS  naïve patients is strongly dominant vs RRMS experienced patients | - |
| Adherence=70% | -€2794.95 | 0.336 | Teriflunomide in RRMS  naïve patients is strongly dominant vs RRMS experienced patients | - |
| Adherence=60% | -€1465.66 | 0.288 | Teriflunomide in RRMS  naïve patients is strongly dominant vs RRMS experienced patients | - |
| Adherence=50% | -€136.38 | 0.240 | Teriflunomide in RRMS  naïve patients is strongly dominant vs RRMS experienced patients | - |
| Adherence=40% | €1192.91 | 0.192 | €6209.05 | +327.44% |

^a^ When ICUR>0, Teriflunomide in RRMS naïve patients is at the same time more costly and produces more QALYs vs. Teriflunomide in RRMS experienced patients.

**Table SI54 – Results - SSA – Probability of recovery after RRMS relapse (€2019)**

| **Parameter variation** | **Incremental Cost**  **(ΔC)** | | **Incremental QALYs**  **(ΔQALYs)** | **ICUR**  **(ΔC/ΔQALYs)** | **Δ vs. base case ICUR** |
| --- | --- | --- | --- | --- | --- |
| **Healthcare sector perspective** | | | | |  |
| **Basecase CUA** | | | | | |
| Probability of recovery after RRMS relapse =20% | | -€1042.68 | 0.480 | Teriflunomide in RRMS  naïve patients is strongly dominant vs RRMS experienced patients | - |
| **SSA** | |  |  |  |  |
| Probability of recovery after RRMS relapse =30% | | -€2554.42 | 0.457 | Teriflunomide in RRMS  naïve patients is strongly dominant vs RRMS experienced patients | - |
| Probability of recovery after RRMS relapse =40% | | -€3445.24 | 0.441 | Teriflunomide in RRMS  naïve patients is strongly dominant vs RRMS experienced patients | - |
| Probability of recovery after RRMS relapse =50% | | -€3896.86 | 0.432 | Teriflunomide in RRMS  naïve patients is strongly dominant vs RRMS experienced patients | - |
| Probability of recovery after RRMS relapse =60% | | -€4054.39 | 0.426 | Teriflunomide in RRMS  naïve patients is strongly dominant vs RRMS experienced patients | - |
| Probability of recovery after RRMS relapse =70% | | -€4030.78 | 0.422 | Teriflunomide in RRMS  naïve patients is strongly dominant vs RRMS experienced patients | - |
| Probability of recovery after RRMS relapse =80% | | -€3911.25 | 0.421 | Teriflunomide in RRMS  naïve patients is strongly dominant vs RRMS experienced patients | - |
| **Societal perspective** | | | | |  |
| **Basecase CUA** | | | | | |
| Probability of recovery after RRMS relapse =20% | | -€6782.81 | 0.480 | Teriflunomide in RRMS  naïve patients is strongly dominant vs RRMS experienced patients | - |
| **SSA** |  | |  |  |  |
| Probability of recovery after RRMS relapse =30% | | -€8180.31 | 0.457 | Teriflunomide in RRMS  naïve patients is strongly dominant vs RRMS experienced patients | - |
| Probability of recovery after RRMS relapse =40% | | -€8999.37 | 0.441 | Teriflunomide in RRMS  naïve patients is strongly dominant vs RRMS experienced patients | - |
| Probability of recovery after RRMS relapse =50% | | -€9408.76 | 0.432 | Teriflunomide in RRMS  naïve patients is strongly dominant vs RRMS experienced patients | - |
| Probability of recovery after RRMS relapse =60% | | -€9543.92 | 0.426 | Teriflunomide in RRMS  naïve patients is strongly dominant vs RRMS experienced patients | - |
| Probability of recovery after RRMS relapse =70% | | -€9510.80 | 0.422 | Teriflunomide in RRMS  naïve patients is strongly dominant vs RRMS experienced patients | - |
| Probability of recovery after RRMS relapse =80% | | -€9389.81 | 0.421 | Teriflunomide in RRMS  naïve patients is strongly dominant vs RRMS experienced patients | - |

**Table SI55 – Materials and Methods - Transition probability matrix - RRMS naïve patients hypothetical cohort**

|  | **Unrelapsed**  **stage** | **Relapsed**  **stage** | **Remitted**  **stage** | **Switched relapsed**  **stage** | **Switched remitted**  **stage** | **Death^a^** | **Overall** |
| --- | --- | --- | --- | --- | --- | --- | --- |
| **Year 0** | |  |  |  |  |  |  |
| **From Unrelapsed stage to** | 1.0000 | 0.0000 | 0.0000 | 0.0000 | 0.0000 | 0.0000 | 1.0000 |
| **From Relapsed stage to** | 0.0000 | 0.0000 | 0.0000 | 0.0000 | 0.0000 | 0.0000 | 0.0000 |
| **From Remitted stage to** | 0.0000 | 0.0000 | 0.0000 | 0.0000 | 0.0000 | 0.0000 | 0.0000 |
| **From Switched remitted stage to** | 0.0000 | 0.0000 | 0.0000 | 0.0000 | 0.0000 | 0.0000 | 0.0000 |
| **From Switched relapsed stage to** | 0.0000 | 0.0000 | 0.0000 | 0.0000 | 0.0000 | 0.0000 | 0.0000 |
| **Check** |  |  |  |  |  |  | **1.0000** |
| **Year 1** | |  |  |  |  |  |  |
| **From Unrelapsed stage to** | 0.5392 | 0.4603 | 0.0000 | 0.0000 | 0.0000 | 0.0005 | 1.0000 |
| **From Relapsed stage to** | 0.0000 | 0.0000 | 0.0000 | 0.0000 | 0.0000 | 0.0000 | 0.0000 |
| **From Remitted stage to** | 0.0000 | 0.0000 | 0.0000 | 0.0000 | 0.0000 | 0.0000 | 0.0000 |
| **From Switched remitted stage to** | 0.0000 | 0.0000 | 0.0000 | 0.0000 | 0.0000 | 0.0000 | 0.0000 |
| **From Switched relapsed stage to** | 0.0000 | 0.0000 | 0.0000 | 0.0000 | 0.0000 | 0.0000 | 0.0000 |
| **Check** |  |  |  |  |  |  | **1.0000** |
| **Year 2** |  |  |  |  |  |  |  |
| **From Unrelapsed stage to** | 0.2723 | 0.2669 | 0.0000 | 0.0000 | 0.0000 | 0.0003 | 0.5395 |
| **From Relapsed stage to** | 0.0000 | 0.3223 | 0.0920 | 0.0460 | 0.0000 | 0.0002 | 0.4605 |
| **From Remitted stage to** | 0.0000 | 0.0000 | 0.0000 | 0.0000 | 0.0000 | 0.0000 | 0.0000 |
| **From Switched remitted stage to** | 0.0000 | 0.0000 | 0.0000 | 0.0000 | 0.0000 | 0.0000 | 0.0000 |
| **From Switched relapsed stage to** | 0.0000 | 0.0000 | 0.0000 | 0.0000 | 0.0000 | 0.0000 | 0.0000 |
| **Check** |  |  |  |  |  |  | **1.0000** |
| **Year 3** |  |  |  |  |  |  |  |
| **From Unrelapsed stage to** | 0.2722 | 0.2668 | 0.0000 | 0.0000 | 0.0000 | 0.0003 | 0.5392 |
| **From Relapsed stage to** | 0.0000 | 0.2256 | 0.0645 | 0.0322 | 0.0000 | 0.0002 | 0.3226 |
| **From Remitted stage to** | 0.0000 | 0.0456 | 0.0465 | 0.0000 | 0.0000 | 0.00005 | 0.0922 |
| **From Switched remitted stage to** | 0.0000 | 0.0000 | 0.0000 | 0.0092 | 0.0368 | 0.00002 | 0.0461 |
| **From Switched relapsed stage to** | 0.0000 | 0.0000 | 0.0000 | 0.0000 | 0.0000 | 0.0000 | 0.0000 |
| **Check** |  |  |  |  |  |  | **1.0000** |
| **Year 4** |  |  |  |  |  |  |  |
| **From Unrelapsed stage to** | 0.2720 | 0.2666 | 0.0000 | 0.0000 | 0.0000 | 0.0003 | 0.5390 |
| **From Relapsed stage to** | 0.0000 | 0.2257 | 0.0645 | 0.0323 | 0.0000 | 0.0002 | 0.3227 |
| **From Remitted stage to** | 0.0000 | 0.0456 | 0.0465 | 0.0000 | 0.0000 | 0.0001 | 0.0922 |
| **From Switched remitted stage to** | 0.0000 | 0.0000 | 0.0000 | 0.0074 | 0.0295 | 0.00002 | 0.0369 |
| **From Switched relapsed stage to** | 0.0000 | 0.0000 | 0.0000 | 0.0018 | 0.0074 | 0.00001 | 0.0092 |
| **Check** |  |  |  |  |  |  | **1.0000** |
| **Year 5** |  |  |  |  |  |  |  |
| **From Unrelapsed stage to** | 0.2719 | 0.2665 | 0.0000 | 0.0000 | 0.0000 | 0.0003 | 0.5387 |
| **From Relapsed stage to** | 0.0000 | 0.2258 | 0.0646 | 0.0323 | 0.0000 | 0.0002 | 0.3229 |
| **From Remitted stage to** | 0.0000 | 0.0457 | 0.0465 | 0.0000 | 0.0000 | 0.0001 | 0.0923 |
| **From Switched remitted stage to** | 0.0000 | 0.0000 | 0.0000 | 0.0074 | 0.0295 | 0.00002 | 0.0369 |
| **From Switched relapsed stage to** | 0.0000 | 0.0000 | 0.0000 | 0.0018 | 0.0074 | 0.00001 | 0.0092 |
| **Check** |  |  |  |  |  |  | **1.0000** |
| **Year 6** |  |  |  |  |  |  |  |
| **From Unrelapsed stage to** | 0.2718 | 0.2663 | 0.0000 | 0.0000 | 0.0000 | 0.0004 | 0.5385 |
| **From Relapsed stage to** | 0.0000 | 0.2259 | 0.0646 | 0.0323 | 0.0000 | 0.0002 | 0.3231 |
| **From Remitted stage to** | 0.0000 | 0.0457 | 0.0465 | 0.0000 | 0.0000 | 0.0001 | 0.0923 |
| **From Switched remitted stage to** | 0.0000 | 0.0000 | 0.0000 | 0.0074 | 0.0295 | 0.00003 | 0.0369 |
| **From Switched relapsed stage to** | 0.0000 | 0.0000 | 0.0000 | 0.0018 | 0.0074 | 0.00001 | 0.0092 |
| **Check** |  |  |  |  |  |  | **1.0000** |
| **Year 7** |  |  |  |  |  |  |  |
| **From Unrelapsed stage to** | 0.1776 | 0.3602 | 0.0000 | 0.0000 | 0.0000 | 0.0004 | 0.5382 |
| **From Relapsed stage to** | 0.0000 | 0.2260 | 0.0646 | 0.0323 | 0.0000 | 0.0003 | 0.3232 |
| **From Remitted stage to** | 0.0000 | 0.0619 | 0.0304 | 0.0000 | 0.0000 | 0.0001 | 0.0924 |
| **From Switched remitted stage to** | 0.0000 | 0.0000 | 0.0000 | 0.0074 | 0.0295 | 0.00003 | 0.0369 |
| **From Switched relapsed stage to** | 0.0000 | 0.0000 | 0.0000 | 0.0020 | 0.0072 | 0.00001 | 0.0092 |
| **Check** |  |  |  |  |  |  | **1.0000** |

^a^ The probability of all-cause mortality is gender and age-specific [SI2].

**Table SI56 – Materials and Methods - Transition probability matrix - RRMS experienced patients hypothetical cohort**

|  | **Unrelapsed**  **stage** | **Relapsed**  **stage** | **Remitted**  **stage** | **Death^a^** | **Overall** |
| --- | --- | --- | --- | --- | --- |
| **Year 0** | |  |  |  |  |
| **From Unrelapsed stage to** | 1.0000 | 0.0000 | 0.0000 | 0.0000 | 1.0000 |
| **From Relapsed stage to** | 0.0000 | 0.0000 | 0.0000 | 0.0000 | 0.0000 |
| **From Remitted stage to** | 0.0000 | 0.0000 | 0.0000 | 0.0000 | 0.0000 |
| **Check** |  |  |  |  | **1.0000** |
| **Year 1** | |  |  |  |  |
| **From Unrelapsed stage to** | 0.5044 | 0.4951 | 0.0000 | 0.0005 | 1.0000 |
| **From Relapsed stage to** | 0.0000 | 0.0000 | 0.0000 | 0.0000 | 0.0000 |
| **From Remitted stage to** | 0.0000 | 0.0000 | 0.0000 | 0.0000 | 0.0000 |
| **Check** |  |  |  |  | **1.0000** |
| **Year 2** |  |  |  |  |  |
| **From Unrelapsed stage to** | 0.1664 | 0.3380 | 0.0000 | 0.0002 | 0.5047 |
| **From Relapsed stage to** | 0.0000 | 0.3960 | 0.0990 | 0.0002 | 0.4953 |
| **From Remitted stage to** | 0.0000 | 0.0000 | 0.0000 | 0.0000 | 0.0000 |
| **Check** |  |  |  |  | **1.0000** |
| **Year 3** |  |  |  |  |  |
| **From Unrelapsed stage to** | 0.1663 | 0.3378 | 0.0000 | 0.0003 | 0.5044 |
| **From Relapsed stage to** | 0.0000 | 0.2773 | 0.0694 | 0.0002 | 0.3469 |
| **From Remitted stage to** | 0.0000 | 0.0996 | 0.0490 | 0.0001 | 0.1487 |
| **Check** |  |  |  |  | **1.0000** |
| **Year 4** |  |  |  |  |  |
| **From Unrelapsed stage to** | 0.4129 | 0.0910 | 0.0000 | 0.0003 | 0.5042 |
| **From Relapsed stage to** | 0.0000 | 0.2775 | 0.0694 | 0.0002 | 0.3471 |
| **From Remitted stage to** | 0.0000 | 0.0268 | 0.1218 | 0.0001 | 0.1487 |
| **Check** |  |  |  |  | **1.0000** |
| **Year 5** |  |  |  |  |  |
| **From Unrelapsed stage to** | 0.4127 | 0.0909 | 0.0000 | 0.0003 | 0.5040 |
| **From Relapsed stage to** | 0.0000 | 0.2776 | 0.0694 | 0.0002 | 0.3472 |
| **From Remitted stage to** | 0.0000 | 0.0269 | 0.1218 | 0.0001 | 0.1488 |
| **Check** |  |  |  |  | **1.0000** |
| **Year 6** |  |  |  |  |  |
| **From Unrelapsed stage to** | 0.4280 | 0.0754 | 0.0000 | 0.0004 | 0.5038 |
| **From Relapsed stage to** | 0.0000 | 0.2777 | 0.0695 | 0.0002 | 0.3474 |
| **From Remitted stage to** | 0.0000 | 0.0223 | 0.1265 | 0.0001 | 0.1489 |
| **Check** |  |  |  |  | **1.0000** |
| **Year 7** |  |  |  |  |  |
| **From Unrelapsed stage to** | 0.4249 | 0.0783 | 0.0000 | 0.0004 | 0.5035 |
| **From Relapsed stage to** | 0.0000 | 0.2778 | 0.0695 | 0.0003 | 0.3475 |
| **From Remitted stage to** | 0.0000 | 0.0232 | 0.1257 | 0.0001 | 0.1489 |
| **Check** |  |  |  |  | **1.0000** |

^a^ The probability of all-cause mortality is gender and age-specific [SI2].

**Fig. SI1 – Materials and Methods - Markov model**

**
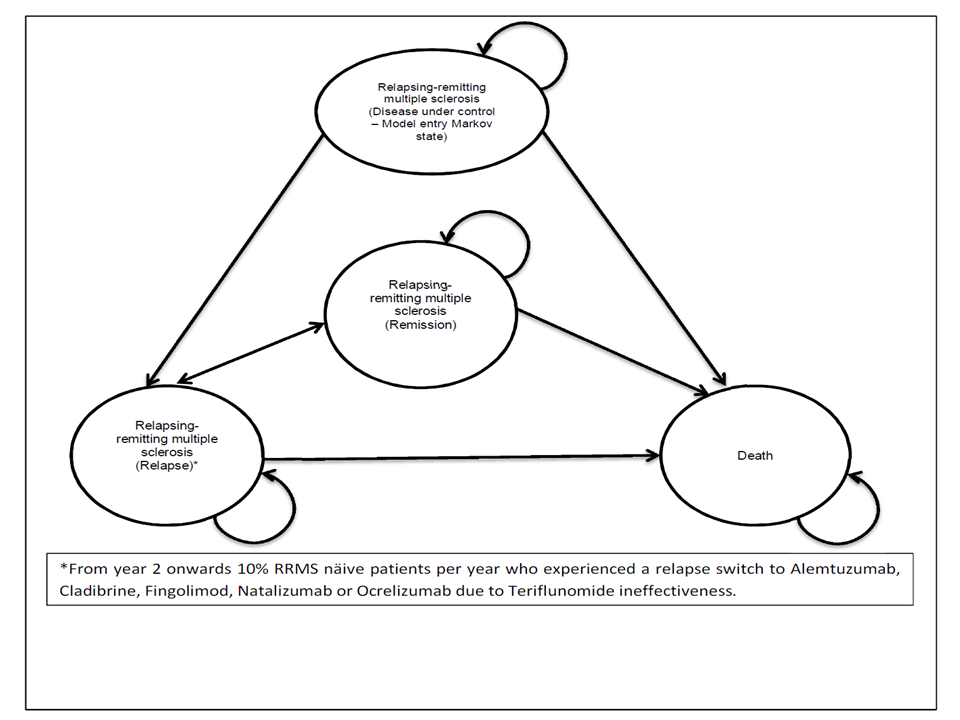
**

**Fig. SI2 – Results - SSA - Relationship between ICUR and time – Healthcare sector and societal perspective (€2019)^a^**


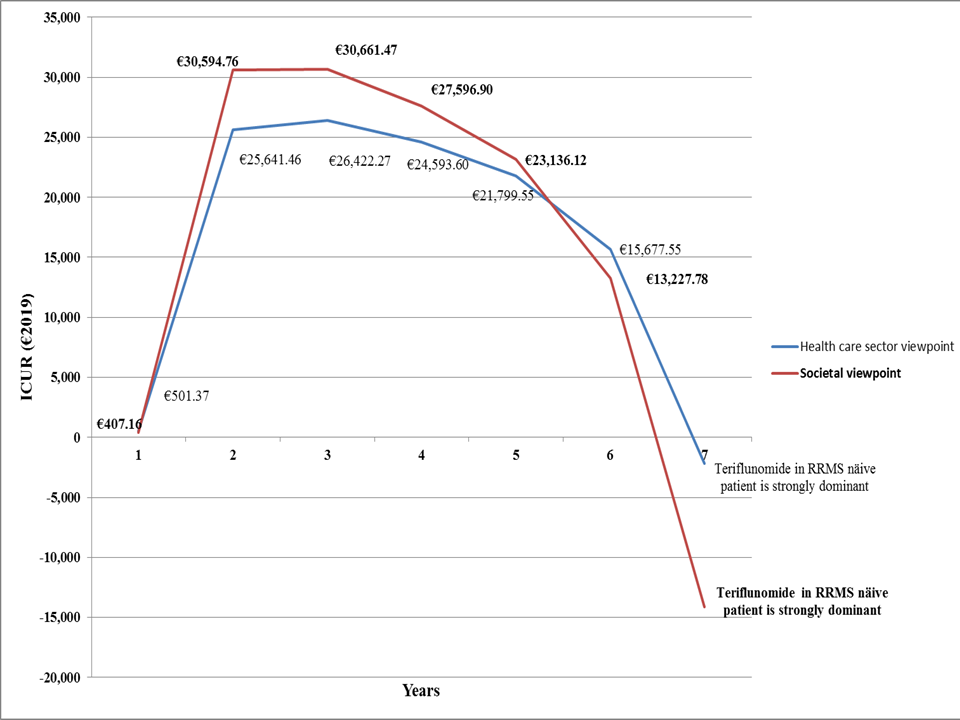


Base case ICUR: Teriflunomide in RRMS naïve patient is strongly dominant.

^a^ When ICUR>0, Teriflunomide in RRMS naïve patients is at the same time more costly and produces more QALYs vs. Teriflunomide in RRMS experienced patients.

## Section B: Cost-utility analysis and probabilistic sensitivity analysis

**Essential glossary**

**Definition 1. Cost-utility analysis (CUA):** CUA compares the difference in costs and Quality-Adjusted Life Years (QALYs) totaled by two or more healthcare programmes [SI33, SI34].

Incremental costs (ΔC) are divided by incremental QALYs (ΔQALYs) and reported via the incremental cost-utilitry ratio (ICUR). In general, the ICUR provides the healthcare decision-maker with the cost for obtaining one incremental QALY with the healthcare programme with better effect on patient’s health-related quality of life (utility) that is also more costly than the comparator(s).

More formally:

$$ICUR=\frac{\Delta C}{\Delta QALYs}$$

where:

*ΔC = (Cost_A_ – Cost_B_)*

*ΔQALYs= (*$QALYs$*_A_ –*$QALYs$*_B_)*

In order to be cost-effective, the ICUR of a given healthcare programme should be lower than a given threshold value (λ)

$$ICUR<\lambda$$

which is equivalent to:

$$\frac{\Delta C}{\Delta QALYs}<\lambda$$

**Definition 2. Net monetary benefit (NMB):** an algebraic manipulation of the incremental cost-utility ratio (ICUR) [SI1, SI33, SI34].

The NMB is simply a different way of connecting the items included in ICUR calculation:

(*NMB_A_ – NMB_B_*) = [(*λ* * $QALYs$*_A_*) – *Cost_A_*] – [(*λ* * $QALYs$*_B_*) – *Cost_B_*]

Hence, the incremental NMB (ΔNMB) is simply a different representation of the ICUR:

*ΔNMB* = [*λ* * ($QALYs$*_A_* – $QALYs$*_B_*)] – (*Cost_A_* – *Cost_B_*)

which is equivalent to:

*ΔNMB* = [(*λ* * *Δ*$QALYs$) – *ΔC*]

The reversed sign of ΔNMB inequality when compared to ICUR inequality indicates the condition for the healthcare programme under investigation to be cost-effective:

$$\Delta NMB>0$$

**Definition 3. Cost-effectiveness acceptability curve** **(CEAC):** a graphical method that summarizes the uncertainty concerning the baseline estimate of the ICUR [SI1, SI33-SI35].

A set of threshold values (e.g., from €0 to €100 000) that represent the healthcare policy makers’ willingness to pay for one ΔE unit (one ΔQALY, in our example) are reported on the x-axis, and the probability for the healthcare programme under investigation to be cost-effective (as usual bounded between 0–1) is reported on the y-axis.

For each threshold value, the healthcare decision-maker can read the probability for the healthcare programme under investigation to be cost-effective by looking across from the y-axis to the CEAC.

**Definition 4. Cost-effectiveness acceptability frontier (CEAF):** an elaboration of CEAC, the CEAF is a graphical method that represents the probability that the healthcare programme with the highest expected NMB is also cost-effective [SI1, SI33-SI35].

**SI references**

1. Briggs A, Sculpher M, Claxton K (2006) Decision modelling for health economic evaluation. Oxford University Press, Oxford
2. Sistema Statistico Nazionale - Istituto Nazionale di Statistica. Demo-Geodemo. Mappe, Popolazione, Statistiche Demografiche dell’ISTAT. Tavole di mortalità della popolazione residente. Ripartizione: Italia - Maschi e femmine - Anno: 2018. http://demo.istat.it/tvm2016/index.php. Accessed 29 December 2019 (Italian)
3. Torrinomedica S.r.l. Prontuario farmaceutico. <http://www.torrinomedica.it>. Accessed 9 December 2019 (Italian)
4. Wilkins GA. The IAU Style Manual (1989): The Preparation of Astronomical Papers and Reports. Transactions of the International Astronomical Union 1990; Series B: S23. https://www.iau.org/static/publications/stylemanual1989.pdf. Accessed 9 December 2019
5. Fattore G per Gruppo di lavoro Associazione Italiana di Economia Sanitaria (AIES). Proposta di linee guida per la valutazione economica degli interventi sanitari in Italia (2009) PharmacoEconomics – Italian Research Articles 11:83-93 (Italian). https://doi.org/10.1007/BF03320660
6. Amato MP, Battaglia MA, Caputo D, Fattore G, Gerzeli S, Pitaro M, Reggio A, Trojano M; Mu. S. I. C. Study Group. The costs of multiple sclerosis: a cross-sectional, multicenter cost-of-illness study in Italy (2002) J Neurol 249:152-163. https://doi: 10.1007/pl00007858
7. Iannazzo S, Santoni L, Saleri C, Puma E, Vestri G, Giuliani L, Canonico PL, Centonze D. Analisi di costo-efficacia dell’utilizzo di peginterferone beta-1a nel trattamento della sclerosi multipla recidivante remittente in Italia (2016) Farmeconomia. Health economics and therapeutic pathways17 (Suppl 2):13-36. (Italian). <http://dx.doi.org/10.7175/fe.v17i2S.1230>
8. Stoppe M, Busch M, Krizek L, Then Bergh F. Outcome of MS relapses in the era of disease-modifying therapy (2017) BMC Neurol 17:151. https://doi: 10.1186/s12883-017-0927-x
9. Orme M, Kerrigan J, Tyas D, Russell N, Nixon R. The effect of disease, functional status, and relapses on the utility of people with multiple sclerosis in the UK (2007) Value Health 10:54-60. https://doi: 10.1111/j.1524-4733.2006.00144.x
10. National Institute for Health and Care Excellence (NICE). Teriflunomide for treating relapsing–remitting multiple sclerosis. Technology appraisal guidance. nice.org.uk/guidance/ta303. Accessed 3 December 2019
11. Stein JD, Brown GC, Brown MM, Sharma S, Hollands H, Stein HD. The quality of life of patients with hypertension (2002) J Clin Hypertens 4:181-188. https://doi: 10.1111/j.1524-6175.2002.00970.x
12. Kolovos S, Bosmans JE, van Dongen JM, van Esveld B, Magai D, van Straten A, van der Feltz-Cornelis C, van Steenbergen-Weijenburg KM, Huijbregts KM, van Marwijk H, Riper H, van Tulder MW. Utility scores for different health states related to depression: individual participant data analysis (2017) Qual Life Res 26:1649-1658. https://doi: 10.1007/s11136-017-1536-2
13. Lazzaro C, Barone C, Caprioni F, Cascinu S, Falcone A, Maiello E, Milella M, Pinto C, Reni M, Tortora G. An Italian cost-effectiveness analysis of paclitaxel albumin (nab-paclitaxel) + gemcitabine vs gemcitabine alone for metastatic pancreatic cancer patients: the APICE study (2018) Expert Rev Pharmacoecon Outcomes Res 18:435-446. https://doi: 10.1080/14737167.2018.1464394
14. Pagano M, Gauvreau K. Principles of biostatistics, 2nd ed (2000) Duxbury Press, Pacific Grove
15. Ministero della Salute. Decreto 18 ottobre 2012. Remunerazione prestazioni di assistenza ospedaliera per acuti, assistenza ospedaliera di riabilitazione e di lungodegenza post acuzie e di assistenza specialistica ambulatoriale. (13A00528). Gazzetta Ufficiale della Repubblica Italiana, Serie Generale, n. 23, 28 January 2013. Allegato 3 Prestazioni di assistenza specialistica ambulatoriale (Italian)
16. Regione del Veneto. Giunta Regionale. Decreto n. 47 del Direttore Generale alla Sanità e al Sociale del 22 Maggio 2013. Aggiornamento, ai sensi della DGR n. 442 del 10 aprile 2013, degli Allegati A e B del Nomenclatore Tariffario Regionale dell'assistenza specialistica ambulatoriale di cui alla DGR n. 859/2011 e successive modifiche ed integrazioni. Allegati A e B. https://www.aulss3.veneto.it/index.cfm?action=mys.apridoc&iddoc=12647. Accessed 29 December 2019 (Italian)
17. Conferenza Permanente per i Rapporti tra lo Stato le Regioni e le Province Autonome di Trento e Bolzano. Accordo interregionale per la compensazione della mobilità sanitaria aggiornato all'anno 2018 - Regole tecniche. http://www.statoregioni.it/media/1809/p-1-csr-atto-rep-n-103-20giu2019.pdf. Accessed 29 December 2019 (Italian)
18. Giber Medicali. Deflussori infusionali. <https://www.gibermedicali.it/p12760-deflussore-per-infusioni-2vie-flebo-kit-sterile-senza>-ago-tubo-150-cm. Accessed 23 December 2019a (Italian)
19. Giber Medicali. Ago cannula monovia. https://www.gibermedicali.it/p14256-ago-cannula-monovia-in-pur-neo-delta-selfsafe. Accessed 23 December 2019b (Italian)
20. Regione Toscana. Ente per i Serizi Tecnico-Amministrativi di Area Vasta (ESTAV) Centro. Scheda prodotto Catetere venoso centrale ad inserimento periferico con rivestimento in argento. Firenze: ESTAV Centro, 23 Aprile 2012. http://www.osservatorioinnovazione.net/schede/sch3140.pdf. Accessed 29 December 2019 (Italian)
21. Sistema Statistico Nazionale - Istituto Nazionale di Statistica. Indice dei prezzi al consumo per l'intera collettività (base 2015=100) - dati mensili. http://dati.istat.it/. Accessed 29 December 2019 (Italian)
22. Giber Medicali. Compressa garza cotone sterile 10 x 10 cm. https://www.gibermedicali.it/p12537-compressa-garza-cotone-sterile-10-x-10-cm. Accessed 23 December 2019c) (Italian)
23. Amica Farmacia. Bioscalin. https://www.amicafarmacia.com/bioscalin.html. Accessed 9 December 2019 (Italian)
24. Lazzaro C, Bianchi C, Peracino L, Zacchetti P, Uccelli A. Economic evaluation of treating clinically isolated syndrome and subsequent multiple sclerosis with interferon beta-1b (2009) Neurol Sci 30:21-31. https://doi: 10.1007/s10072-009-0015-0
25. Sistema Statistico Nazionale - Istituto Nazionale di Statistica. Il valore della moneta in Italia dal 1861 al 2015. Coefficienti per tradurre valori monetari tra i periodi sottoindicati. Serie11_2019.Tavola 21.6.1. http://seriestoriche.istat.it/index.php?id=1&no_cache=1&tx_usercento_centofe%5Bcategoria%5D=21&tx_usercento_centofe%5Baction%5D=show&tx_usercento_centofe%5Bcontroller%5D=Categoria&cHash=2554fd1315b067d029da590365075156. Accessed 29 December 2019b (Italian)
26. Azienda Trasporti Milanesi S.p.A. tariffe sosta a pagamento - gestita da ATM. https://www.atm.it/it/ViaggiaConNoi/Auto/Documents/Discipline_sosta.pdf. Accessed 30 December 2019 (Italian)
27. Agenzia delle Entrate. Tabelle nazionali dei costi chilometrici di esercizio di autovetture e motocicli elaborate dall’ACI - Art. 3, comma 1, del decreto legislativo 2 settembre 1997, n. 314 (2018) Gazzetta Ufficiale della Repubblica Italiana, Serie Generale, n. 295 del 20 dicembre 2018, Supplemento ordinario N. 57 (Italian)
28. Agenzia delle Entrate (2013) Annuario del contribuente 2013. Rome: Agenzia delle Entrate, Rome (Italian)
29. Ente Bilaterale Colf e Badanti. Contratto collettivo nazionale di lavoro colf e badanti 2017-2020. www.ebilcoba.it. Accessed 27 December 2019 (Italian)
30. Istituto Nazionale per la Previdenza Sociale. Direzione Centrale Entrate. Circolare n. 16. Importo dei contributi dovuti per l’anno 2019 per i lavoratori domestici. https://www.inps.it/CircolariZIP/Circolare%20numero%2016%20del%2001-02-2019.pdf. Accessed 29 December 2019a (Italian)
31. Banca d’Italia. I bilanci delle famiglie italiane nell’anno 2016. tav.S28. https://www.bancaditalia.it/statistiche/tematiche/indagini-famiglie-imprese/bilanci-famiglie/risultati-indagine/index.html. Accessed 29 December 2019 (Italian)
32. Istituto Nazionale per la Previdenza Sociale. Canale informazioni. Minimali giornalieri di retribuzione. https://www.inps.it/NuovoportaleINPS/default.aspx?iprestazioni=110&iTema=7688. (2019, Accessed 29 December 2019b (Italian)
33. Drummond MF, Sculpher MJ, Claxton K, Stoddart GL, Torrance, GW. Methods for the economic evaluation of health care programmes, 4th ed (2015) Oxford University Press, Oxford
34. Neumann PJ, Ganiats TG, Russell LB, Sanders GD, Siegel JE, editors. Cost-effectiveness in health and medicine, 2nd ed (2016) Oxford University Press, New York
35. Fenwick E, Claxton K, Sculpher M. Representing uncertainty: the role of cost-effectiveness acceptability curves (2001) Health Econ 10:779-787. https://doi: 10.1002/hec.635
